# TGF-**β** coordinates alanine synthesis and import for myofibroblast differentiation in pulmonary fibrosis

**DOI:** 10.1172/jci.insight.199449

**Published:** 2026-04-23

**Authors:** Fei Li, Niv Vigder, David R. Ziehr, Mari Kamiya, Hung N. Nguyen, Diana E. Ferreyra Faustino, Aseel H. Khalil, Hilaire C. Lam, Matthew L. Steinhauser, Edy Y. Kim, William M. Oldham

**Affiliations:** 1 Department of Medicine, The Warren Alpert Medical School of Brown University, Providence, Rhode Island, USA.; 2 Department of Medicine, Brigham and Women’s Hospital and Harvard Medical School, Boston, Massachusetts, USA.; 3 School of Life and Environmental Sciences, Faculty of Science, The University of Sydney, Sydney, New South Wales, Australia.; 4 Metabolomics Platform, The Broad Institute of MIT and Harvard, Cambridge, Massachusetts, USA.; 5 Department of Medicine, Massachusetts General Hospital and Harvard Medical School, Boston, Massachusetts, USA.; 6 Department of Medicine, University of Pittsburgh Medical Center, Pittsburgh, Pennsylvania, USA.; 7 Aging Institute, University of Pittsburgh School of Medicine, Pittsburgh, Pennsylvania, USA.

**Keywords:** Cell biology, Metabolism, Pulmonology, Amino acid metabolism, Fibrosis, Metabolomics

## Abstract

Idiopathic pulmonary fibrosis (IPF) is a progressive interstitial lung disease driven by aberrant fibroblast-to-myofibroblast differentiation, which requires metabolic reprogramming. Here, we identify alanine as an essential metabolite for myofibroblast differentiation. TGF-β increases intracellular alanine levels through enhanced synthesis and import in both normal and IPF lung fibroblasts. Alanine synthesis is primarily mediated by glutamate-pyruvate transaminase 2 (GPT2), whose expression is regulated by the glutamine/glutamate/α-ketoglutarate axis. Inhibition of GPT2 depletes alanine and suppresses TGF-β–induced α-SMA and COL1A1 expression, which are rescued by exogenous alanine. We also identify solute carrier family 38 member 2 (SLC38A2) as a transporter for both alanine and glutamine, upregulated by TGF-β or alanine deprivation. SLC38A2 and GPT2 form a coordinated regulatory axis sustaining intracellular alanine levels to support myofibroblast differentiation. Mechanistically, alanine deficiency impairs glycolytic flux and depletes tricarboxylic acid cycle intermediates, while alanine supplementation provides carbon and nitrogen for intracellular glutamate and proline biosynthesis, particularly under glutamine deprivation. Combined inhibition of alanine synthesis and uptake suppresses fibrogenic responses in fibroblasts and human precision-cut lung slices, highlighting dual metabolic targeting as a potential therapeutic strategy for fibrotic lung disease.

## Abstract 2

In pulmonary fibrosis, scar-forming cells rely on alanine as a metabolic building block. Simultaneously blocking its synthesis and uptake suppresses fibrosis in human lung tissue.

## Graphical abstract

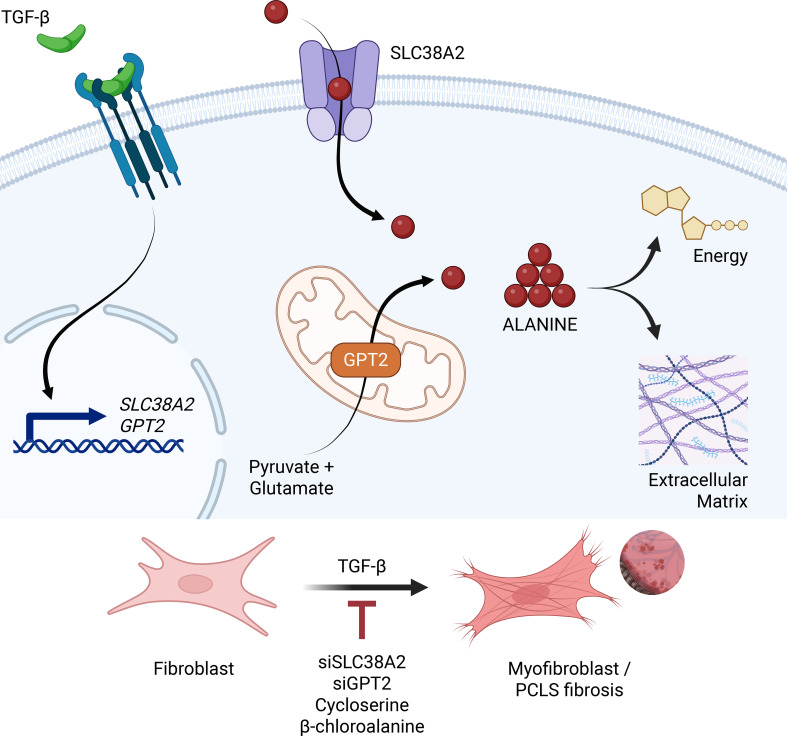

## Introduction

Idiopathic pulmonary fibrosis (IPF) is a progressive and lethal lung disease with a median survival of 3–5 years after diagnosis (1). Antifibrotic therapies, including pirfenidone and nintedanib, are limited to slowing disease progression (2, 3). More recently, nerandomilast has been approved as a novel therapeutic option for IPF, further expanding the treatment landscape (4). Nevertheless, no currently available pharmacological therapies reduce mortality or cure the disease (5). Thus, a deeper understanding of underlying disease mechanisms is essential for the development of more effective antifibrotic therapies.

Fibroblast-to-myofibroblast differentiation is a key step in fibrosis progression (6). Under the influence of profibrotic factors such as TGF-β, pulmonary fibroblasts differentiate to myofibroblasts characterized by metabolic reprogramming, including increased nutrient uptake and activation of anabolic pathways that promote extracellular matrix deposition and contractile force generation (7). Recent studies have shown that myofibroblast differentiation requires changes in glucose and glutamine metabolic pathways (8). TGF-β promotes the uptake of extracellular glucose and its subsequent metabolism into glycine and lactate, supporting collagen synthesis and bioenergetic pathway activity, respectively (9, 10). Our previous studies further showed that lactate transporters MCT1 and MCT4 promote myofibroblast differentiation and represent actionable metabolic targets (11).

Similarly, glutamine metabolism contributes to the collagen deposition that drives pathological tissue remodeling in fibrosis (12–14). Glutamine is a vital nutrient for rapidly proliferating cells as it supports metabolic and biosynthetic reactions (15, 16). Strategies targeting glutamine metabolism are emerging for the treatment of IPF, with glutaminase identified as a potential therapeutic target for fibrosis (12, 17). However, cancer-related studies have revealed that targeting glutamine metabolism showed limited efficacy in vivo due to metabolic adaptability that enables cells to bypass glutamine-dependent metabolic requirements for proliferation (18). This metabolic flexibility may involve compensatory pathways mediated by transaminases and alternative metabolites, including glutamate, asparagine, serine, α-ketoglutarate (α-KG), and pyruvate (19–23). In particular, the reversible synthesis and interconversion of nonessential amino acids (NEAAs) represent a key mechanism underlying this metabolic flexibility under conditions of nutrient stress. Additionally, although NEAAs (e.g., glutamine, serine, and alanine) can be synthesized intracellularly, the tight regulation of their intracellular concentration depends largely on extracellular sources, particularly when synthesis is limited (24, 25). Through import and export, the extracellular metabolite pool offers the buffering capacity that is required for cells to maintain a narrow, homeostatic range of metabolite concentrations intracellularly. The reliance on extracellular metabolite pools also suggests that intracellular synthesis alone is insufficient to meet the metabolic demands of activated fibroblasts. For example, targeted inhibition of the activity of key transaminases does not completely inhibit myofibroblast differentiation (8), suggesting that cells can compensate for impaired synthesis by increasing the uptake of extracellular metabolites. Such metabolic adaptability, characterized by flexible utilization of both intracellular and extracellular metabolite sources, enables myofibroblasts to tolerate large fluctuations in nutrient availability and limits the efficacy of metabolism-targeted antifibrotic therapies.

Prompted by these considerations, we investigated whether and how specific NEAAs act as compensatory substrates during myofibroblast differentiation. We demonstrated that alanine provision, through de novo synthesis by glutamate-pyruvate transaminase 2 (GPT2) and import by solute carrier family 38 member 2 (SLC38A2), is essential for myofibroblast differentiation. Combined pharmacological inhibition of both alanine synthesis and uptake significantly suppressed this process, revealing a previously unrecognized metabolic vulnerability and a potentially effective dual-targeting therapeutic strategy for IPF.

## Results

### TGF-β–induced myofibroblast differentiation increases intracellular alanine.

To examine how medium composition affects myofibroblast differentiation, we treated primary normal human lung fibroblasts (NHLFs) with TGF-β (2 ng/mL) in 1 of 2 commonly used fibroblast culture media, DMEM or fibroblast basal medium (FBM). DMEM is a general-purpose medium containing glutamine and branched-chain amino acids (valine, leucine, and isoleucine) but lacking NEAAs (alanine, proline, glutamate, asparagine, and aspartate) (Supplemental Table 1; supplemental material available online with this article; https://doi.org/10.1172/jci.insight.199449DS1). FBM, a fibroblast-specific medium, has a more complex proprietary amino acid composition that contains NEAAs (Supplemental Figure 1, A–E). Thus, in DMEM, fibroblasts rely on endogenous synthesis of these amino acids, enabling the interrogation of synthesis-dependent metabolic programs, whereas in FBM, extracellular availability permits the assessment of uptake-permissive metabolic remodeling.

**Figure 1:**
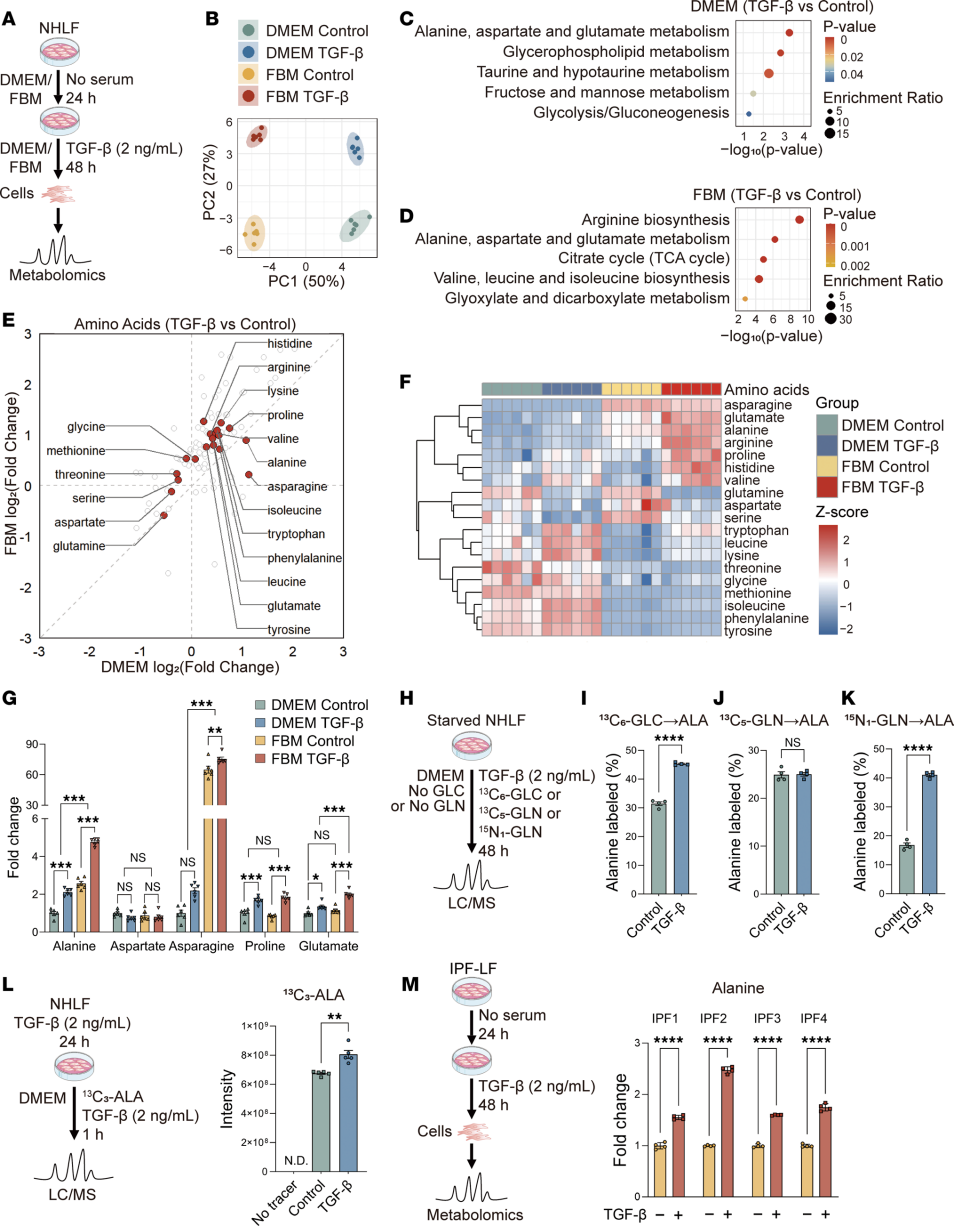
Increased intracellular alanine levels during myofibroblast differentiation. (**A**) Schematic overview of the experimental design for primary normal human lung fibroblasts (NHLFs) treated with TGF-β (2 ng/mL) in DMEM or FBM. Samples were collected and analyzed after 48 hours using LC/MS (*n* = 6 for each condition). (**B**) Principal component analysis (PCA) of the experimental groups. (**C** and **D**) Enriched metabolite sets after 48 hours of TGF-β treatment in DMEM (**C**) and FBM (**D**), based on differential metabolites with fold-change ≥ 1.5 and *P* ≤ 0.05. Enrichment ratio = hits / expected. (**E**) Comparison of intracellular amino acid changes induced by TGF-β treatment in DMEM versus FBM conditions. (**F**) Heatmap showing the intracellular levels of amino acids in fibroblasts cultured in DMEM or FBM with or without TGF-β stimulation for 48 hours. Each column represents a sample, and each row corresponds to an amino acid. Colors indicate *z* score–normalized abundance, with red and blue representing higher and lower relative levels, respectively. (**G**) Relative quantification of the intracellular abundances of alanine, aspartate, asparagine, proline, and glutamate. (**H**) Schematic overview of LC/MS-based stable isotope tracing with [U-^13^C_6_]-glucose (^13^C_6_-GLC), [U-^13^C_5_]-glutamine (^13^C_5_-GLN), or[α-^15^N_1_]-glutamine (^15^N_1_-GLN) for 48 hours. (**I–K**) Stable isotope label enrichment of alanine from ^13^C_6_-GLC (**I**), ^13^C_5_-GLN (**J**), or ^15^N_1_-GLN (**K**). (**L**) Schematic overview of short-term uptake assays using isotope tracing with [U-^13^C_3_]-alanine (^13^C_3_-ALA) for 1 hour (left). Signal intensity of intracellular ¹³C_3_-alanine (undetectable without tracer) (right). (**M**) Schematic overview of the experimental design for IPF human lung fibroblasts (IPF-LF) from 4 donors treated with TGF-β (2 ng/mL) in FBM for 48 hours (left). Relative quantification of intracellular alanine levels (right). For **G**, 1-way ANOVA; **I–M**, unpaired 2-tailed *t* test. Data are presented as mean ± SEM. ns, *P* > 0.05; *P < 0.05; **P < 0.01; ***P < 0.001; ****P < 0.0001.

We next performed liquid chromatography–high-resolution accurate mass spectrometry (LC/MS) to profile the intracellular metabolome of NHLFs after 48 hours of TGF-β treatment (Figure 1A). Principal component analysis (PCA) of approximately 100 measured metabolites revealed a clear separation among experimental groups, with the first principal component (50% of variance) driven by medium composition and the second principal component (27% of variance) reflecting the effects of TGF-β treatment (Figure 1B).

Metabolite set enrichment analyses revealed an upregulation of amino acid metabolism after TGF-β treatment in DMEM and FBM (Figure 1, C and D). The concentrations of numerous amino acids were differentially regulated by TGF-β, regardless of culture medium. Notably, intracellular glutamine was decreased, potentially reflecting increased glutamine consumption, whereas levels of several NEAAs, including proline, alanine, and glutamate, were increased (Figure 1E). Comparison of DMEM- and FBM-cultured cells revealed baseline and TGF-β–induced differences in metabolite profiles, highlighting that medium composition can shape cellular metabolism and influence responses to profibrotic stimuli (Figure 1F). When cells were deprived of an exogenous supply of the 5 NEAAs in DMEM culture, TGF-β increased the intracellular concentrations of alanine, proline, and glutamate (Figure 1G), suggesting enhanced intracellular synthesis. Among these, the intracellular concentration of alanine, irrespective of TGF-β treatment, was significantly higher in FBM, suggesting that the intracellular level of alanine is regulated, at least in part, by the extracellular alanine pool (Figure 1G).

Given the observed increases in alanine after TGF-β treatment, we next sought to determine the metabolic origins of alanine, as determined by LC/MS-based stable isotope tracing experiments using [U-^13^C_6_]-glucose, [U-^13^C_5_]-glutamine, or [α-^15^N_1_]-glutamine (Figure 1H). [U-^13^C_6_]-glucose tracing confirmed that glucose serves as a key carbon contributor to alanine synthesis (Figure 1I), consistent with previous studies (26). TGF-β treatment further increased the fraction of glucose-labeled alanine from approximately 30% to approximately 45% (Figure 1I). We found that approximately 25% and approximately 20% of the total alanine pool was labeled from [U-^13^C_5_]-glutamine and [α-^15^N_1_]-glutamine, respectively, and that TGF-β further increased the fraction of ^15^N-alanine labeling from ^15^N_1_-glutamine to approximately 40% (Figure 1, H, J, and K). Taken together, these data suggest that, apart from glucose, glutamine is a critical carbon and nitrogen source for alanine synthesis under both baseline conditions and TGF-β treatment.

To quantify alanine uptake, we conducted short-term (1 hour) labeling experiments using [U-^13^C_3_]-alanine in control and TGF-β–pretreated cells in DMEM (Figure 1L). Labeled intracellular alanine was detected under both baseline and TGF-β treatment conditions, with TGF-β enhancing its accumulation (Figure 1L).

To determine whether the observed alterations in alanine metabolism are preserved in a disease context, we cultured primary fibroblasts isolated from 4 patients with IPF in FBM, which approximates the nutrient-replete in vivo environment, and confirmed that TGF-β significantly increased alanine levels intracellularly by approximately 1.5–2.5 fold (Figure 1M), consistent with previous studies reporting higher alanine levels in the lungs of patients with IPF (27, 28). These findings indicate that TGF-β–induced alanine accumulation is not restricted to normal fibroblasts under defined nutrient conditions but is conserved in primary IPF fibroblasts, supporting the relevance of this metabolic response in pulmonary fibrosis.

### Glutamine promotes TGF-β–induced GPT2 expression to support myofibroblast differentiation.

**Figure 2:**
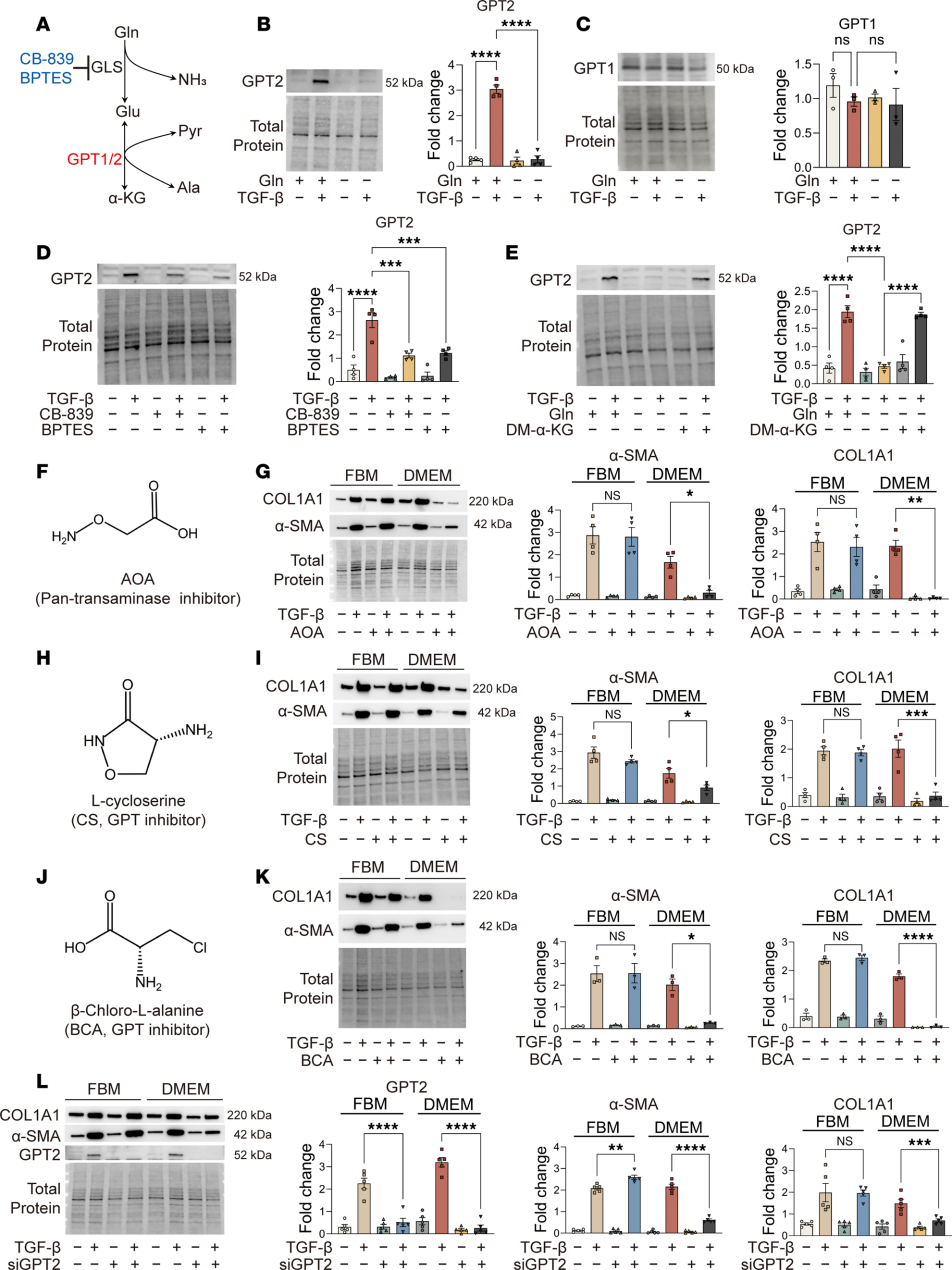
Glutamine promotes TGF-β–induced GPT2 expression to support myofibroblast differentiation. (**A**) Schematic overview of glutamine catabolism and alanine synthesis involving GPT1 and GPT2. (**B** and **C**) Western blot analysis and quantification of GPT2 (**B**) and GPT1 (**C**) in NHLFs treated with TGF-β in glutamine-deficient or glutamine-sufficient (2 mM) DMEM for 48 hours. (**D** and **E**) Western blot analysis and quantification of GPT2 in NHLFs treated with TGF-β in DMEM for 48 hours with glutaminase-1 inhibitors (CB-839, 10 μM; BPTES, 5 μM) (**D**) or with/without DM-α-KG (5 mM) under glutamine-deficient or -sufficient conditions (**E**). (**F**) Chemical structure of the pan-transaminase inhibitor AOA. (**G**) NHLFs were stimulated with TGF-β in FBM or DMEM, with or without AOA (1 mM) for 48 hours. α-SMA and COL1A1 expression were examined by Western blot. (**H**) Chemical structure of the GPT1/2 inhibitor CS. (**I**) NHLFs were stimulated with TGF-β in FBM or DMEM, with or without CS (100 μM) for 48 hours. α-SMA and COL1A1 expression were examined by Western blot. (**J**) Chemical structure of the GPT1/2 inhibitor BCA. (**K**) NHLFs were stimulated with TGF-β in FBM or DMEM, with or without BCA (100 μM) for 48 hours. α-SMA and COL1A1 expression were examined by Western blot. (**L**) NHLFs were transfected with control siRNA or GPT2 siRNA for 24 hours, followed by 24 hours of starvation. Cells were then stimulated with TGF-β in FBM or DMEM for 48 hours. GPT2-knockdown efficiency, α-SMA, and COL1A1 expression were examined by Western blot. Individual data points represent biological replicates. For **B–E**, 1-way ANOVA; **G**, **I**, **K**, and **L**, 1-way ANOVA versus TGF-β within each medium. ns, *P* > 0.05; *P < 0.05; **P < 0.01; ***P < 0.001; ****P < 0.0001.

Alanine synthesis is mediated by GPTs. In this process, glutamine is converted to glutamate by glutaminase. Then, GPT1/2 enzymes catalyze glutamate transamination with pyruvate to generate alanine and α-KG (Figure 2A). Our [α-^15^N_1_]-glutamine tracing data demonstrated a 2.5-fold increase in transaminase activity after TGF-β treatment (Figure 1K). To specifically examine intracellular alanine synthesis in the absence of exogenous alanine, we measured cytosolic GPT1 and mitochondrial GPT2 protein expression during TGF-β–induced myofibroblast differentiation in DMEM. TGF-β increased GPT2 expression but had no effect on GPT1 (Figure 2, B and C).

To determine the role of GPT2 in myofibroblast differentiation, we first examined the effects of TGF-β in medium lacking glutamine (Gln), a primary source of glutamate for GPT2-catalyzed transamination of pyruvate to alanine. Interestingly, TGF-β did not increase GPT2 expression in the absence of glutamine (Figure 2B). Because SLC1A5 is a major glutamine transporter implicated in fibroblast activation (29), we next evaluated its role using both siRNA-mediated knockdown and pharmacological inhibition with V-9302. SLC1A5 knockdown significantly reduced GPT2 protein expression under TGF-β stimulation (Supplemental Figure 2A). Consistently, V-9302 treatment also decreased TGF-β–induced GPT2 levels (Supplemental Figure 2B), indicating that glutamine transport contributes to GPT2 regulation. Similarly, the glutaminase-1 inhibitors telaglenastat (CB-839) and BPTES suppressed TGF-β–induced upregulation of GPT2 protein expression in glutamine-containing medium (Figure 2D), despite discrepancies with previous studies (19, 30). Additionally, glutamine deficiency may decrease protein expression by impairing translation or stability, a process that can be rescued by α-KG (31). Indeed, we found that supplementation with a cell-permeable analogue of α-KG [dimethyl (DM)-α-KG] enabled the TGF-β–induced upregulation of GPT2 protein expression even in the absence of glutamine (Figure 2E). Taken together, these data suggest that a glutamine/glutamate/α-KG metabolic axis mediates TGF-β–induced upregulation of GPT2.

We next examined direct inhibition of GPT2 by small molecules to investigate its role in TGF-β–induced myofibroblast differentiation. We first used the pan-transaminase inhibitor, aminooxyacetic acid (AOA) (32), which has been shown previously to block α-SMA and collagen production during myofibroblast differentiation (13). Unexpectedly, AOA did not inhibit TGF-β–mediated upregulation of α-SMA and COL1A1 in FBM, although it was effective in DMEM as previously demonstrated (Figure 2, F and G) (13). We then tested the effects of the GPT1/2 inhibitors L-cycloserine (CS) and β-chloro-l-alanine (BCA) (33). Like AOA, both CS and BCA suppressed TGF-β–induced upregulation of α-SMA and COL1A1 protein expression in DMEM but had no effect in FBM (Figure 2, H–K, and Supplemental Figure 2, C–F). Given that CS and BCA lack selectivity for individual GPT isoforms (i.e., GPT1 vs. GPT2), we next used RNA interference to study the role of each isoform separately. GPT1 knockdown had no effect on myofibroblast differentiation in either FBM or DMEM, as assessed by the relative protein expression of COL1A1 and α-SMA (Supplemental Figure 2G). By contrast, GPT2 knockdown significantly decreased the TGF-β–induced upregulation of α-SMA and COL1A1 protein expression in DMEM (Figure 2L). Consistently, GPT2 knockdown also decreased *ACTA2* (encoding α-SMA), *COL1A1*, *FN1* (encoding fibronectin), and *CTGF* mRNA, further supporting suppression of the profibrotic program at a transcriptional level (Supplemental Figure 2H). Notably, GPT2 knockdown did not affect the activation of Smad3 signaling measured 1 hour after TGF-β treatment (Supplemental Figure 2I). Together, these results suggest that GPT2 is required for myofibroblast differentiation only under NEAA-deficient conditions (i.e., DMEM). In contrast, when exogenous NEAAs are available (i.e., FBM), GPT2 is redundant with respect to TGF-β–induced myofibroblast differentiation.

### Alanine is essential for α-SMA expression and collagen production in myofibroblasts.

**Figure 3:**
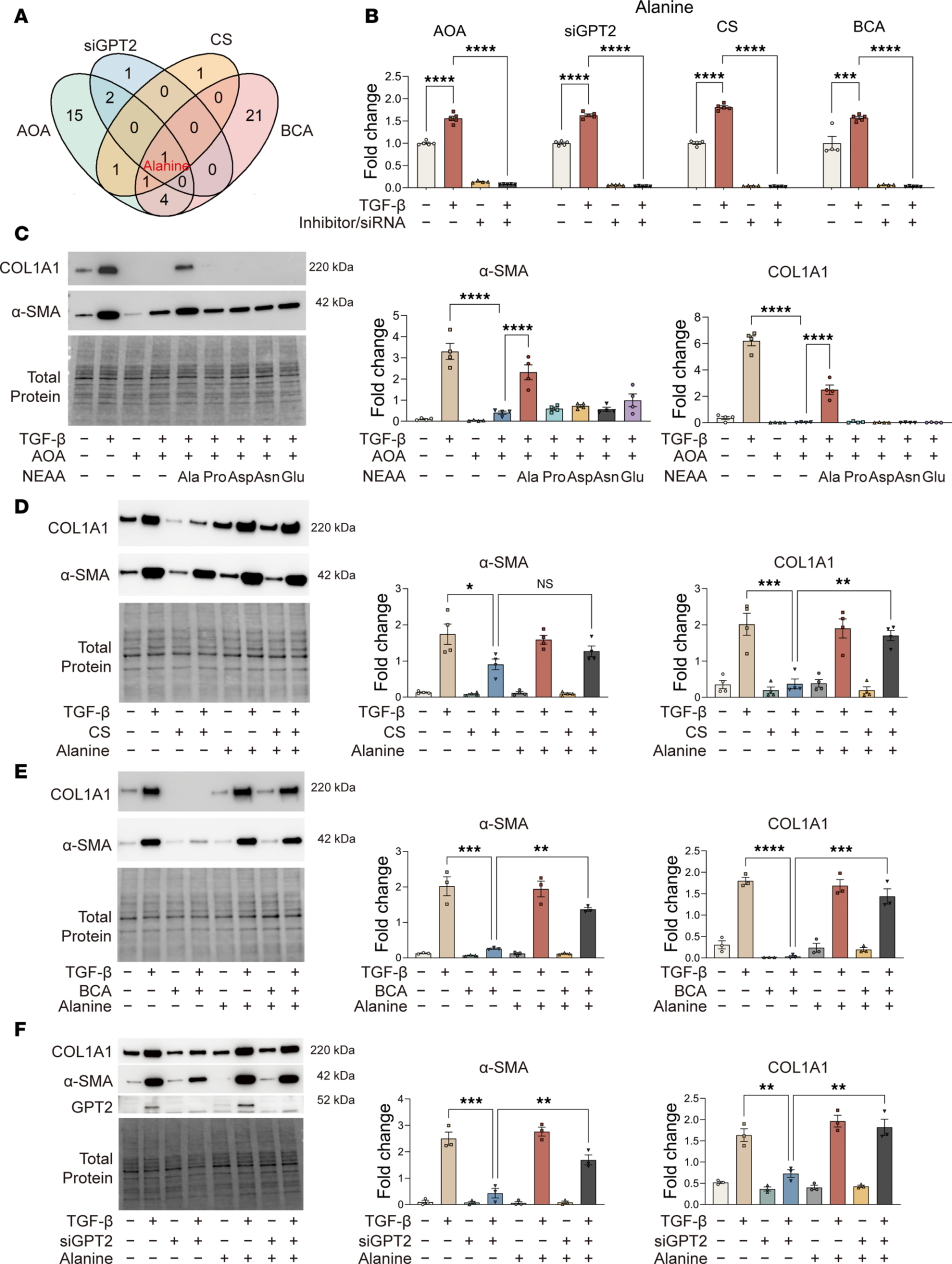
Alanine is essential for myofibroblast differentiation. (**A**) Venn diagram depicting the number of differentially regulated metabolites across 4 treatment conditions compared with their respective control groups, with alanine as the only shared metabolite. Differential metabolites: fold-change ≥ 1.5 and *P* ≤ 0.05. (**B**) Intracellular alanine levels in NHLFs treated with TGF-β for 48 hours, with or without GPT2 knockdown or inhibition in DMEM. (**C**) Western blot analysis and quantification of α-SMA and COL1A1 expression in NHLFs treated with AOA (1 mM) and TGF-β for 48 hours in DMEM, with or without individual NEAA supplementation (2 mM each). (**D**) NHLFs were stimulated with TGF-β in DMEM for 48 hours, with or without CS (100 μM) and alanine supplementation (2 mM). α-SMA and COL1A1 expression were examined by Western blot. (**E**) NHLFs were stimulated with TGF-β in DMEM for 48 hours, with or without BCA (100 μM) and alanine supplementation (2 mM). α-SMA and COL1A1 expression were examined by Western blot. (**F**) NHLFs were transfected with control siRNA or GPT2 siRNA for 24 hours, followed by 24 hours of starvation. Cells were then stimulated with TGF-β in DMEM for 48 hours, with or without alanine supplementation. α-SMA and COL1A1 expression were examined by Western blot. For Western blot, individual data points represent biological replicates. **B** versus TGF-β and **C–F** versus knockdown/inhibitor plus TGF-β were analyzed using 1-way ANOVA. Data are presented as mean ± SEM. ns, *P* > 0.05; *P < 0.05; **P < 0.01; ***P < 0.001; ****P < 0.0001.

Next, we investigated whether the role of GPT2 in supporting myofibroblast differentiation depends on alanine metabolism. To this end, we analyzed intracellular metabolite profiles after either genetic knockdown or pharmacological inhibition of GPT2 in DMEM-cultured fibroblasts. In the absence of TGF-β, AOA and BCA treatment markedly altered the intracellular concentration of 24 and 27 metabolites, respectively, while both CS and GPT2 knockdown separately led to differential regulation of 4 metabolites (Figure 3A). Alanine was the only metabolite that was consistently downregulated by each of the 4 treatments (Figure 3, A and B). Notably, after TGF-β stimulation, alanine remained the most prominently altered amino acid across all treatment groups (Supplemental Figure 3, A–D).

To determine whether alanine deficiency impairs myofibroblast differentiation, we performed rescue experiments by supplementing 2 mM exogenous alanine, as previously reported (26, 34, 35). Since DMEM lacks 5 NEAAs, we compared alanine with the 4 other NEAAs. Alanine — but not proline, aspartate, asparagine, or glutamate — significantly reversed the inhibitory effect of AOA on TGF-β–induced α-SMA and COL1A1 protein upregulation (Figure 3C). Similarly, alanine restored TGF-β–induced upregulation of α-SMA and COL1A1 that was suppressed by CS (Figure 3D), BCA (Figure 3E), or GPT2 knockdown (Figure 3F). These data demonstrate that alanine is required for myofibroblast differentiation and can bypass the antifibrotic effects of GPT2 inhibition.

### SLC38A2 facilitates alanine uptake to support myofibroblast differentiation.

**Figure 4:**
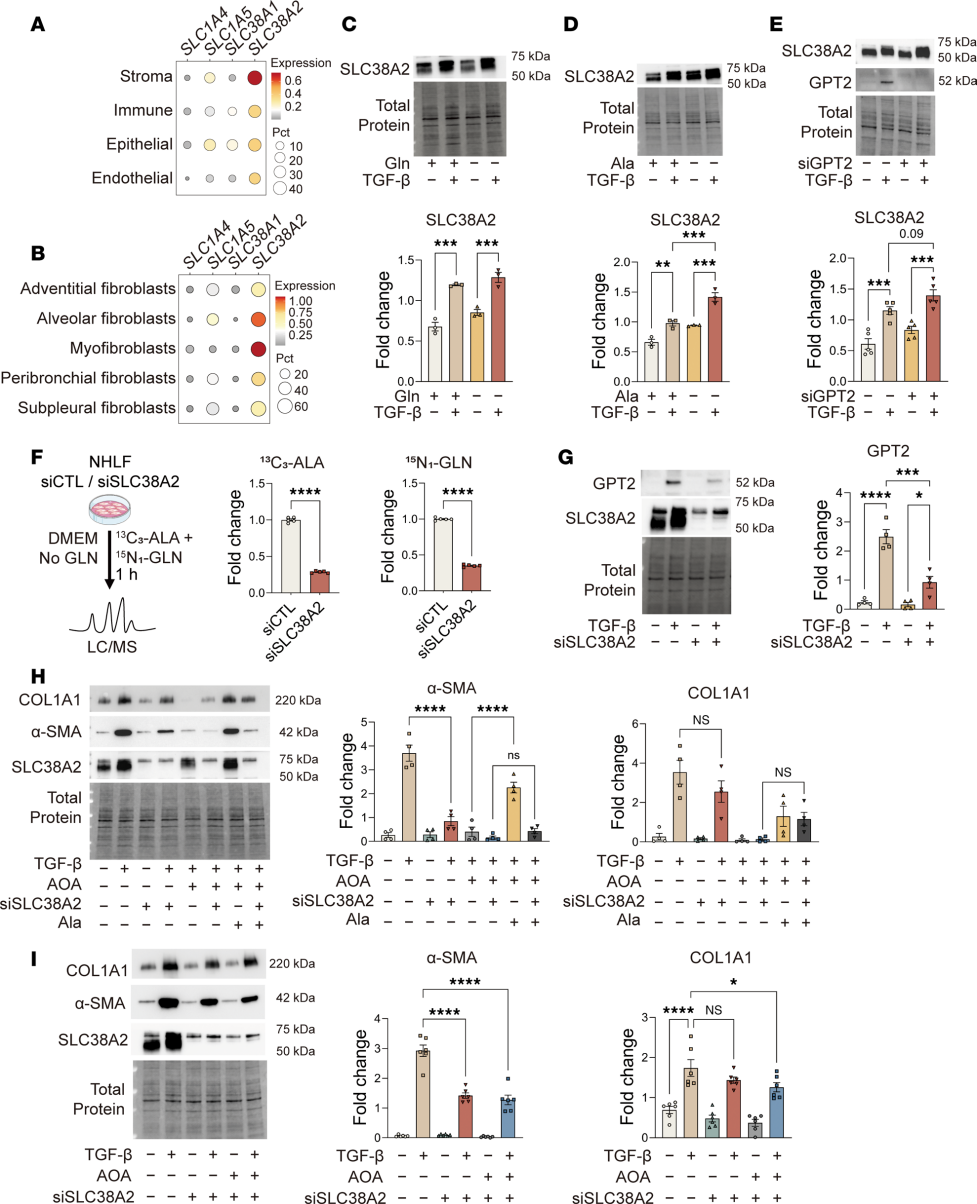
SLC38A2 mediates alanine uptake and myofibroblast differentiation. (**A**) Normalized transporter expression in lung cell types from the Human Lung Cell Atlas. (**B**) Fibroblast subset–specific expression in healthy lung samples. Dot color indicates mean expression; size indicates percentage of expressing cells. (**C**) Western blot analysis and quantification showing that TGF-β significantly upregulates SLC38A2 protein expression in NHLFs, independent of glutamine availability. (**D**) Western blot analysis and quantification showing that extracellular alanine deprivation promotes SLC38A2 protein expression in DMEM-cultured fibroblasts. (**E**) Western blot analysis and quantification showing that GPT2 knockdown reduces intracellular alanine levels and shows a trend toward increasing SLC38A2 protein expression in FBM. (**F**) Schematic overview of the short-term [U-^13^C_3_]-alanine-alanine and [^15^N_1_]-glutamine co-uptake assay in control and SLC38A2-knockdown fibroblasts and relative quantification of ^13^C_3_-alanine and [^15^N_1_]-glutamine uptake. (**G**) Western blot analysis and quantification showing that SLC38A2 knockdown reduces TGF-β–induced GPT2 expression in FBM. (**H**) Western blot analysis and quantification of α-SMA and COL1A1 expression in control and SLC38A2-knockdown fibroblasts cultured in DMEM for 48 hours with TGF-β stimulation, with or without AOA (1 mM) and/or alanine supplementation (2 mM). (**I**) Western blot analysis and quantification of α-SMA and COL1A1 expression in FBM-cultured fibroblasts after TGF-β stimulation and combined treatment with AOA and SLC38A2 knockdown for 48 hours. For Western blot analysis, individual data points represent biological replicates. For **F**, unpaired 2-tailed *t* test; for **C–E** and **G–I**, 1-way ANOVA. Data are presented as mean ± SEM. ns, *P* > 0.05; *P < 0.05; **P < 0.01; ***P < 0.001; ****P < 0.0001.

Given that inhibition of intracellular alanine synthesis did not impair myofibroblast differentiation in alanine-replete FBM, we hypothesized that cells may regulate the intracellular alanine pool by increasing uptake of alanine from the medium. Alanine uptake is mediated by the SLC family of transporters, with previous studies implicating ASCT1 (*SLC1A4*), ASCT2 (*SLC1A5*), SNAT1 (*SLC38A1*), and SNAT2 (*SLC38A2*), though SLC1A5 has been reported to have minimal impact on alanine transport (36–38). Compared with other transporters, single-cell transcriptomic data revealed that *SLC38A2* was highly expressed in stromal cells, particularly in fibroblasts and myofibroblasts (Figure 4, A and B). Despite previous findings indicating that *SLC38A2* mRNA levels are not altered in NHLFs treated with TGF-β for 48 hours (29), our results demonstrated that TGF-β significantly upregulated SLC38A2 protein expression (Figure 4C). Similar to TGF-β alone, a profibrotic cocktail including TGF-β, PDGF-AB, TNF-α, and LPA also induced GPT2 and SLC38A2 expression and increased intracellular alanine levels (Supplemental Figure 4, A and B) (39). Unlike GPT2, the TGF-β–induced upregulation of SLC38A2 protein expression was independent of extracellular glutamine (Figure 4C). Previous studies have shown that SLC38A2 protein expression is induced in response to amino acid deprivation (40). We further discovered that adding alanine to DMEM decreased SLC38A2 protein expression in control and TGF-β–treated cells (Figure 4D). GPT2 knockdown, which decreases intracellular alanine, increased SLC38A2 modestly in FBM (Figure 4E), suggesting a compensatory upregulation of alanine transport in response to impaired intracellular alanine synthesis.

To determine the relative contribution of SLC38A2 to alanine uptake, we performed short-term uptake assays using [U-^13^C_3_]-alanine in control and SLC38A2-knockdown fibroblasts (Figure 4F). SLC38A2 knockdown decreased alanine import by approximately 3-fold, confirming that the majority of alanine uptake is mediated by SLC38A2 (Figure 4F). Interestingly, glutamine uptake was also suppressed by approximately 50%, confirming the previously reported role of SLC38A2 as a glutamine transporter (Figure 4F) (41). In line with these findings, SLC38A2 knockdown reduced intracellular alanine and glutamine levels by approximately 3- and 2-fold, respectively, after 48 hours in FBM medium (Supplemental Figure 4C). Notably, glutamine import decreased by 30% when cells were supplemented with 2 mM alanine (Supplemental Figure 4D), consistent with prior reports of alanine-mediated competitive inhibition of glutamine uptake (35). Given that SLC38A2 functions both as an alanine and glutamine transporter, and that glutamine regulates GPT2 protein expression (Figure 2B), we hypothesized that SLC38A2 knockdown would further suppress GPT2 expression, and thereby reduce alanine synthesis. As hypothesized, SLC38A2 knockdown significantly attenuated TGF-β–induced GPT2 upregulation in FBM-cultured cells (Figure 4G).

We next explored the role of SLC38A2 in myofibroblast differentiation. In DMEM, SLC38A2 knockdown decreased TGF-β–induced upregulation of α-SMA protein expression (Figure 4H), possibly by limiting glutamine availability, and thereby reducing GPT2-dependent alanine synthesis. This inhibitory effect of SLC38A2 knockdown was potentiated when alanine synthesis was fully blocked by AOA (Figure 4H). Notably, alanine supplementation restored α-SMA protein expression in control cells, but not in SLC38A2-knockdown cells, confirming that SLC38A2 is required for alanine uptake–mediated α-SMA protein upregulation (Figure 4H). COL1A1 protein expression was only modestly affected by SLC38A2 knockdown (Figure 4H). In FBM, SLC38A2 knockdown significantly reduced *ACTA2* (encoding α-SMA) expression and modestly decreased *COL1A1* mRNA levels, whereas other profibrotic markers were not significantly altered (Supplemental Figure 4E). Consistent with these transcriptional changes, SLC38A2 knockdown attenuated TGF-β–induced α-SMA protein expression, whereas COL1A1 protein levels were less markedly reduced (Figure 4I), suggesting differential sensitivity of these profibrotic markers to SLC38A2-mediated metabolic regulation. To eliminate intracellular alanine, we combined SLC38A2 knockdown with AOA treatment, which resulted in a significant decrease in COL1A1 protein expression (Figure 4I).

### Alanine deficiency reprograms myofibroblast metabolism.

**Figure 5:**
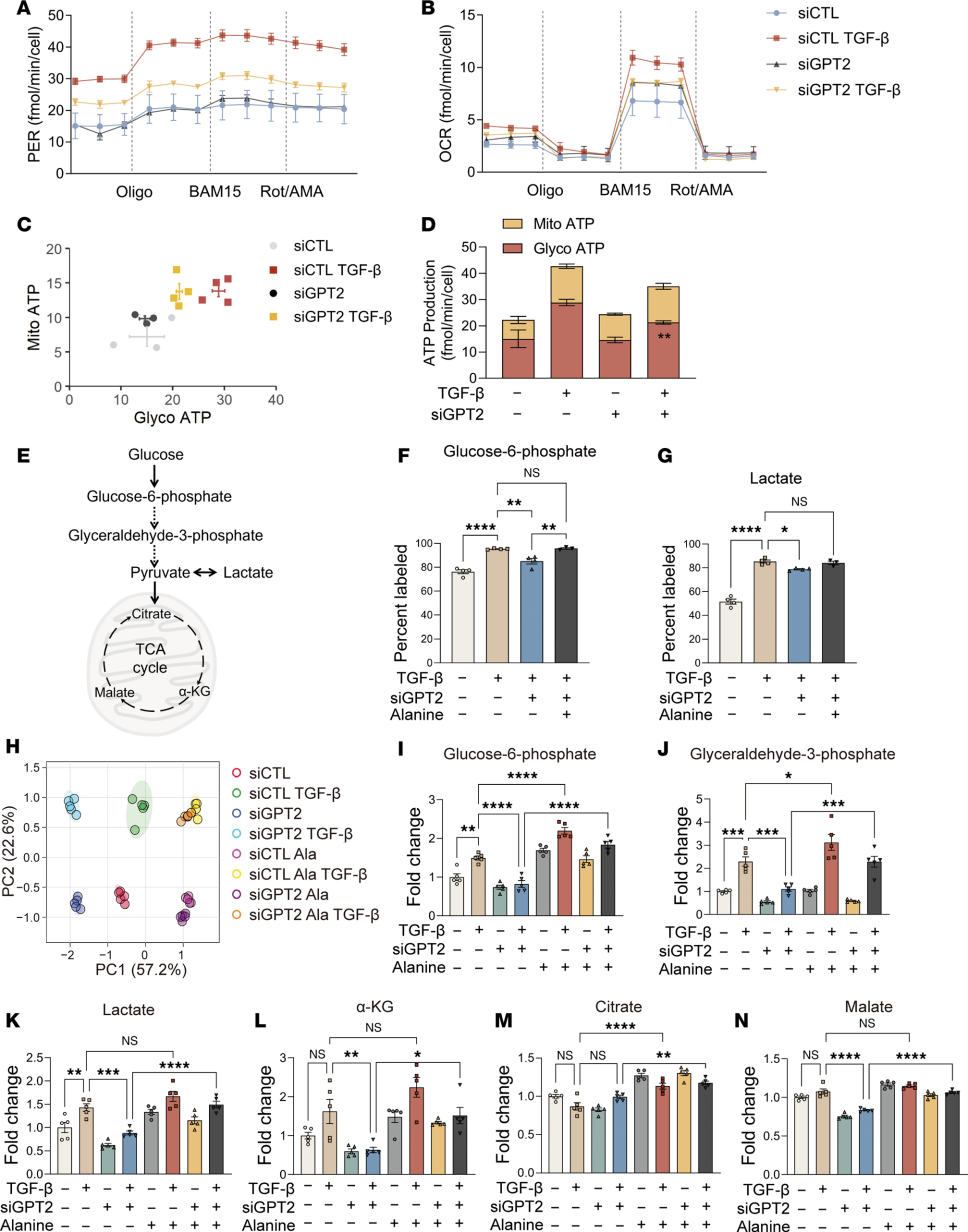
Alanine deficiency reprograms myofibroblast metabolism. (**A** and **B**) Proton export rate (PER) and oxygen consumption rate (OCR) in NHLFs treated with TGF-β for 48 hours, with or without GPT2 knockdown. (**C** and **D**) ATP production from glycolysis and oxidative phosphorylation in NHLFs treated with TGF-β for 48 hours, with or without GPT2 knockdown. (**E**) Schematic overview of glycolysis and the TCA cycle. (**F** and **G**) NHLFs cultured in glucose-free DMEM supplemented with glutamine, pyruvate, and 8 mM [U-¹³C_6_]-glucose and treated with TGF-β for 48 hours. GPT2 knockdown reduced ¹³C incorporation into glucose-6-phosphate (G6P) (**F**) and lactate (**G**), which were restored by alanine supplementation (2 mM). (**H**) PCA showing metabolic shifts after GPT2 knockdown and alanine supplementation in TGF-β–treated NHLFs. (**I–K**) Quantification of intracellular G6P (**I**), glyceraldehyde-3-phosphate (GAP) (**J**), and lactate (**K**) levels in NHLFs after GPT2 knockdown and alanine supplementation. (**L–N**) Quantification of intracellular TCA cycle intermediates: α-KG (**L**), citrate (**M**), and malate (**N**). In all cases, control cells were transfected with nontargeting (control) siRNA. For **D**, 2-way ANOVA; **F**, **G**, and **I–N**, 1-way ANOVA with multiple comparisons. **A–D** show results representative of 3 independent experiments. Data are presented as mean ± SEM. ns, *P* > 0.05; *P < 0.05; **P < 0.01; ***P < 0.001; ****P < 0.0001.

Myofibroblast differentiation in vitro requires increased glycolysis (9, 42). Notably, GPT2 silencing reduced intracellular alanine levels and impaired glucose uptake, accompanied by decreased expression of the key glucose transporter GLUT1, suggesting that alanine availability may influence glycolytic capacity (Supplemental Figure 5, A and B). To further assess the metabolic consequences of alanine deficiency in myofibroblasts globally, we measured proton efflux rate (PER) and oxygen consumption rate (OCR) after GPT2 knockdown and TGF-β stimulation in DMEM, as indicators of flux through glycolysis and oxidative phosphorylation, respectively. As expected, both PER and OCR were elevated after 48 hours of TGF-β stimulation (Figure 5, A and B, and Supplemental Figure 5, C–F). GPT2 knockdown attenuated these effects, reducing both PER and cellular ATP production, primarily through inhibition of glycolysis (Figure 5, C and D). Consistently, pharmacological inhibition of GPT2 with BCA and CS produced similar reductions in PER and glycolytic ATP production rates (Supplemental Figure 5, G–L).

To further elucidate the metabolic alterations in glycolysis after TGF-β treatment in the context of GPT2 knockdown, we conducted metabolic stable-isotope tracing studies (Figure 5E). Lung fibroblasts were cultured in glucose-free DMEM supplemented with [U-^13^C_6_]-glucose (8 mM) and stimulated with TGF-β for 48 hours. GPT2 knockdown reduced TGF-β–induced labeling of glucose-6-phosphate (G6P), while alanine fully restored G6P labeling (Figure 5F). Alanine also increased lactate labeling under GPT2 knockdown conditions, although this increase did not reach statistical significance (Figure 5G). Metabolomic analysis further confirmed that GPT2 knockdown reshaped the intracellular metabolome, as indicated by the distinct clustering of experimental groups in the PCA plot (Figure 5H). Moreover, alanine-supplemented control and GPT2 knockdown overlapped, suggesting metabolic rescue (Figure 5H). Specifically, relative to cells transfected with control siRNA (siCTL), siGPT2 decreased G6P, glyceraldehyde-3-phosphate, and lactate after TGF-β stimulation, all of which were restored by alanine (Figure 5, I–K). Additionally, GPT2 inhibition decreased the abundance of several TCA cycle intermediates (e.g., α-KG, malate, and citrate), while alanine partially rescued their levels (Figure 5, L–N). These findings further support an alanine-dependent role for GPT2 in sustaining glycolytic and TCA cycle metabolite levels, key metabolic features previously associated with myofibroblast differentiation (8).

### Alanine metabolism compensates for glutamine deficiency.

**Figure 6:**
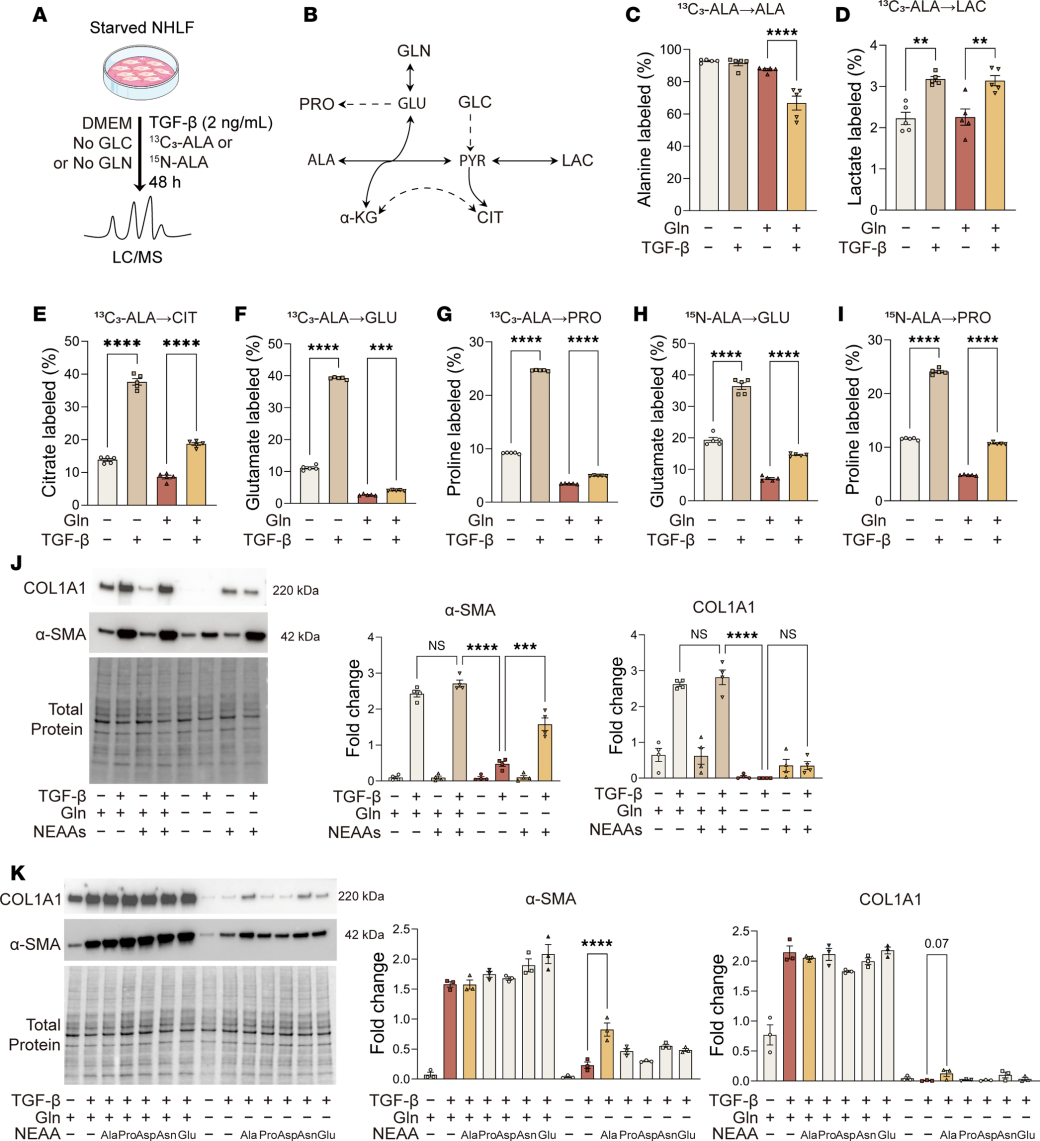
Alanine sustains myofibroblast differentiation by compensating for glutamine deficiency. (**A**) Schematic overview of isotope tracing experiments using [U-^13^C_3_]-alanine or [^15^N]-alanine in NHLFs treated with TGF-β in DMEM with or without 2 mM glutamine (*n* = 5 per group). (**B**) Expected metabolic fate of alanine. (**C**) Percentage of ^13^C_3_-labeled alanine relative to total intracellular alanine derived from extracellular [U-^13^C_3_]-alanine under baseline conditions and after TGF-β stimulation. (**D–G**) Fractional label enrichment of lactate (**D**), citrate (**E**), glutamate (**F**), and proline (**G**) from [U-^13^C_3_]-alanine in NHLFs after TGF-β stimulation for 48 hours. (**H** and **I**) Fractional label enrichment of ^15^N-glutamate (**H**) and ^15^N-proline (**I**) from ^15^N-alanine under glutamine-sufficient and glutamine-deficient conditions after TGF-β treatment for 48 hours. (**J**) Western blot analysis and quantification of α-SMA and COL1A1 expression in fibroblasts cultured in DMEM with or without glutamine after supplementation with a nonessential amino acid (NEAA) cocktail for 48 hours. (**K**) Western blot and quantification of α-SMA and COL1A1 expression after individual supplementation of NEAAs (2 mm) in DMEM with or without glutamine. For Western blot analysis, individual data points represent biological replicates. For **C–J**, 1-way ANOVA with multiple comparisons; for **K**, 1-way ANOVA versus Gln– TGF-β. Data are presented as mean ± SEM. ns, *P* > 0.05; **P < 0.01; ***P < 0.001; ****P < 0.0001.

Myofibroblast differentiation involves both enhanced glycolysis and glutaminolysis (8). Glutamine contributes to proline biosynthesis, a process required for TGF-β–induced extracellular matrix expression (14, 43). GPT2 knockdown decreased glutamate and proline after TGF-β treatment. These reductions were fully restored upon alanine supplementation (Supplemental Figure 6, A and B). Given this observation, we hypothesized that alanine may serve as an alternative carbon and nitrogen source for central metabolic intermediates required for myofibroblast differentiation, particularly during partial or complete glutamine deprivation. To test this, we performed stable isotope tracing using [U-^13^C_3_]- or [^15^N]-alanine in DMEM supplemented with 2 mM glutamine after TGF-β stimulation (Figure 6A). Transamination of [U-^13^C_3_]-alanine produces ^13^C_3_-pyruvate, which can be reduced to ^13^C_3_-lactate by lactate dehydrogenase. Alternatively, ^13^C_3_-pyruvate can be oxidized to ^13^C_2_-acetyl-CoA by pyruvate dehydrogenase or to ^13^C_3_-oxaloacetate by pyruvate carboxylase, yielding ^13^C_2_- and ^13^C_3_-citrate, respectively. Similarly, transamination of [^15^N]-alanine generates [^15^N]-glutamate (Figure 6B). Our data showed that, in the absence of TGF-β, approximately 90% of the total intracellular alanine pool was derived from exogenous [U-^13^C_3_]-alanine (Figure 6C). TGF-β treatment decreased the labeling enrichment of alanine to approximately 60%, suggesting increased endogenous alanine synthesis from unlabeled substrates during myofibroblast differentiation (Figure 6C). Only a very small fraction of labeled lactate (~2%) was detected (Figure 6D), in line with previous findings that show alanine to be a minor contributor to the total intracellular lactate pool (44). Instead, citrate showed substantial ^13^C-label enrichment (~15% and ~40% in the absence or presence of TGF-β, respectively) (Figure 6E and Supplemental Figure 7A), suggesting that a greater fraction of the total citrate pool, when compared with the total lactate pool, is derived from alanine. Interestingly, under glutamine-deficient conditions, TGF-β stimulation increased the labeling of glutamate (~10% to ~40%), proline (~10% to ~25%), glutamine (~10% to ~25%), malate (~5% to ~10%), and aspartate (~10% to ~20%), indicating alanine can be an important alternative carbon source for these metabolites (Figure 6, F and G, and Supplemental Figure 7, B–D).

In the [^15^N]-alanine experiment, approximately 10% of the total glutamate and proline pools were ^15^N-labeled under glutamine-sufficient conditions after TGF-β stimulation, and ^15^N labeling enrichment increased substantially during glutamine deprivation (~35% for glutamine and glutamate, and ~25% for proline) (Figure 6, H and I, and Supplemental Figure 7E), demonstrating reverse GPT2 flux allowing transamination of α-KG for glutamate and glutamine production.

Since the enhanced synthesis of proline from glutamine facilitates TGF-β–induced matrix protein production, we next investigated whether alanine could compensate for glutamine deficiency to maintain α-SMA and COL1A1 expression. We first applied a commercial NEAA cocktail, which was sufficient to partly rescue AOA-induced inhibition of TGF-β–stimulated α-SMA and COL1A1 protein expression (Supplemental Table 2 and Supplemental Figure 7F). Under glutamine-deficient conditions, α-SMA and COL1A1 protein expression was attenuated after TGF-β stimulation (Figure 6J). The NEAA cocktail partially restored α-SMA protein expression, while only modestly increasing COL1A1 production (Figure 6J). Next, we supplemented each NEAA individually. Only alanine partially restored the α-SMA protein expression, with minimal change in the COL1A1 protein (Figure 6K). These data identify a unique and critical role for alanine as a carbon and nitrogen source for central metabolic intermediates, which may support myofibroblast differentiation under glutamine-deprived conditions.

### SLC38A2 and GPT2 cooperate to drive myofibroblast function and fibrogenic protein expression.

**Figure 7:**
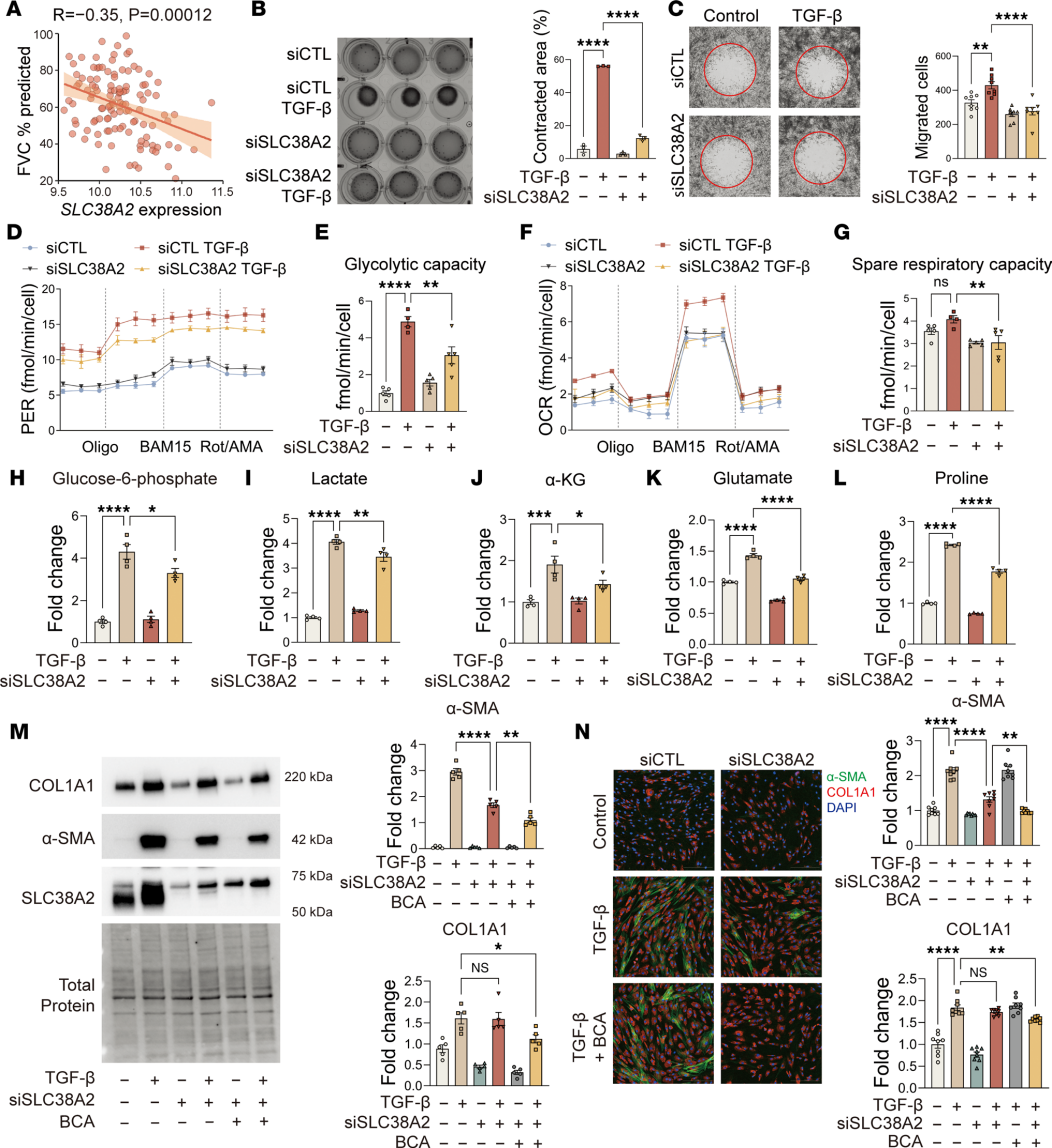
SLC38A2 and GPT2 cooperatively promote TGF-β–induced fibrogenic responses. (**A**) Pearson’s correlation between *SLC38A2* mRNA levels and forced vital capacity (FVC) predicted for each patient, from clinical data of GSE32537. (**B**) Representative images of collagen gel contraction after 24 hours of TGF-β treatment in FBM with or without SLC38A2 knockdown (left). Quantification of gel contraction expressed as the percentage reduction in gel area relative to baseline (0 h) (right). (**C**) Representative images of control and SLC38A2-knockdown NHLFs migrating into the cell-free zone 24 hours after TGF-β treatment in FBM (left). Quantification of migrated cells within the defined area based on DAPI staining (right). (**D**–**G**) PER (**D**), glycolytic capacity (**E**), OCR (**F**), and spare respiratory capacity (**G**) in NHLFs after 48 hours of TGF-β treatment in FBM, with or without SLC38A2 knockdown. (**H**–**L**) Quantification of intracellular G6P (**H**), lactate (**I**), α-KG (**J**), glutamate (**K**), and proline (**L**) levels in NHLFs after SLC38A2 knockdown and TGF-β treatment. (**M**) Western blot analysis and quantification of α-SMA and COL1A1 expression in NHLFs treated with TGF-β for 48 hours, with or without SLC38A2 knockdown and β-chloro-L-alanine (BCA, 100 μM) treatment. (**N**) Immunofluorescence staining and quantification of α-SMA and COL1A1 expression in IPF patient-derived fibroblasts after SLC38A2 knockdown and BCA (100 μM) treatment under TGF-β stimulation (*n* = 8 per group). For Western blot analysis, individual data points represent biological replicates. **B**–**G** and **N** show results representative of 3 independent experiments. For **A**, linear regression and Pearson’s correlation. For **B**, **C**, **E**, **G**, and **H**–**N**, 1-way ANOVA. Data are presented as mean ± SEM. ns, *P* > 0.05; *P < 0.05; **P < 0.01; ****P < 0.0001.

Based on the cooperative regulation of alanine metabolism by GPT2 and SLC38A2 and their essential roles in promoting myofibroblast differentiation, we assessed their clinical relevance in IPF, examined key myofibroblast functional and metabolic phenotypes, and explored their potential as therapeutic targets. In a publicly available whole-lung RNA-Seq dataset (GSE32537), we examined the relationship between *SLC38A2* and *GPT2* expression and lung function (45). Increased SLC38A2 expression, but not GPT2 expression, correlated with more impaired lung function as measured by forced vital capacity (Figure 7A and Supplemental Figure 8A).

Myofibroblast differentiation is characterized by enhanced contractile capacity, with α-SMA playing a key role in maximal force generation (7, 46). Since SLC38A2 knockdown reduces alanine uptake and limits glutamine for GPT2-dependent alanine synthesis, thereby impairing α-SMA expression (Figure 4G), we hypothesized that SLC38A2 inhibition may attenuate myofibroblast contractile function. To test this hypothesis, we evaluated SLC38A2 function in TGF-β–treated fibroblasts cultured in FBM using a collagen gel contraction assay, a functional readout of myofibroblast-mediated matrix contraction. SLC38A2 knockdown significantly reduced TGF-β–induced gel contraction to near baseline levels (Figure 7B). We also measured fibroblast migratory capacity under the same conditions and found that siSLC38A2 markedly decreased migration to levels comparable to unstimulated controls (Figure 7C). These functional impairments were accompanied by reductions in both PER and OCR (Figure 7, D–G, and Supplemental Figure 8, B and C), along with decreased levels of G6P, lactate, α-KG, glutamate, and proline (Figure 7, H–L), indicating that SLC38A2 links amino acid transport to bioenergetic and anabolic programs required for myofibroblast differentiation.

Given that SLC38A2 knockdown only partially reduced GPT2 protein expression, it failed to fully block alanine synthesis (Figure 4G). We further explored the feasibility of targeting both alanine synthesis and uptake simultaneously to ameliorate pulmonary fibrosis. We attempted pharmacological inhibition of both targets to facilitate future translation to animal models and to avoid transfection reagents. GPT2 inhibition was achieved using BCA, which was validated by metabolomic profiling and recapitulated the effects observed with GPT2 knockdown (Figure 3). For SLC38A2 inhibition, the neutral amino acid transporter inhibitor MeAIB has been reported; however, its specificity for SLC38A2 and its efficacy in blocking alanine uptake under nutrient-replete conditions remain uncertain (47, 48). We performed short-term labeled alanine and glutamine uptake assays and found that MeAIB (up to 50 mM) did not inhibit alanine or glutamine import (Supplemental Figure 8D). Consistently, MeAIB treatment did not reproduce the phenotypic effects observed with SLC38A2 knockdown, as confirmed by Western blot (Supplemental Figure 8E), immunofluorescence (Supplemental Figure 8F), and functional assays (Supplemental Figure 8, G and H) in FBM. Therefore, we relied on siRNA-mediated knockdown combined with BCA. Our results show that SLC38A2 knockdown in NHLFs suppressed TGF-β–induced α-SMA protein expression in FBM, and that this suppression was potentiated in the presence of BCA-mediated GPT inhibition (Figure 7M). Combined SLC38A2 knockdown and BCA treatment also significantly reduced COL1A1 protein expression (Figure 7M). To validate these findings in a disease-relevant context, we performed immunofluorescence staining in fibroblasts derived from patients with IPF in FBM. The data showed a similar inhibitory effect on α-SMA and COL1A1 protein expression of SLC38A2 knockdown combined with BCA treatment (Figure 7N). These findings collectively demonstrate that dual inhibition of alanine synthesis and transport markedly attenuates TGF-β–induced fibrogenic protein expression.

### Combined inhibition of alanine synthesis and uptake attenuates pulmonary fibrosis.

**Figure 8:**
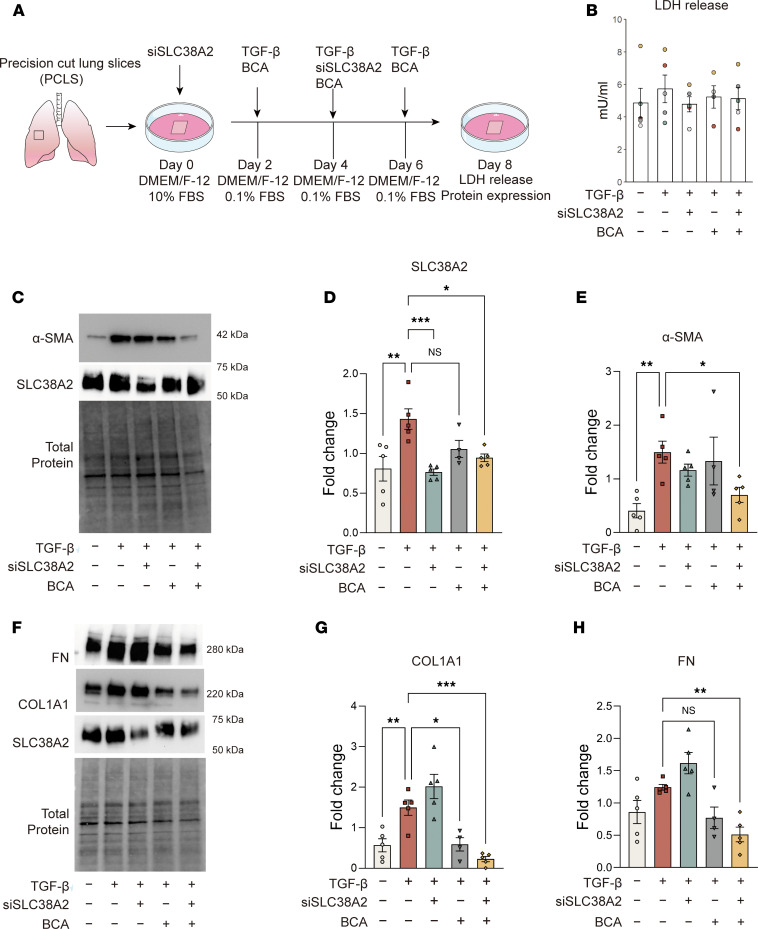
Dual inhibition of alanine uptake and synthesis alleviates pulmonary fibrosis. (**A**) Schematic overview of experiments using commercially available precision-cut lung slices (PCLS) derived from healthy donors to investigate the role of GPT inhibition and SLC38A2 knockdown in pulmonary fibrosis. In all cases, control PCLS were transfected with nontargeting (control) siRNA. (**B**) Supernatants collected from day 6 to day 8 were subjected to LDH assay. Each color represents an independent biological replicate. (**C**–**E**) Western blot analysis (**C**) and quantification of SLC38A2 (**D**) and α-SMA (**E**) expression in PCLS after TGF-β stimulation with or without SLC38A2 knockdown and BCA (250 μM) treatment. (**F**–**H**) Western blot analysis (**F**) and quantification of COL1A1 (**G**) and fibronectin (FN) (**H**) expression in PCLS after combined SLC38A2 knockdown and BCA (250 μM) treatment. For Western blot analysis, individual data points represent biological replicates. For **D**, **E**, **G**, and **H**, 1-way ANOVA versus TGF-β. Data are presented as mean ± SEM. ns, *P* > 0.05; *P < 0.05; **P < 0.01; ***P < 0.001.

To extend our findings to a more physiologically relevant model, we employed precision-cut lung slices (PCLS), which preserve the native lung architecture and multicellular microenvironment, including both airway and alveolar compartments, making them highly valuable for studying fibrotic pathogenesis, pharmacological responses, and therapeutic discovery in pulmonary fibrosis (49–51). As illustrated in Figure 8A, commercially available PCLS from nondiseased human donors were cultured, transfected with SLC38A2 siRNA, and treated with TGF-β and BCA in DMEM/F-12 medium, which contains both alanine and glutamine (Supplemental Table 3).

Previous studies have demonstrated that PCLS can be maintained for up to 14 days while preserving tissue integrity and metabolic activity (52, 53). After treatment with inhibitors or siRNA, our data showed that PCLS remained responsive to TGF-β. To assess tissue viability under our experimental conditions, we measured LDH release in PCLS culture supernatants as an indicator of cellular injury. LDH release was comparable across all experimental groups, indicating preserved tissue viability throughout the culture period (Figure 8B). Consistent with our in vitro findings, SLC38A2 protein expression was upregulated after TGF-β treatment in PCLS, which was suppressed by SLC38A2 knockdown (Figure 8, C and D). TGF-β also induced key fibrogenic proteins in PCLS, including α-SMA, COL1A1, and fibronectin (Figure 8, E, G, and H). In combination with BCA, SLC38A2 knockdown significantly decreased α-SMA protein levels (Figure 8, C and E). Notably, BCA alone decreased COL1A1 upregulation after TGF-β treatment; the combined intervention potentiated the decrease in both COL1A1 and fibronectin protein expression (Figure 8, F–H). Collectively, these results show that combined inhibition of alanine synthesis and uptake effectively ameliorates pulmonary fibrosis after TGF-β treatment ex vivo.

## Discussion

This study identifies a role for alanine metabolism in myofibroblast differentiation, revealing dynamic coordination between intracellular synthesis via GPT2 and transporter-mediated uptake via SLC38A2. Our findings demonstrate that this alanine metabolic axis sustains profibrotic activation and represents a context-dependent metabolic vulnerability in pulmonary fibrosis.

Myofibroblast differentiation is characterized by increased expression of α-SMA and collagen, accompanied by functional reprogramming, including enhanced contractility and migratory capacity. This process relies on key nutrients, particularly glucose and glutamine, as inhibition of glycolysis or glutamine deprivation effectively suppress myofibroblast differentiation (19). Multiple amino acids, including proline, serine, and glycine, contribute to collagen biosynthesis and matrix production in pulmonary fibrosis (10, 14, 54). In this study, we identified alanine as a critical NEAA in myofibroblast differentiation. To dissect the role of alanine metabolism, we intentionally employed two distinct nutrient contexts. DMEM, which lacks alanine and several NEAAs, enabled interrogation of GPT2-dependent de novo alanine synthesis and the metabolic consequences of alanine deficiency. In contrast, FBM provides an alanine-replete environment that more closely reflects physiological conditions, allowing evaluation of the combined contributions of alanine synthesis and uptake. The differential responses observed across these widely used media demonstrate that nutrient context shapes metabolic dependency. This nutrient-context dependency was further illustrated by pharmacological transaminase inhibition: although the transaminase inhibitor AOA had been previously shown to suppress α-SMA protein expression in myofibroblasts (13), we have found that its inhibitory effect requires alanine deficiency as α-SMA upregulation was substantially attenuated under alanine-replete conditions (i.e., when cells were cultured in FBM or alanine-supplemented DMEM). These findings expose an important limitation of DMEM as a culture medium for metabolic studies of myofibroblast differentiation. By lacking alanine and other NEAAs present in vivo, DMEM creates artificial metabolic dependencies that may lead to erroneous conclusions about the essentiality of specific metabolic pathways. Researchers studying myofibroblast metabolism should carefully consider whether standard culture media adequately recapitulate the nutrient environment in vivo before drawing conclusions about metabolic requirements.

Alanine homeostasis is maintained through coordinated intracellular synthesis and transporter-mediated uptake. Under alanine-sufficient conditions, intracellular alanine is sustained by SLC38A2-dependent uptake, which is highly expressed and further upregulated by profibrotic stimulation such as TGF-β. When extracellular alanine is limited, TGF-β–induced GPT2 expression becomes critical for sustaining intracellular alanine pools. Under these conditions, enhanced glycolysis and glutamine utilization provide the carbon (pyruvate) and nitrogen (glutamate) required for GPT2-mediated alanine synthesis. Glutamine tracing further confirmed incorporation of ^13^C from glutamine into alanine, indicating that glutamine-derived carbon can enter the TCA cycle and contribute to pyruvate pools, thereby supporting alanine production. We demonstrate that the glutamine/glutamate/α-KG axis contributes to the TGFβ-induced upregulation of GPT2. These findings are consistent with prior evidence that inhibition of SLC1A5 with V-9302 reduces glutamine import and extracellular matrix deposition in experimental lung fibrosis (29). We found that knockdown of SLC38A2, in addition to SLC1A5, also reduced GPT2 levels, indicating that multiple glutamine transporters cooperate to maintain intracellular glutamine availability. Furthermore, V-9302 has been reported to inhibit SLC38A2-mediated transport (55, 56), suggesting that its antifibrotic effects may not be exclusively mediated through SLC1A5. Collectively, these observations support a model in which glutamine transport acts upstream to sustain the alanine/GPT2 metabolic axis under profibrotic conditions. Importantly, a profibrotic cocktail similarly induced GPT2 and SLC38A2 and increased intracellular alanine, indicating that the GPT2/SLC38A2/alanine axis represents a conserved metabolic response to diverse fibrotic stimuli. Given that matrix stiffness is a well-established regulator of fibroblast activation and metabolic reprogramming (57, 58), biomechanical cues may further modulate alanine synthesis and transporter expression, thereby influencing metabolic flexibility in fibrotic tissues.

Previous studies have highlighted the contribution of alanine metabolism in tumor, neuronal, and immune cell function (36, 44, 59, 60). Our metabolomic analyses indicate that intracellular alanine deficiency impaired glucose uptake and diminished glycolytic flux accompanied by reduced α-SMA and COL1A1 expression, leading to compensatory upregulation of SLC38A2. α-SMA and COL1A1 are classical myofibroblast markers, and fibroblast activation also includes extracellular matrix remodeling and profibrotic signaling programs. Under alanine-restricted conditions, GPT2 knockdown further decreased *FN1* and *CTGF* at the transcriptional level, in addition to *ACTA2* and *COL1A1*, supporting a role for alanine metabolism in transcriptional reprogramming of fibroblast activation. In parallel, suppression of SLC38A2 limits both alanine uptake and glutamine availability, thereby restricting glutamate-proline metabolism required for collagen synthesis. Together, disruption of the GPT2/SLC38A2 axis reduced intracellular alanine levels, impaired central carbon metabolism, and ultimately attenuated myofibroblast differentiation.

Glutamine metabolism has been explored as a therapeutic target in cancer and pulmonary fibrosis (12, 13, 61, 62), but compensatory pathways often undermine single-target interventions. Indeed, our data demonstrate that, under glutamine-deficient conditions, alanine compensates for glutamine as a nitrogen and carbon source to sustain α-SMA protein expression during TGF-β–induced myofibroblast differentiation. Although this study focuses on fibroblasts, single-cell transcriptomic analyses indicate that SLC38A2 is expressed in specific mesenchymal subpopulations within the fibrotic lung, implying that alanine transport may contribute more broadly to stromal metabolic reprogramming. Moreover, given the established roles of amino acid metabolism in immune and epithelial cell function, alanine-derived carbon may support central metabolic pathways, including the TCA cycle, to meet the biosynthetic and energetic demands of diverse cell types during tissue remodeling.

Publicly available transcriptomic data demonstrate that SLC38A2 is highly expressed in fibroblasts and correlates with disease severity in patients with IPF, supporting its potential relevance in fibrotic lung disease. In contrast, GPT2 expression does not correlate with forced vital capacity in IPF cohorts. This lack of association may reflect the nutrient-sufficient in vivo environment, in which extracellular alanine availability could compensate for inhibition of de novo synthesis alone. Consistent with this context-dependent model, our data indicate that targeting GPT2 alone is insufficient under alanine-replete conditions, supporting a strategy that simultaneously disrupts alanine synthesis and uptake. In contrast to the pronounced effects of GPT2 knockdown under alanine-deficient conditions, SLC38A2 knockdown in alanine-replete settings significantly attenuated TGF-β–induced glycolytic intermediates, including G6P and lactate, but to a lesser extent. Consistently, SLC38A2 depletion reduced TGF-β–induced COL1A1 expression only modestly, potentially reflecting compensatory alanine or glutamine uptake through alternative transporters. Importantly, SLC38A2 inhibition markedly suppressed contractile and migratory capacities associated with myofibroblast activation. These findings suggest that nutrient transport represents a functional vulnerability in profibrotic remodeling.

PCLS have emerged as a physiologically relevant ex vivo model that preserves lung tissue architecture and cellular interactions while allowing genetic manipulation or pharmacological intervention. In our study, we used human PCLS to demonstrate that combined inhibition of SLC38A2-mediated alanine uptake and GPT2-mediated synthesis effectively reduces TGF-β–induced expression of fibrotic markers, including α-SMA, COL1A1, and fibronectin. SLC38A2 knockdown alone exhibited only a modest efficacy with respect to suppressing markers of myofibroblast differentiation (i.e., α-SMA and COL1A1), possibly due to a lower knockdown efficiency in PCLS as compared with a monolayer cell culture (i.e., likely a result of less efficient siRNA delivery). This limitation notwithstanding, PCLS remain a valuable translational model in pulmonary research, as they better recapitulate the native cellular microenvironment. Our experiments using human PCLS provide key insights into the in vivo relevance of targeting alanine metabolism in IPF.

Several limitations of the current study should be acknowledged and warrant further investigation. First, conditional SLC38A2 or GPT2 knockout animal models will be necessary to validate the role of alanine metabolism in fibrosis in vivo and to further evaluate the therapeutic potential of this pathway. Second, current SLC38A2 inhibitors have limited specificity, selectivity, and efficacy (55, 63), highlighting the need for improved inhibitors with enhanced selectivity and cell type–specific targeting of SLC38A2 in myofibroblasts. Third, whether combined inhibition of alanine synthesis and uptake exhibits synergistic antifibrotic effects in vivo remains to be determined.

Our findings propose a broader concept in which disrupting the metabolic flexibility required for fibrogenic responses may represent an effective therapeutic strategy for pulmonary fibrosis. The ability of activated fibroblasts to draw on multiple nutrient sources — switching between de novo synthesis and transporter-mediated uptake to maintain critical intracellular metabolite pools — exemplifies the adaptive capacity that has limited the efficacy of single-target metabolic interventions in fibrosis and cancer alike. Overcoming this adaptability will likely require strategies that simultaneously target complementary nodes within a metabolic network, rather than individual enzymes or transporters in isolation. Further investigations into how nutrient availability, tissue stiffness, and the broader fibrotic microenvironment shape cellular metabolic adaptability will be essential for identifying the combinations of targets most likely to overcome compensatory rewiring. As our understanding of metabolic reprogramming in fibrosis matures, therapeutic strategies that account for — and actively exploit — the context-dependence of metabolic dependencies may offer a more durable path to antifibrotic efficacy.

## Methods

### Sex as a biological variable

This study utilized primary fibroblasts and PCLS derived from both male and female donors.

### Fibroblast cell culture

Primary NHLFs were obtained from Lonza, cryopreserved at passage 3, and used between passages 3 and 8. Cells were cultured in FGM-2 medium, consisting of FBM basal medium (Lonza) supplemented with FBM SingleQuots Supplements and Growth Factors (Lonza), and subjected to starvation in serum-free DMEM or FBM without supplement for 24 hours unless otherwise specified. Cells were then treated with recombinant human TGF-β1 (2 ng/mL) for 48 hours to induce myofibroblast differentiation in vitro.

Lung fibroblasts from patients with IPF were derived from lung transplantation surgeries. The study protocol and sample collection were approved by the Mass General Brigham IRB (2013P002332, 2016P001890, 2019P003592, 2020P002765). Lung tissues were minced and digested with Liberase and DNase I (Sigma-Aldrich), followed by filtration using a sterile 70 μm filter. The filtrates were centrifuged at 300*g*; washed once with RPMI medium (Lonza); and plated in DMEM supplemented with 10% (v/v) FBS, penicillin, and streptomycin. After 2 passages, the medium was replaced with FGM-2 medium. Cells were cryopreserved in liquid nitrogen at passage 3 and used for experiments at passage 4.

### PCLS

PCLS were obtained from Anabios and cultured in DMEM/F-12 medium containing 10% (v/v) FBS for 48 hours. siRNA transfections and treatment with TGF-β alone or in combination with inhibitors were performed, with fresh media and treatments replenished every 48 hours. On day 8, slices were collected, lysed in RIPA lysis buffer with protease inhibitors, and homogenized using bead beating for protein extraction. Western blotting was performed as described above. Experiments included 5 biological replicates.

### Chemical reagents and preparation

Reagents, antibodies, and other key resources are listed in Supplemental Table 4. Recombinant human TGF-β1 was dissolved in 10 mM citric acid (pH 3.0), filtered, and diluted to 10 μg/mL in PBS with 0.1% BSA prior to aliquoting and storing at –80 °C. β-Chloro-l-alanine and L-cycloserine were dissolved in water, and aminooxyacetic acid was dissolved in DMSO. Unless otherwise specified, all reagents were used at the indicated concentrations based on previous studies.

### Isotope labeling experiments

For alanine uptake experiments, cells treated with TGF-β for 24 hours were switched to fresh DMEM supplemented with 2 mM ^13^C_3_-l-alanine. For glutamine and alanine co-uptake experiments, cells subjected to SLC38A2 knockdown for 48 hours were switched to fresh glutamine-free DMEM containing 2 mM ^13^C_3_-l-alanine and 2 mM [α-^15^N_1_]-glutamine. Similarly, for glutamine uptake experiments, cells pretreated with 2 mM unlabeled alanine in DMEM for 24 hours were switched to fresh glutamine-free DMEM containing 2 mM ^13^C_5_-glutamine and 2 mM unlabeled alanine. After 1 hour of incubation, cells were washed 3 times with LC/MS-grade water (Thermo Fisher Scientific), and lysates were prepared for metabolomic analysis as described below.

To study alanine synthesis, cells starved in DMEM for 24 hours were switched to glucose-free DMEM supplemented with ^13^C_6_-glucose, glutamine-free DMEM supplemented with ^13^C_5_-glutamine, or glutamine-free DMEM supplemented with [α-^15^N_1_]-glutamine. After 48 hours, cells were washed with LC/MS-grade water twice, and lysates were prepared for metabolomic analysis as described below.

Alternatively, to study the metabolic fate of alanine, cells starved in DMEM for 24 hours were switched to glutamine-free DMEM supplemented with 2 mM ^13^C_3_-l-alanine or ^15^N-l-alanine, with or without 2 mM glutamine, and treated with TGF-β for 48 hours. Cells were then washed twice with LC/MS-grade water, and lysates were prepared for metabolomic analysis as described below.

### mRNA extraction and quantitative RT-PCR

Total RNA from NHLFs was isolated using the RNeasy Mini kit (QIAGEN) according to the manufacturer’s instructions. cDNA was synthesized using the PrimeScript RT reagent kit (Takara). Quantitative real-time PCR was performed using PowerUp SYBR Green Master Mix (Thermo Fisher Scientific) on a QuantStudio 6 (QS6) Real-Time PCR system (Thermo Fisher Scientific). Both HPRT and GAPDH were used as internal reference genes for normalization. Primer sequences are provided in Supplemental Table 5.

### Western blotting

Cells were lysed on ice in buffer containing Tris 10 mM (pH 7.4), NaCl 150 mM, EDTA 1 mM, EGTA 1 mM, Triton X-100 1% v/v, NP-40 0.5% v/v, and 1x Halt Protease and Phosphatase Inhibitor Cocktail (Thermo Fisher Scientific). Protein concentrations were measured using the bicinchoninic acid (BCA) assay. Lysates were standardized to 10–20 μg of protein (30 μg of protein was used for the p-Smad3 immunoblot), separated by SDS-PAGE on stain-free Tris-glycine gels (Bio-Rad), cross-linked by 45 seconds of UV illumination, and imaged using the ChemiDoc system (Bio-Rad). Proteins were transferred to PVDF membranes using the Trans-Blot Turbo transfer system (Bio-Rad). Stain-free membrane images were used to assess total protein loading.

Membranes were blocked in 5% (v/v) milk (Bio-Rad), incubated with primary antibodies overnight at 4 °C, followed by incubation with secondary antibodies at room temperature for 1 hour. Bands were visualized using WesternBright ECL reagent (Advansta). Band intensities were quantified using Image Lab software (Bio-Rad) and normalized to total protein signals. Quantitative values are presented as target-to-total protein ratios, and statistical analyses were performed using data from multiple independent biological replicates.

### RNA interference

GPT1, GPT2, SLC1A5, or SLC38A2 siRNA (Dharmacon) was reverse-transfected into 4 × 10^5^ lung fibroblasts per well in 6-well plates using 20 pmol siRNA and 7.5 μL Lipofectamine RNAiMAX transfection reagent (Thermo Fisher Scientific). Nontargeting siRNA was used as a transfection control. After 24 hours of transfection, cells were serum-starved for an additional 24 hours, followed by TGF-β treatment (2 ng/mL).

### Immunofluorescence staining

Cells were seeded at 8,750 cells/well in 96-well black-wall, clear-bottom plates. After 24 hours, cells were serum-starved for 24 hours and treated with TGF-β and inhibitors for 48 hours. For combined SLC38A2 knockdown and GPT inhibitor treatment, cells were transfected for 24 hours, seeded, and starved for 24 hours. After treatments, cells were washed with PBS, fixed in 4% (v/v) paraformaldehyde for 15 minutes, and permeabilized with 0.5% (v/v) Triton X-100 for an additional 15 minutes. Blocking was performed with 3% (v/v) BSA (Sigma-Aldrich) for 30 minutes at room temperature. Cells were then incubated with primary antibodies against α-SMA (1:1,500) and COL1A1 (1:1,000) at room temperature for 2 hours. After 3 washes with PBS, cells were incubated with fluorescently labeled secondary antibodies (goat anti-mouse Alexa Fluor 488, 1:1,000, and goat anti-rabbit Alexa Fluor 647, 1:1,000) for an additional 45 minutes at room temperature. After 3 washes with PBS, nuclei were stained with DAPI (2 μg/mL) for 10 minutes, followed by 3 PBS washes. Immunofluorescence images were acquired using a Cytation 5 Cell Imaging Multi-Mode Reader (Agilent BioTek Instruments). Cells were imaged using a 20× objective for representative images and a 4× objective for quantitative analysis, with filter channels for DAPI, Alexa Fluor 488, and Alexa Fluor 647. Exposure time and gain settings were kept consistent across all samples. Image acquisition and analysis including cell counting and total fluorescence intensity per cell were performed automatically using Gen5 software.

### Seahorse assay

Lung fibroblasts transfected with GPT2 or SLC38A2 siRNA for 24 hours were seeded at 20,000 cells/well in 24-well Seahorse plates. After 24 hours of serum starvation and subsequent 48 hours of TGF-β treatment, the medium was replaced with Seahorse XF DMEM (Agilent, 103335-100) supplemented with 10 mM glucose, 2 mM glutamine, and 1 mM pyruvate. Basal OCR and PER were measured using an XFe24 analyzer (Agilent), with sequential injections of oligomycin (1 μM), BAM15 (2.5 μM), and rotenone/antimycin A (0.5 μM each). After the assay, cells were stained with Nuclear Green LCS1 (10 μM; Abcam) for 30 minutes and counted using a Cytation 5 imager for normalization.

### Metabolomics

#### Sample preparation.

Extracellular metabolites were extracted by mixing conditioned media with precooled LC/MS-grade methanol (Honeywell) at a 1:4 (v/v) media/methanol ratio on ice. For analysis of intracellular metabolites, cells were rapidly washed twice with HPLC-grade water and snap-frozen by placing the plates on liquid nitrogen. Plates were stored at −80 °C until metabolite extraction. Metabolites were extracted with 500 μL of extraction buffer consisting of 50:30:20 (v/v/v) LC/MS-grade methanol/acetonitrile/water containing 1 μM D_8_-valine and ^13^C_5_-glutamine as internal standards (internal standards were not added for tracing experiments) at −80 °C. Samples were centrifuged at 17,000*g* for 10 minutes at 4°C. Supernatants were dried using a SpeedVac concentrator (Thermo Savant) at 42°C, reconstituted in 50 μL of HPLC-grade water, centrifuged at 17,000*g* for 10 minutes at 4°C, and transferred (35 μL) to LC/MS vials.

#### LC/MS data acquisition and analysis.

LC/MS analyses were performed using a Vanquish UHPLC system connected to a Q Exactive Orbitrap mass spectrometer with a HESI-II source (Thermo Fisher Scientific). Samples were stored in the autosampler at 5°C prior to injection. The following chromatographic parameters were used: solvent A, 20 mM ammonium carbonate supplemented with 5 μM medronic acid, pH corrected to 9.2 with ammonium hydroxide; solvent B, acetonitrile; injection volume, 2 μL; oven temperature, 25°C; flow rate, 0.1 mL/min. Compounds were separated using a ZIC-pHILIC stationary phase (150 mm × 2.1 mm × 3.5 mm; Merck) with a guard column and a linear gradient of solvent B as follows: 0 minutes, 80%; 20 minutes, 20%; 20.5 minutes, 8%; 24 minutes, 8%; 24.5 minutes, 80%; 35 minutes, 80%. MS scan parameters were as follows: scan type, full MS; scan range, 60–900 Th; fragmentation, none; resolution, 70,000; microscans, 1; lock masses, off; AGC target, 1 × 10^6^; maximum injection time, 80 ms. The MS was operated in polarity switching mode. HESI-II source parameters were as follows: sheath gas flow rate, 40, auxiliary gas flow rate, 15; sweep gas flow rate, 1; spray voltage, 1 kV; capillary temperature, 320°C; S-lens RF level, 50; auxiliary gas heater temperature, 350°C. Peak identification was performed using TraceFinder software (Thermo Fisher Scientific), using an in-house library of metabolites with known retention times, as assessed and optimized previously with authentic standards using the same method parameters. To correct for total signal variation across samples, AUCs of integrated peaks were normalized to the total sum of all AUCs in the same sample. Volcano plots, heatmaps, and Kyoto Encyclopedia of Genes and Genomes pathway enrichment plots were generated using R (version 4.4.3) with relevant packages.

### Gel contraction assay

NHLFs were transfected with either control siRNA or SLC38A2 siRNA for 24 hours, followed by resuspension in FBM and neutralized collagen solution (TeloCol-3, Advanced Biomatrix) at a 1:2 ratio to yield a final concentration of 100,000 cells/mL and 1.8 mg/mL collagen. The mixture (0.5 mL) was added to 24-well plates and allowed to harden for 10 minutes at 37 °C, after which 1 mL of FBM was added. Cells were serum-starved overnight prior to gel release. Baseline (0 h) images were acquired using a ChemiDoc imager (Bio-Rad), followed by treatment with TGF-β (2 ng/mL). After 24 hours, images were acquired, and gel areas were measured using Image Lab software (Bio-Rad). Data were expressed as the percentage of gel contraction relative to the initial area at 0 h using the formula: [(Area at 0 h − Area at 24 h) / Area at 0 h] × 100.

### Cell migration assay

NHLFs were transfected with either control siRNA or SLC38A2 siRNA for 24 hours, then seeded at a density of 1 × 10^5^ cells/mL (100 μL per well) into 96-well migration plates equipped with stoppers (Platypus Technologies). After overnight serum starvation, the stoppers were removed, baseline (0 h) images were acquired using the white light channel of the Cytation 5 Cell Imaging Multi-Mode Reader, and the initial cell-free migration zone was defined. Cells were then treated with TGF-β (5 ng/mL) and allowed to migrate for 24 hours. After migration, cells were fixed and permeabilized with 4% (v/v) paraformaldehyde containing 0.5% (v/v) Triton X-100 for 15 minutes, followed by staining with DAPI. Fluorescence images were acquired using the DAPI channel of the Cytation 5 system. The number of cells that migrated into the defined zone was quantified using ImageJ (NIH). For representative visualization, selected wells were stained with 0.1% (v/v) crystal violet for 15 minutes and imaged using the white light channel of the Cytation 5 system.

### Statistics

Unless otherwise stated, data analysis, visualization, and statistical comparisons were performed using GraphPad Prism 10. Quantification of Western blots was based on at least 3 biological replicates. Seahorse and other functional assays were independently performed in a minimum of 3 biological replicates, and representative data from a single experiment are shown. Data are presented as mean ± SEM. Normality was assessed using the Shapiro-Wilk test. Two-group comparisons were performed using 2-tailed unpaired *t* tests, Welch’s *t* tests, or Mann-Whitney *U* tests, as appropriate. For comparisons among multiple groups, 1-way ANOVA followed by Tukey’s or Dunnett post hoc test was used. For experiments involving 2 independent variables, 2-way ANOVA with Tukey’s post hoc test was applied. Correlations were assessed using linear regression and Pearson’s correlation. *P* values less than 0.05 were considered statistically significant.

### Study approval

The investigation involving clinical samples was approved by the Mass General Brigham IRB (2013P002332, 2016P001890, 2019P003592, 2020P002765), and lung tissues from patients with IPF for primary fibroblast culture were obtained from lung transplantation surgeries with prior written informed consent from patients or their families.

### Data availability

All data associated with this study are available in the main text, the supplemental materials, and the Supporting Data Values file. The graphical abstract was created with BioRender.com. Any additional information required to reanalyze the data reported in this paper is available from the corresponding author upon request.

## Author contributions

FL and WMO contributed to conceptualization. FL, NV, and WMO contributed to methodology. FL, NV, DRZ, and WMO conducted the investigation. FL, NV, and WMO contributed to visualization. WMO acquired funding, was responsible for project administration, and supervised the study. DRZ and WMO provided resources. FL and NV wrote the original draft of the manuscript. FL, NV, DRZ, MK, HNN, DEFF, AHK, HCL, MLS, EYK, and WMO reviewed and edited the manuscript.

## Conflict of interest

The authors have declared that no conflict of interest exists.

## Funding support

This work is the result of NIH funding, in whole or in part, and is subject to the NIH Public Access Policy. Through acceptance of this federal funding, the NIH has been given a right to make the work publicly available in PubMed Central.

- NIH grant R01HL167718 (to WMO).

## Supplementary Material

Supplemental data

Unedited blot and gel images

Supporting data values

## Version 1 04/23/2026

In-Press Preview

## Version 2 07/08/2026

Electronic publication
