# Supplementary material for: TGF-β coordinates alanine synthesis and import for myofibroblast differentiation in pulmonary fibrosis
Source: JCI Insight. 2026 Apr 23;11(13):e199449. doi: 10.1172/jci.insight.199449 (PMC13460857; doi:10.1172/jci.insight.199449)
Supplement: Supplementary file 1 [file jciinsight-11-199449-s052.pdf]

Supplemental Information

TGF-β coordinates alanine synthesis and import for myofibroblast differentiation in pulmonary fibrosis

Fei Li, Niv Vigder, David R. Ziehr, Mari Kamiya, Hung N. Nguyen, Diana E. Ferreyra Faustino, Aseel H. Khalil, Hilaire C. Lam, Matthew L. Steinhauser, Edy Y. Kim, and William M. Oldham

Supplemental Figures 1-8

Supplemental Tables 1-5

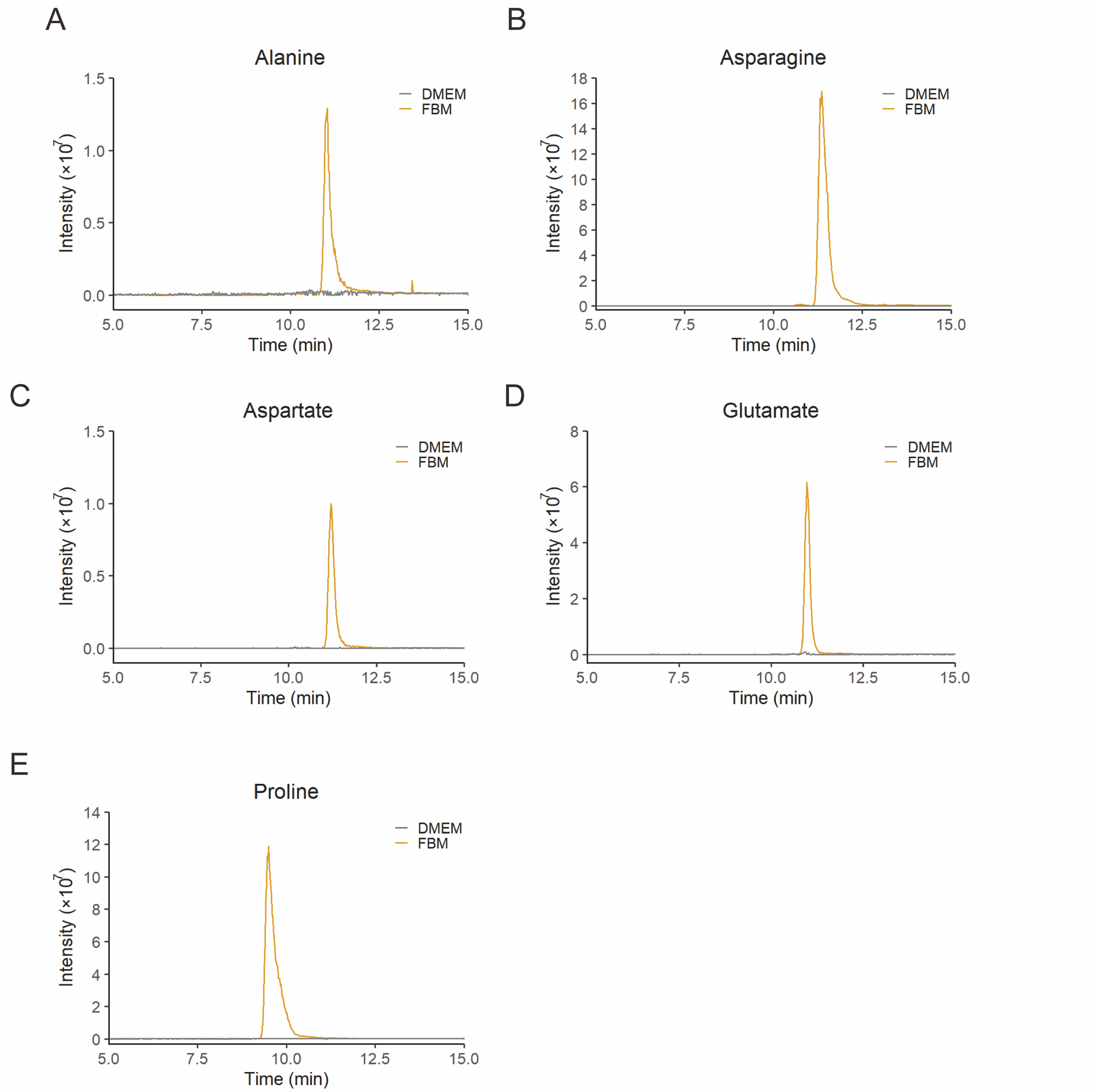

Supplemental Figure 1. Amino acid composition of DMEM and FBM. (A-E) Representative LC/MS chromatograms of selected NEAAs in FBM and DMEM. Shown are extracted ion chromatograms (± 3 ppm) for alanine (m/*z* 90.0550, RT 11.0 min), asparagine (m/*z* 133.0608, RT 11.4 min), aspartate (m/*z* 134.0448, RT 11.2 min), glutamate (m/*z* 148.0604, RT 11.0 min), and proline (m/*z* 116.0706, RT 9.5 min).

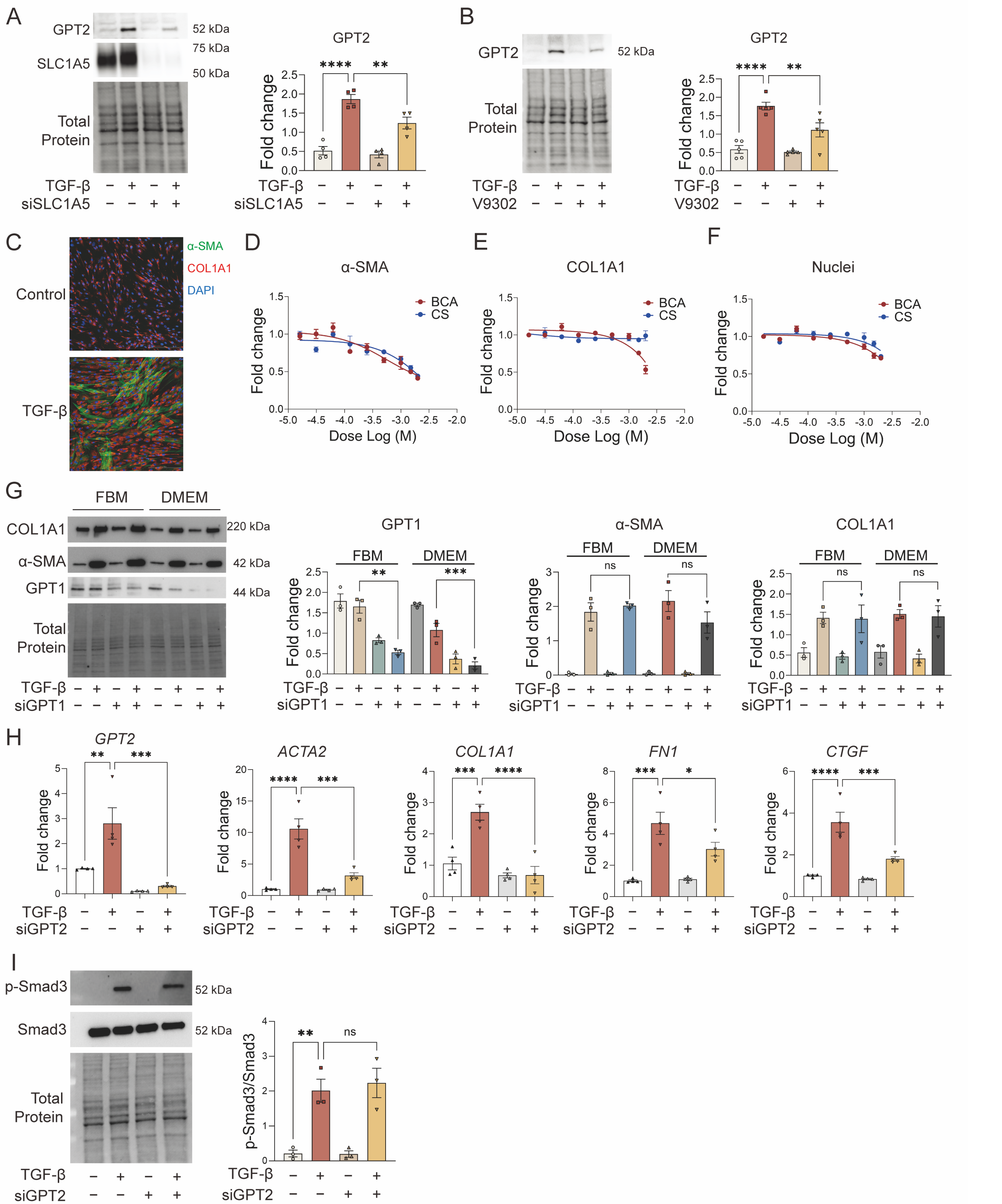

Supplemental Figure 2. Effects of GPT inhibition on myofibroblast differentiation. (A) Immunoblot analysis showing GPT2 protein expression following SLC1A5 knockdown under TGF-β stimulation for 48 h. (B) Immunoblot analysis showing GPT2 protein expression after pharmacologic inhibition with V-9302 (5 μM) under TGF-β stimulation for 48 h. (C) Representative immunofluorescence images showing α-SMA (green), COL1A1 (red), and DAPI (blue) in control and

TGF-β–treated NHLF. (D–F) Immunofluorescence quantification showing the dose-dependent effects of GPT1/2 inhibitors L-cycloserine (CS) and β-chloro-L-alanine (BCA) on α-SMA expression (D), COL1A1 expression (E), and cell number (F) in FBM-cultured NHLF after 48 h of treatment. (G) Western blot analysis showing that GPT1 knockdown does not significantly affect α-SMA or COL1A1 expression in either FBM or DMEM medium. (H) qPCR analysis showing relative mRNA expression (fold change, normalized to the internal reference gene) of *GPT2*, *ACTA2* (encoding α-SMA), *COL1A1*, *FN1* (encoding FN), and *CTGF* in control and GPT2 knockdown NHLF cultured in DMEM with or without TGF-β stimulation for 48 h. (I) Immunoblot analysis showing that GPT2 knockdown does not alter Smad3 phosphorylation (p-Smad3) following 1 h TGF-β stimulation. Quantification of the p-Smad3/Smad3 ratio is shown. For Western blot analysis (A, B, G, I), individual data points represent biological replicates. For immunofluorescence (C-F) and qPCR (H) analyses, representative results from three independent experiments are shown. A, B, G-I: One-way ANOVA; data are presented as mean ± SEM; ns $p \geq 0.05$, * $p < 0.05$, ** $p < 0.01$, *** $p < 0.001$, **** $p < 0.0001$.

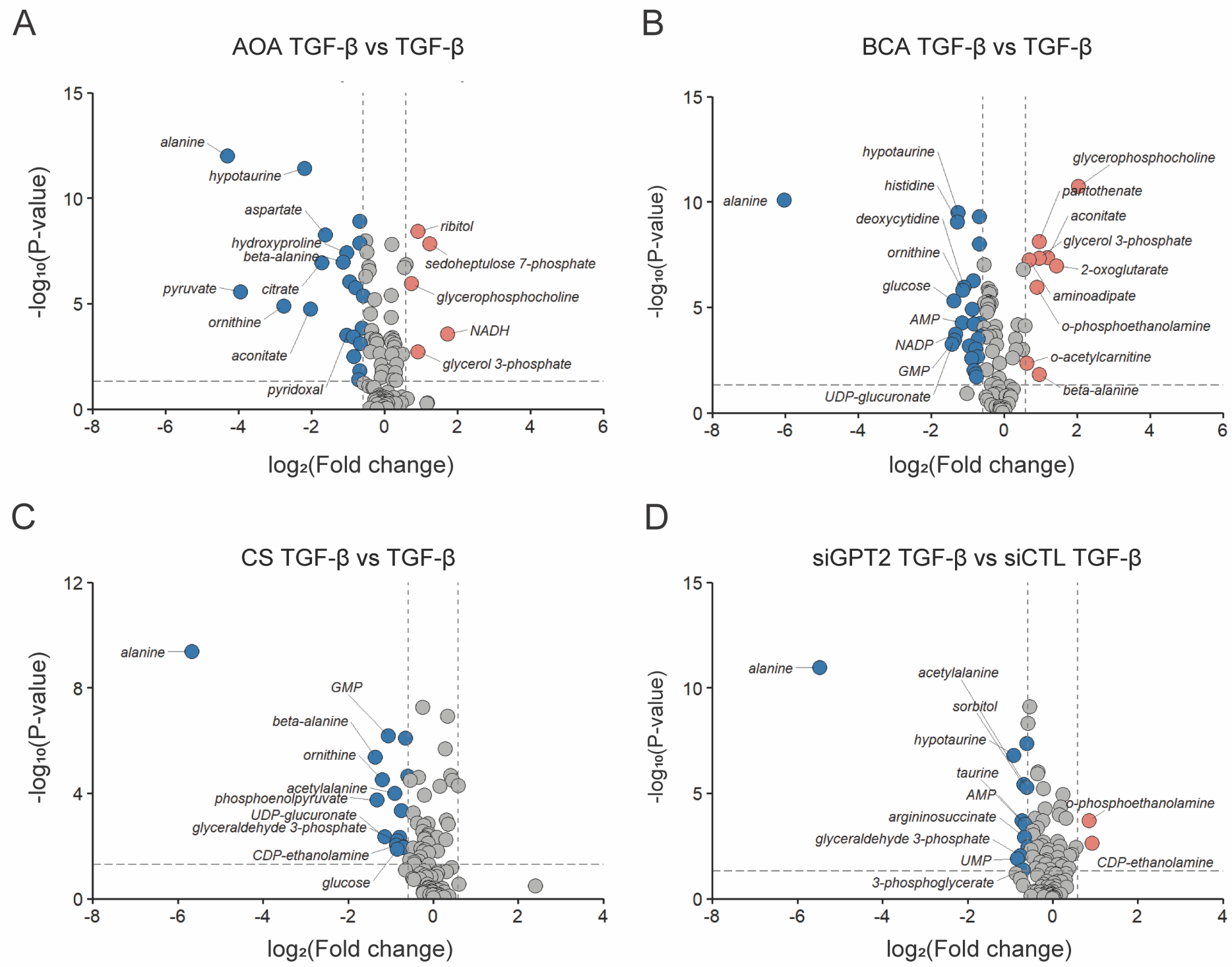

Supplemental Figure 3. Effects of GPT2 inhibition on profibrotic metabolites. (A–D) Volcano plots showing metabolomic changes in TGF-β–treated NHLF upon inhibition or knockdown of GPT2 using AOA (A), BCA (B), CS (C), or GPT2 siRNA (D). Differential metabolites were selected based on a fold change (FC) threshold ⩾ 1.5 and a p-value ⩽ 0.05. The top 10 most significantly altered metabolites are labeled.

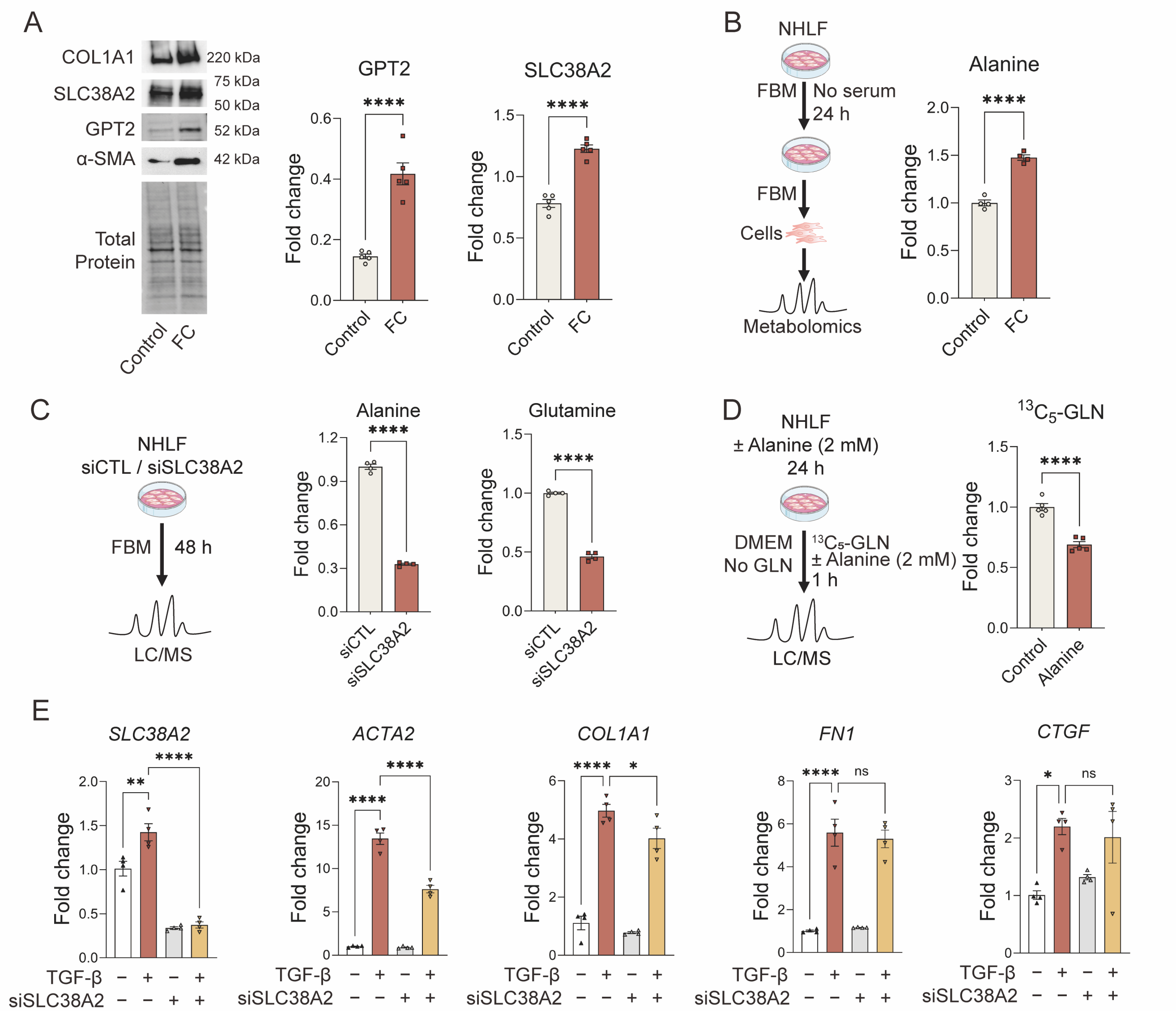

Supplemental Figure 4. SLC38A2 mediates alanine-supported myofibroblast activation and modulates glutamine uptake. (A) Immunoblot analysis showing protein expression of α-SMA, COL1A1, GPT2, and SLC38A2 in NHLF treated with pro-fibrotic cocktail (FC; TGF-β 5 ng/mL, PDGF-AB 10 ng/mL, TNF-α 10 ng/mL, LPA 5 μM). Quantification of GPT2 and SLC38A2 protein levels are shown. (B) Schematic of the experimental design and LC/MS-based metabolomic analysis showing intracellular alanine levels in NHLF cultured in FBM and treated with FC for 48 h. (C) Experimental schematic and quantification of intracellular alanine and glutamine levels in control and SLC38A2-knockdown NHLF after 48 h of culture in FBM. (D) Schematic of the experimental design to assess the effect of extracellular alanine (2 mM) on [$^{13}C_5$]-glutamine uptake in glutamine-deficient DMEM. Quantification of intracellular $^{13}C_5$-glutamine levels is shown on the right. (E) qPCR analysis showing relative mRNA expression (fold change, normalized to the internal reference gene) of *SLC38A2*, *ACTA2* (encoding α-SMA), *COL1A1*, *FN1* (encoding FN), and *CTGF* in control or SLC38A2 knockdown NHLF cultured in FBM under TGF-β stimulation for 48 h. For Western blot analysis (A), individual data points represent biological replicates. For qPCR analysis(E), representative results from 3 independent experiments are shown. A-D: unpaired *t*-test; E: One-way ANOVA. Data are presented as mean ± SEM; ns $p \geq 0.05$, * $p < 0.05$, ** $p < 0.01$, *** $p < 0.001$, **** $p < 0.0001$.

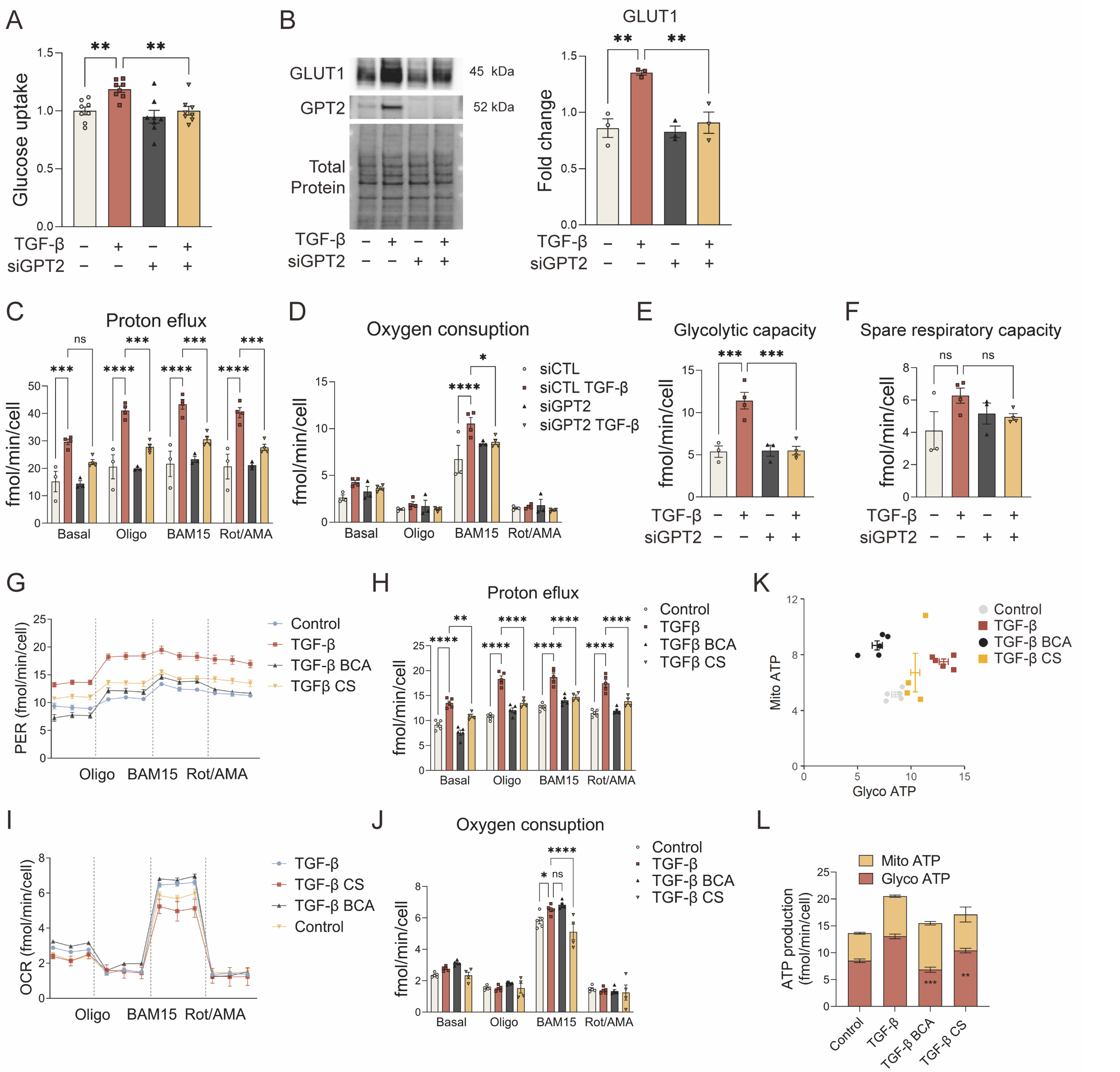

Supplemental Figure 5. GPT2 inhibition disrupts cellular bioenergetics and anabolic metabolism. (A) Quantification of glucose uptake assessed by 2-NBDG (60 µM for 30 min) fluorescence intensity (normalized to cell count) in control and siGPT2 NHLF with or without TGF-β stimulation in DMEM. (B) Immunoblot analysis of GLUT1 protein expression in control or GPT2 knockdown NHLF with or without TGF-β stimulation. Quantification of GLUT1 protein levels is shown on the right. (C, D) Summary of proton efflux (PER) and oxygen consumption rates (OCR) from Seahorse XF analysis in NHLF with or without GPT2 knockdown and TGF-β treatment. Quantification is based on Fig. 5A and 5B. (E, F) Cell bioenergetic stress test results: glycolytic capacity was determined by the increase in proton efflux rate after oligomycin treatment; spare respiratory capacity was calculated as the increase in OCR after BAM15 treatment relative to basal levels. (G–L) PER (G), summary PER analysis (H), OCR (I), summary OCR analysis (J), glycolytic and mitochondrial ATP production rates (K), and summarized ATP production (L) in NHLF after 48 h of TGF-β treatment in DMEM with or without pharmacological GPT inhibitor (BCA and CS). For Western blot analysis (B), individual data points represent biological

replicates. For glucose uptake analysis (A) and Seahorse analyses (C–L), representative results from three independent experiments are shown. A, B, E, F: One-way ANOVA; C, D, H, L: Two-way ANOVA. Data are presented as mean ± SEM; ns $p \geq 0.05$, * $p < 0.05$, ** $p < 0.01$, *** $p < 0.001$, **** $p < 0.0001$.

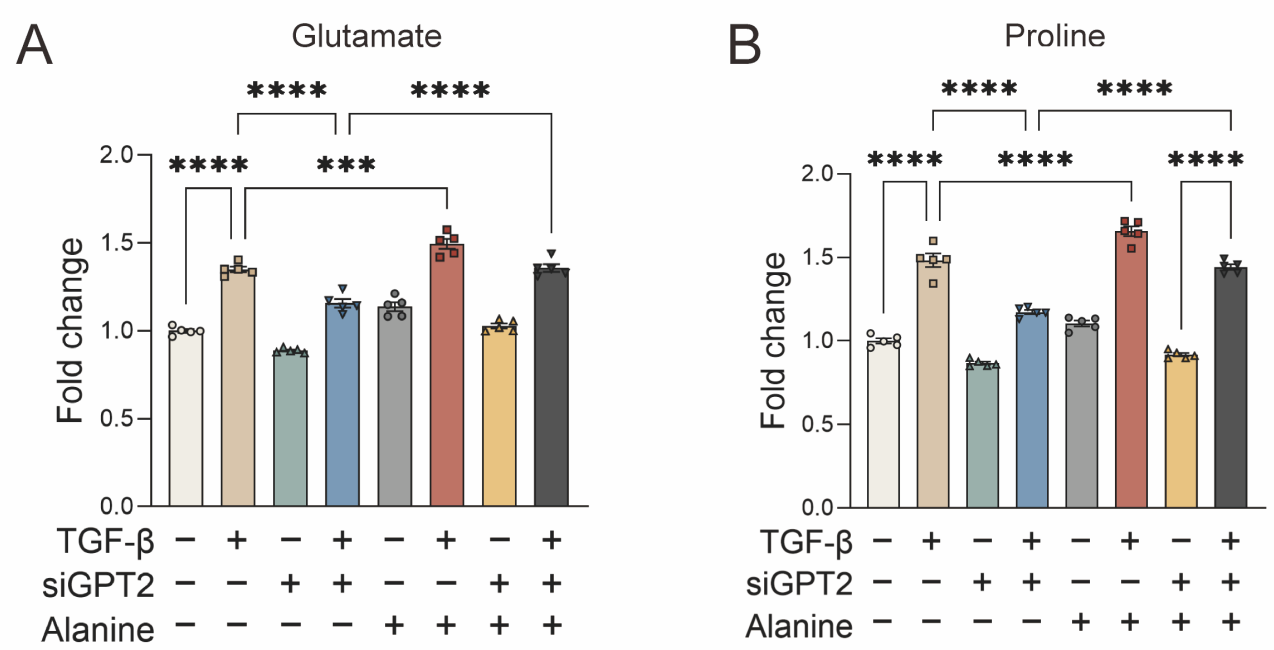

Supplemental Figure 6. Alanine deprivation disrupts glutamate and proline metabolic pathways. (A, B) Relative quantification of intracellular glutamate (A), and proline (B) levels in GPT2 knockdown NHLF cultured in DMEM with TGF-β (48 h) and supplemented with alanine (2 mM). A and B: One-way ANOVA. Data are presented as mean ± SEM; *** $p < 0.001$, **** $p < 0.0001$.

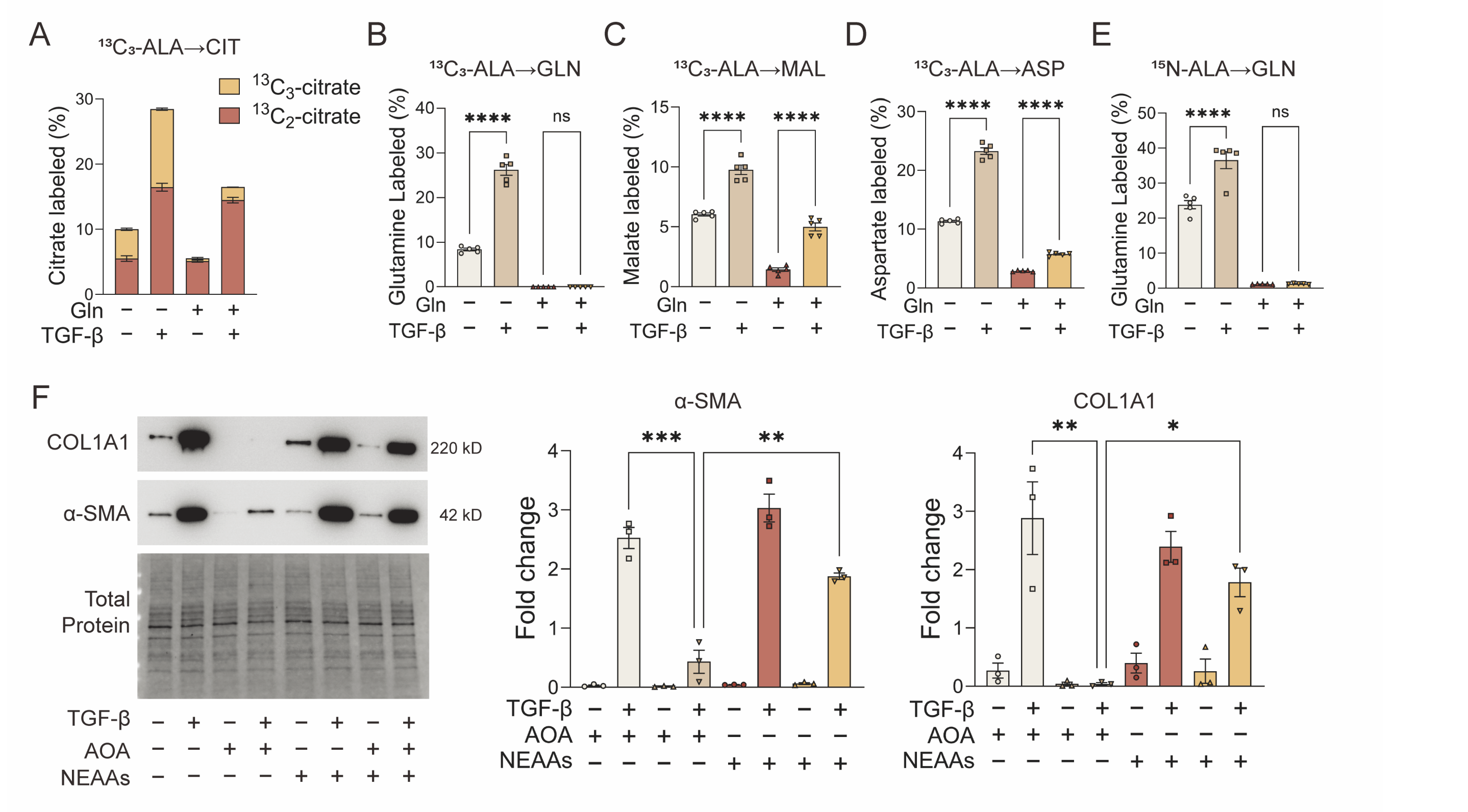

Supplemental Figure 7. Alanine is a carbon and nitrogen source for anabolic metabolism during myofibroblast differentiation. (A-D) Metabolite labeling from [U-$^{13}C_3$]-alanine showing fractional label enrichment in intracellular $^{13}C_2$- or $^{13}C_3$-citrate (A), glutamine (B), malate (C), and aspartate (D), with or without TGF-β treatment in the presence or absence of exogenous glutamine. (E) Fractional label enrichment of intracellular glutamine from ^{15}N-alanine. (F) Representative Western blot and quantification showing α-SMA and COL1A1 expression in NHLF cultured in DMEM with TGF-β (2 ng/mL) for 48 h, in the presence or absence of AOA (1 mM) and/or NEAA cocktail supplementation. For Western blot analysis (F), individual data points represent biological replicates. A-F: One-way ANOVA. Data are presented as mean ± SEM; ns $p \geq 0.05$, * $p < 0.05$, ** $p < 0.01$, *** $p < 0.001$, **** $p < 0.0001$.

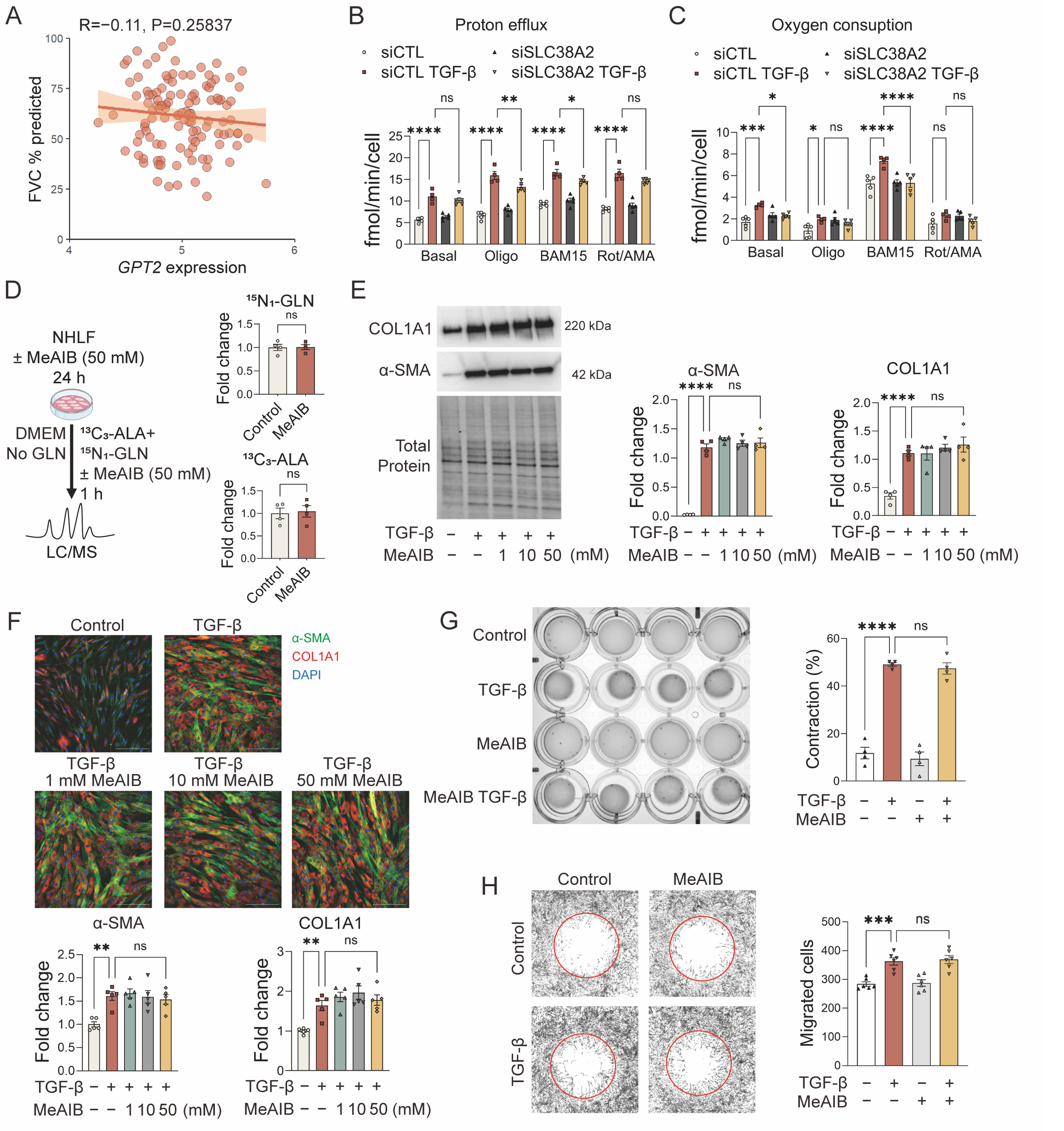

Supplemental Figure 8. SLC38A2–alanine axis regulates fibroblast metabolic activation and pro-fibrotic phenotypes. (A) Pearson correlation between GPT2 mRNA expression and predicted forced vital capacity (FVC) in patients (GSE32537). (B–C) Seahorse extracellular flux analysis showing PER (B) and OCR (C) in control or SLC38A2 knockdown NHLF with or without TGF-β stimulation in FBM for 48 h. (D) Schematic of isotope tracing experimental design and LC/MS analysis of $^{15}N_1$-glutamine and $^{13}C_3$-alanine uptake in control or MeAIB -treated NHLF for 1 h. ($^{15}N_1$-glutamine 2 mM, $^{13}C_3$-alanine 2 mM, MeAIB 50 mM). (E) Immunoblot analysis of α-SMA and COL1A1 protein expression in NHLF treated with TGF-β in the presence or absence of MeAIB (1, 10, or 50 mM) in FBM for 48 h. (F) Representative immunofluorescence images of α-SMA (green), COL1A1 (red), and DAPI (blue) in IPF LF cells treated with TGF-β in the

presence or absence of MeAIB (1, 10, or 50 mM) in FBM for 48 h, with quantification of relative fluorescence intensity (normalized to cell count). (G) Representative images of collagen gel contraction after 24 h TGF-β treatment with or without MeAIB (50 mM) in FBM. (H) Representative images of control or MeAIB (50 mM)-treated NHLF migrating into the cell-free zone 24 h after TGF-β treatment in FBM. For Western blot analysis (E), individual data points represent biological replicates. For Seahorse analyses (B, C), immunofluorescence (F), gel contraction (G), and migration assays (H), representative data from 3 independent experiments are shown. A: linear regression and Pearson correlation. B, C: two-way ANOVA. D: unpaired t-test. E-H: One-way ANOVA. Data are presented as mean ± SEM; ns $p \geq 0.05$, * $p < 0.05$, ** $p < 0.01$, *** $p < 0.001$, **** $p < 0.0001$.

Supplemental Table 1. Amino acid composition of DMEM

**Amino Acid**	**Concentration (mM)**
Alanine	
L-Arginine	0.398
Asparagine	
Aspartate	
L-Cystine	0.201
Glutamate	
L-Glutamine	4.000
Glycine	0.400
L-Histidine	0.200
L-Isoleucine	0.802
L-Leucine	0.802
L-Lysine	0.798
L-Methionine	0.201
L-Phenylalanine	0.400
Proline	
L-Serine	0.400
L-Threonine	0.798
L-Tryptophan	0.078
L-Tyrosine	0.398
L-Valine	0.803

Supplemental Table 2. Non-essential amino acid supplement composition

Amino Acid	Concentration (µM)
L-Alanine	100
L-Asparagine	100
L-Aspartate	100
L-Proline	100
Glutamate	100
L-Serine	100
L-Glycine	100

Supplemental Table 3. Amino acid composition of DMEM/F-12

Amino Acid	Concentration (mM)
L-Alanine	0.050
L-Arginine	0.699
L-Asparagine	0.050
L-Aspartic acid	0.050
L-Cysteine	0.100
L-Cystine	0.100
L-Glutamic acid	0.050
L-Glutamine	2.500
Glycine	0.250
L-Histidine	0.150
L-Isoleucine	0.416
L-Leucine	0.451
L-Lysine	0.499
L-Methionine	0.116
L-Phenylalanine	0.215
L-Proline	0.150
L-Serine	0.250
L-Threonine	0.449
L-Tryptophan	0.044
L-Tyrosine	0.214
L-Valine	0.452

Supplemental Table 4. Key resources

Reagent or resource	Source	Identifier
Anti-SLC38A2	MBL International	BMP081
Anti-GPT (GPT1)	Proteintech	16897-1-AP
Anti-GPT2	Proteintech	16757-1-AP
Anti-FN	Abcam	ab2413
Anti-α-SMA	Abcam	ab7817
Anti-Smad3	Cell Signaling Technology	9523
Anti-p-Smad3	Cell Signaling Technology	9520
Anti-COL1A1	Cell Signaling Technology	39952
Anti-GLUT1	Cell Signaling Technology	12939
Anti-SLC1A5	Cell Signaling Technology	8057
HRP-conjugated anti-Rabbit IgG	Cell Signaling Technology	7074
HRP-conjugated anti-Mouse IgG	Cell Signaling Technology	7076
Goat anti-mouse Alexa Fluor 488	Thermo Fisher Scientific	A11001
Goat anti-rabbit Alexa Fluor 647	Thermo Fisher Scientific	A21244
Recombinant human TGF-β1	PeproTech	100-21
Recombinant human PDGF-AB	PeproTech	100-00AB
Recombinant human TNF-α	PeproTech	300-01A
LPA	Cayman Chemical	62215
β-Chloro-L-Alanine (BCA)	MedChemExpress	HY-107373
L-Cycloserine (CS)	Sigma-Aldrich	C1159
Aminooxyacetic acid (AOA)	SelleckChem	S4989
[U-$^{13}C_6$]-Glucose	Cambridge Isotope Laboratories	71800
[U-$^{13}C_5$]-Glutamine	Cambridge Isotope Laboratories	CLM-1822-H
[α-$^{15}N_1$]-Glutamine	Cambridge Isotope Laboratories	NLM-557, NLM-1016
[U-$^{13}C_3$]-L-Alanine	Cambridge Isotope Laboratories	CLM-2184-H
[^{15}N]-L-Alanine	Cambridge Isotope Laboratories	NLM-454
L-glutamine	Sigma-Aldrich	G8540
L-alanine	Sigma-Aldrich	A7627
L-proline	Sigma-Aldrich	5607
L-aspartate	Sigma-Aldrich	7219
L-glutamate	Sigma-Aldrich	G8415
L-asparagine	Sigma-Aldrich	A4159
DNase I	Sigma-Aldrich	10104159001
Liberase	Sigma-Aldrich	05401020001
Bovine serum albumin	Sigma-Aldrich	A9576

Halt™ Protease and Phosphatase Inhibitor Cocktail	Thermo Fisher Scientific	78440
Lipofectamine RNAiMAX transfection reagent	Thermo Fisher Scientific	13778
WesternBright ECL reagent	Advansta	K-12045-D20
Nuclear Green LCS1	Abcam	ab138904
BAM15	Cayman	17811
Pierce™ BCA Protein Assay Kit		23227
Trans-Blot Turbo PVDF Transfer Kit	Bio Rad	1704272
Oris Cell Migration Assay Kit	Platypus Technologies	CMA1.101
QIAGEN RNeasy Mini Kit	Qiagen	74106
SYBR Green PCR Master Mix	Thermo Fisher Scientific	A25742
PrimeScript RT Reagent Kit	Takara	RR037A
Glucose Uptake Cell-Based Assay Kit	Cayman Chemical	600470
LDH-Glo™ Cytotoxicity Assay	Promega	J2380
Primary normal human lung fibroblasts (NHLF)	Lonza	CC-2512
Precision-Cut Lung Slices (PCLS)	Anobios	
ON-TARGETplus GPT2 siRNA	Dharmacon	L-004173-01-0005
Non TARGETplus Control siRNA	Dharmacon	D-001810-10-05
ON-TARGETplus SLC38A2 siRNA	Dharmacon	L-007559-01-0005
ON-TARGETplus GPT siRNA	Dharmacon	L-031622-01-0005
ON-TARGETplus SLC1A5 siRNA	Dharmacon	L-007429-00-0005

Supplemental Table 5. Primer sequences used for qPCR analysis

Gene	Forward (5′–3′)	Reverse (5′–3′)
GPT2	GGAGCTAGTGACGGCATTTCTACGA	CCCAGGGTTGATTATGCAGAGCA
SLC38A2	GTGTTAATGGCTGTGACCCTGAC	GAGACTATGACGCCACCAACTGA
ACTA2	CTTCGTTACTACTGCTGAGCGTGAG	GGGGCAATGATCTTGATCTTCATGG
FN1	GCCAAGAGACAGCTGTAACCCAG	CAATGCACTGATCTCGAAGCTGC
COL1A1	TTCAGGGAATGCCTGGTGAA	ACCTTTGGGACCAGCATCA
CTGF	CAGCATGGACGTTCGTCTG	AACCACGGTTTGGTCCTTGG
GAPDH	GGTGAAGGTCGGAGTCAACGGA	GAGGGATCTCGCTCCTGGAAGA
HPRT	AAGGACCCCACGAAGTGTTG	GGCTTTGTATTTTGCTTTTCCA
