# Supplementary material for: TGF-β coordinates alanine synthesis and import for myofibroblast differentiation in pulmonary fibrosis
Source: JCI Insight. 2026 Apr 23;11(13):e199449. doi: 10.1172/jci.insight.199449 (PMC13460857; doi:10.1172/jci.insight.199449)

Full unedited gels for Fig 2B

GPT2 band

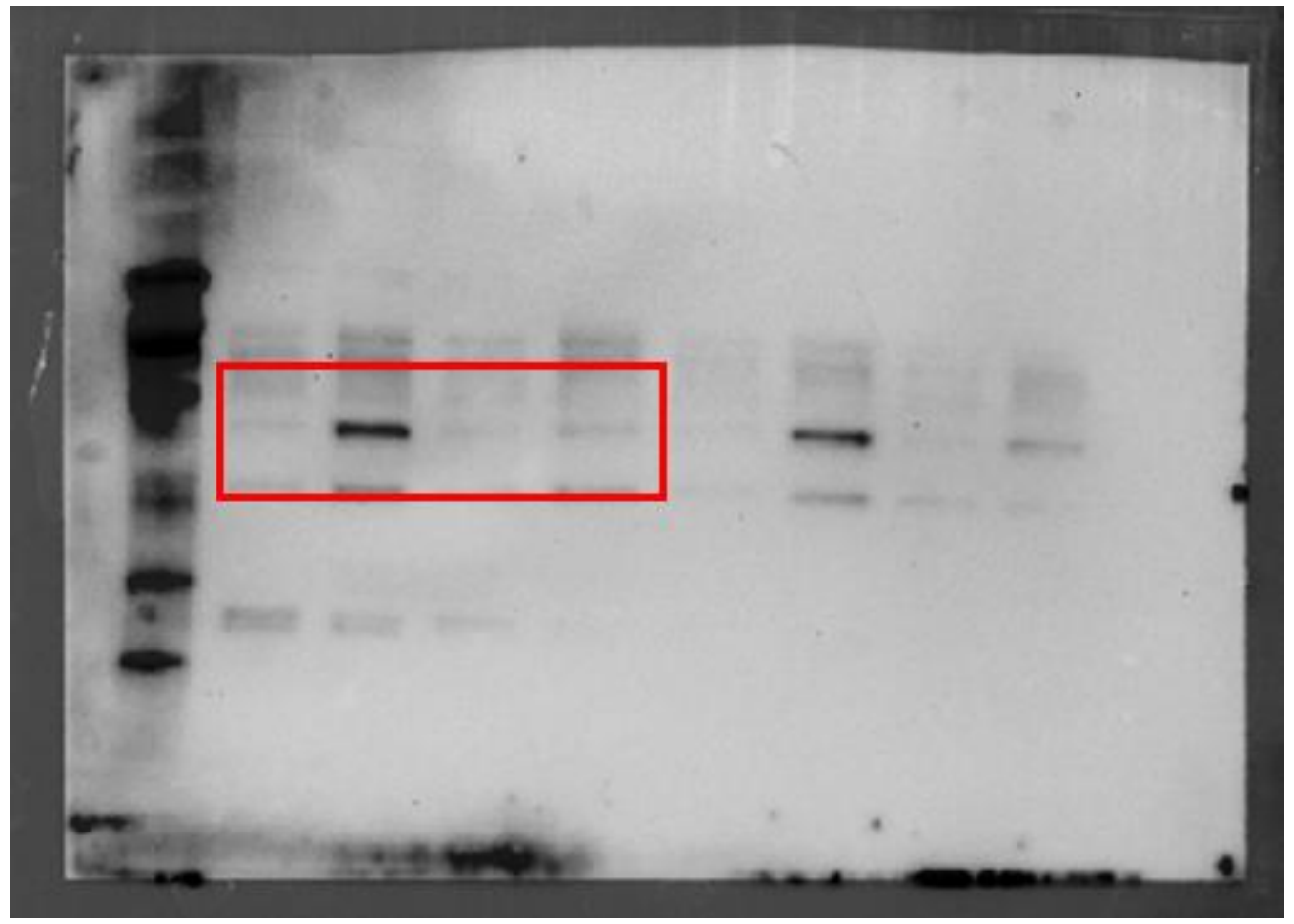

Total Protein

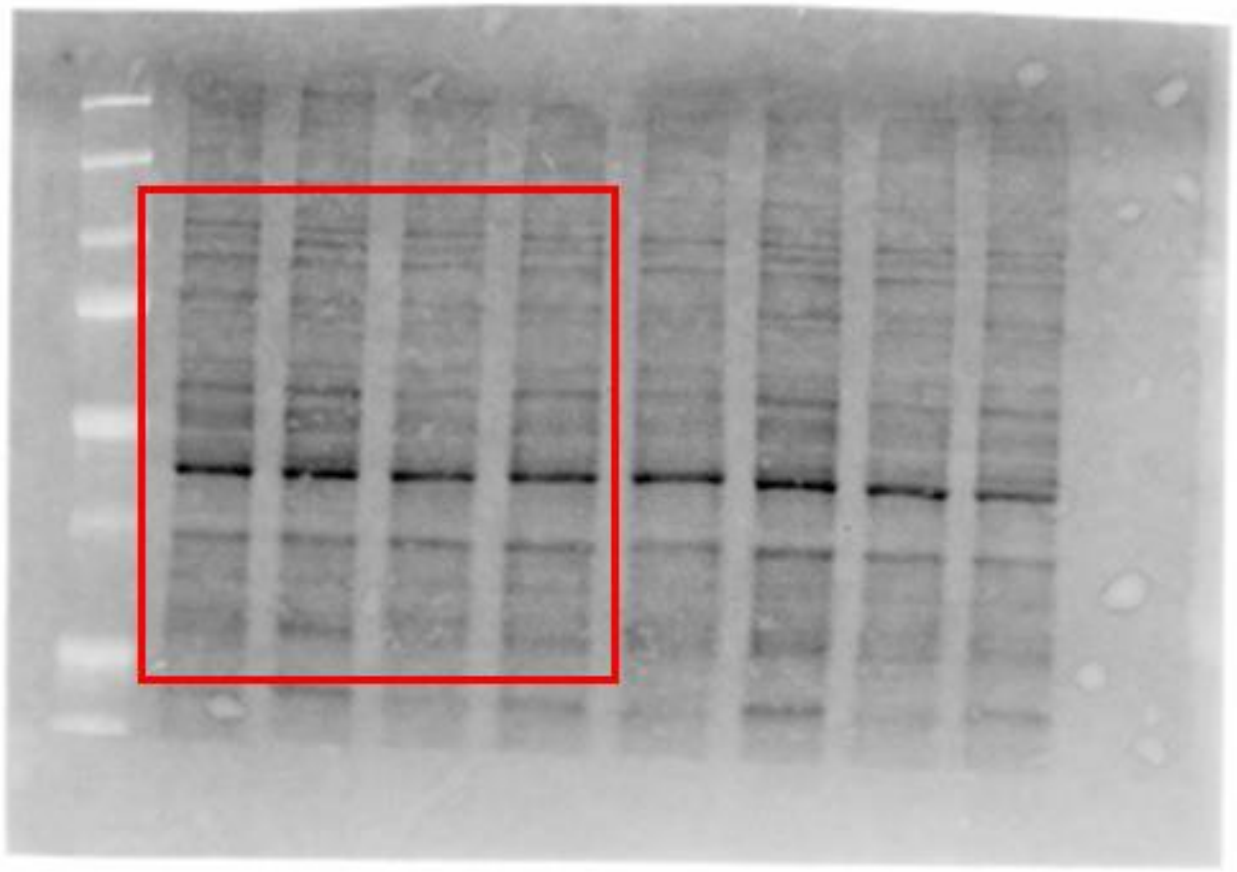

Full unedited gels for Fig 2C

GPT1 band

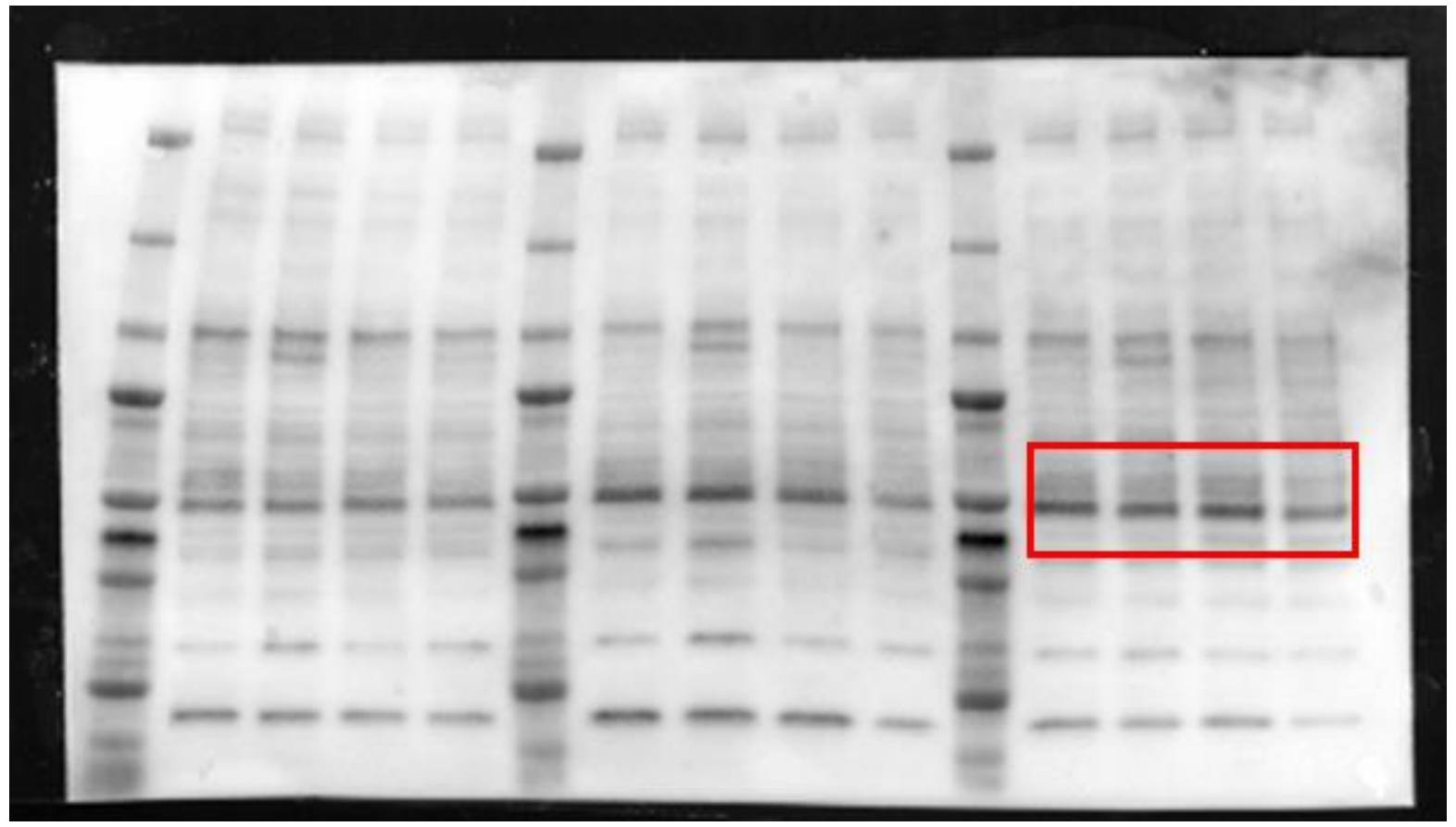

Total Protein

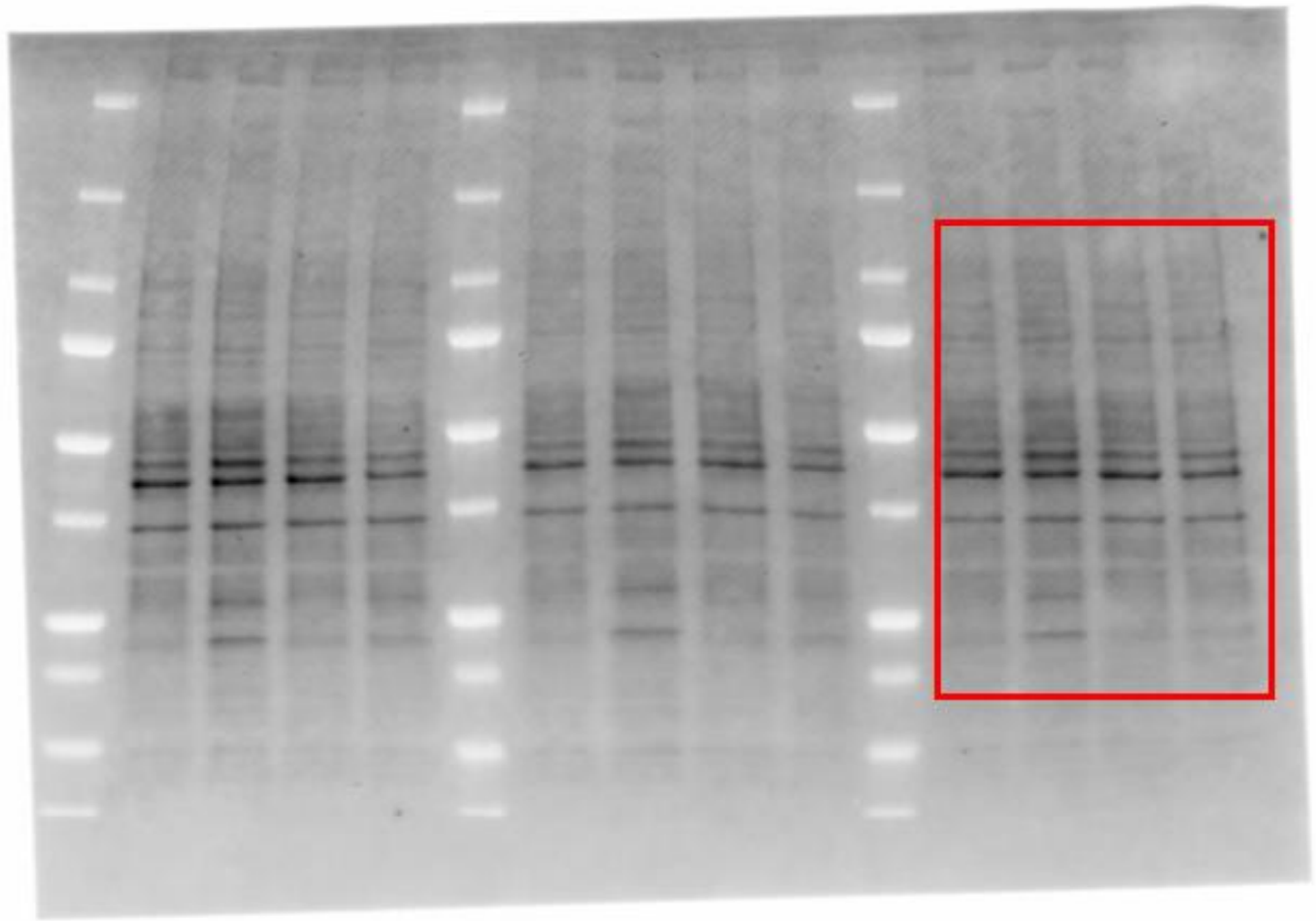

Full unedited gels for Fig. 2D

GPT2 band

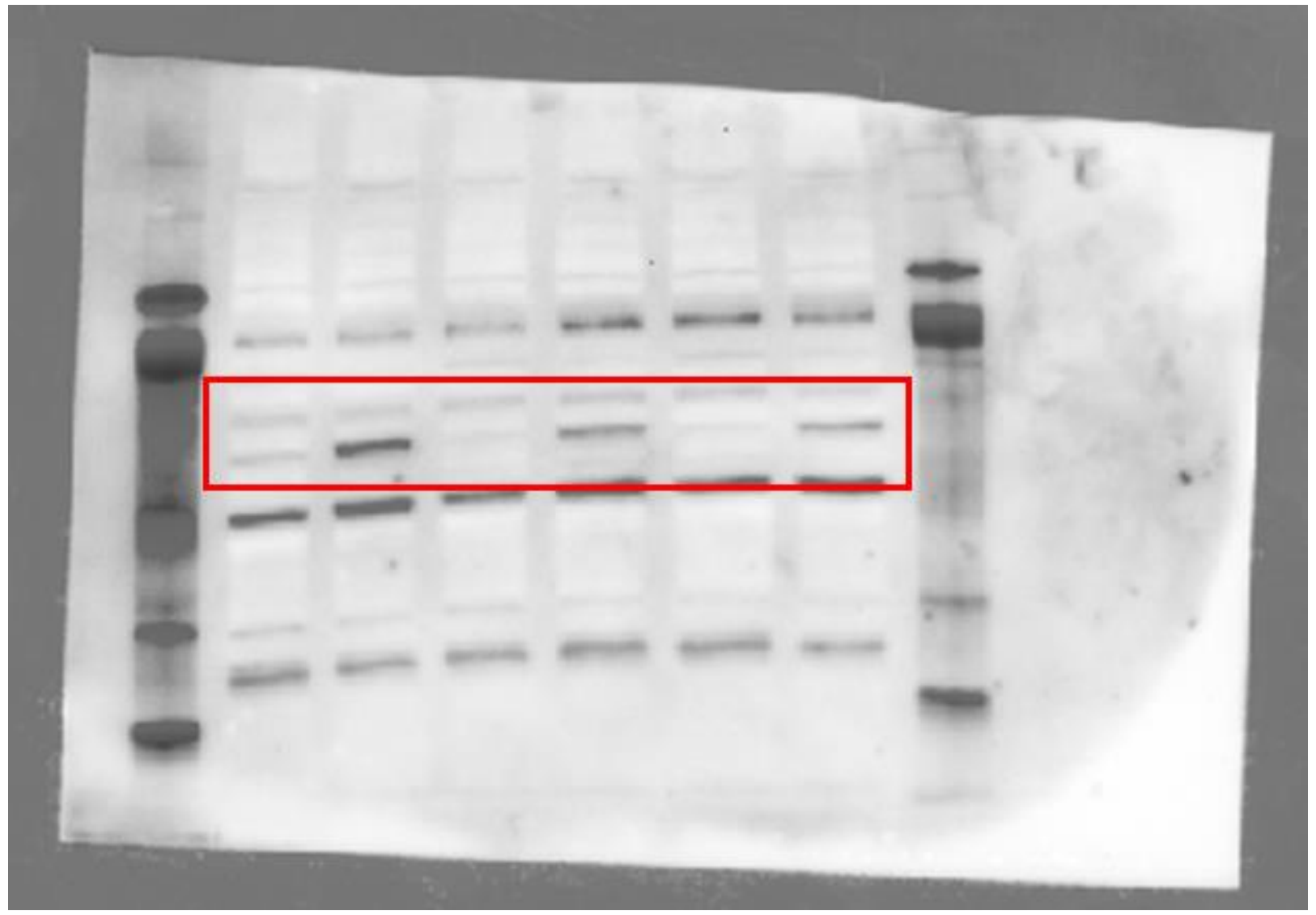

Total Protein

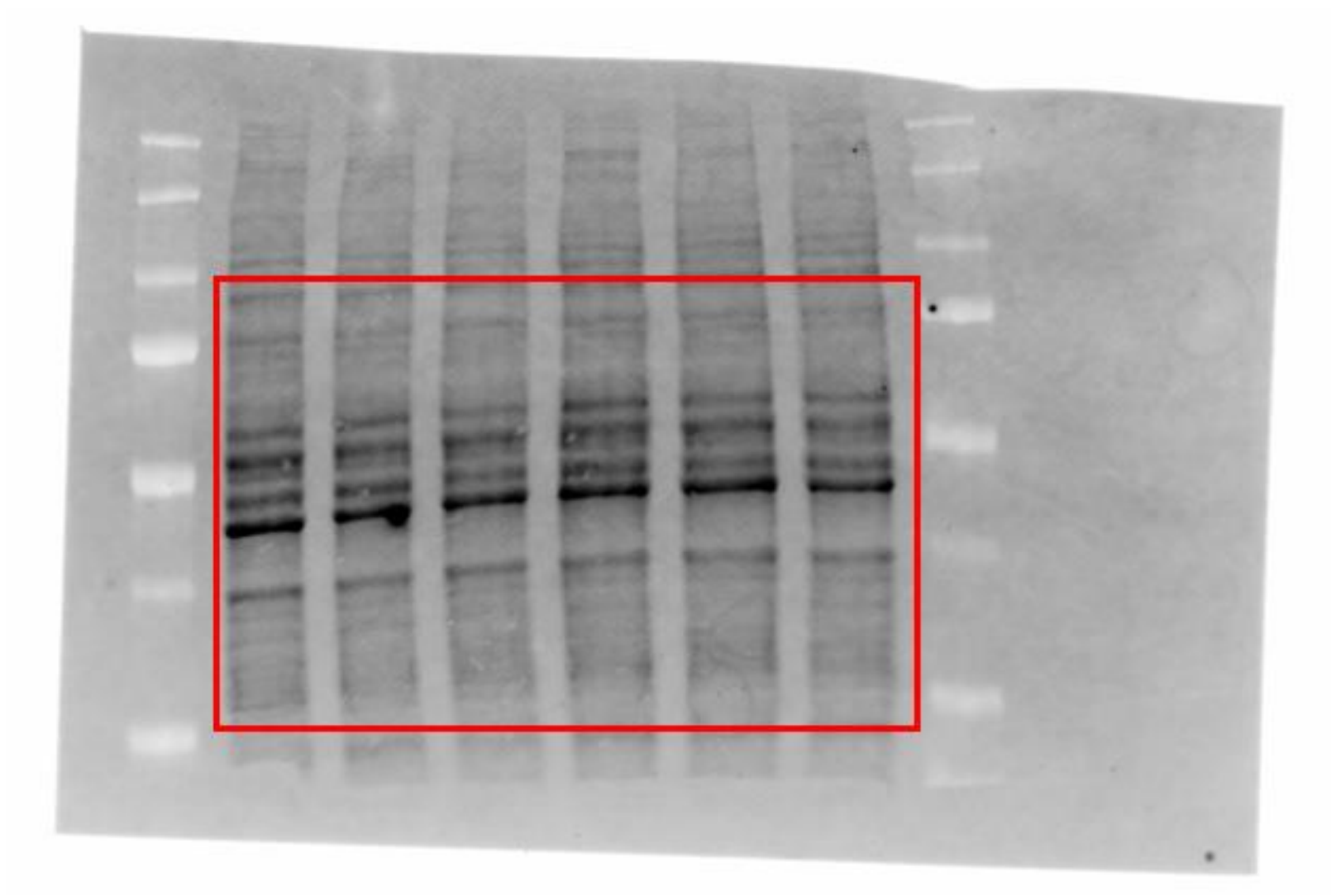

Full unedited gels for Fig. 2E

GPT2 band

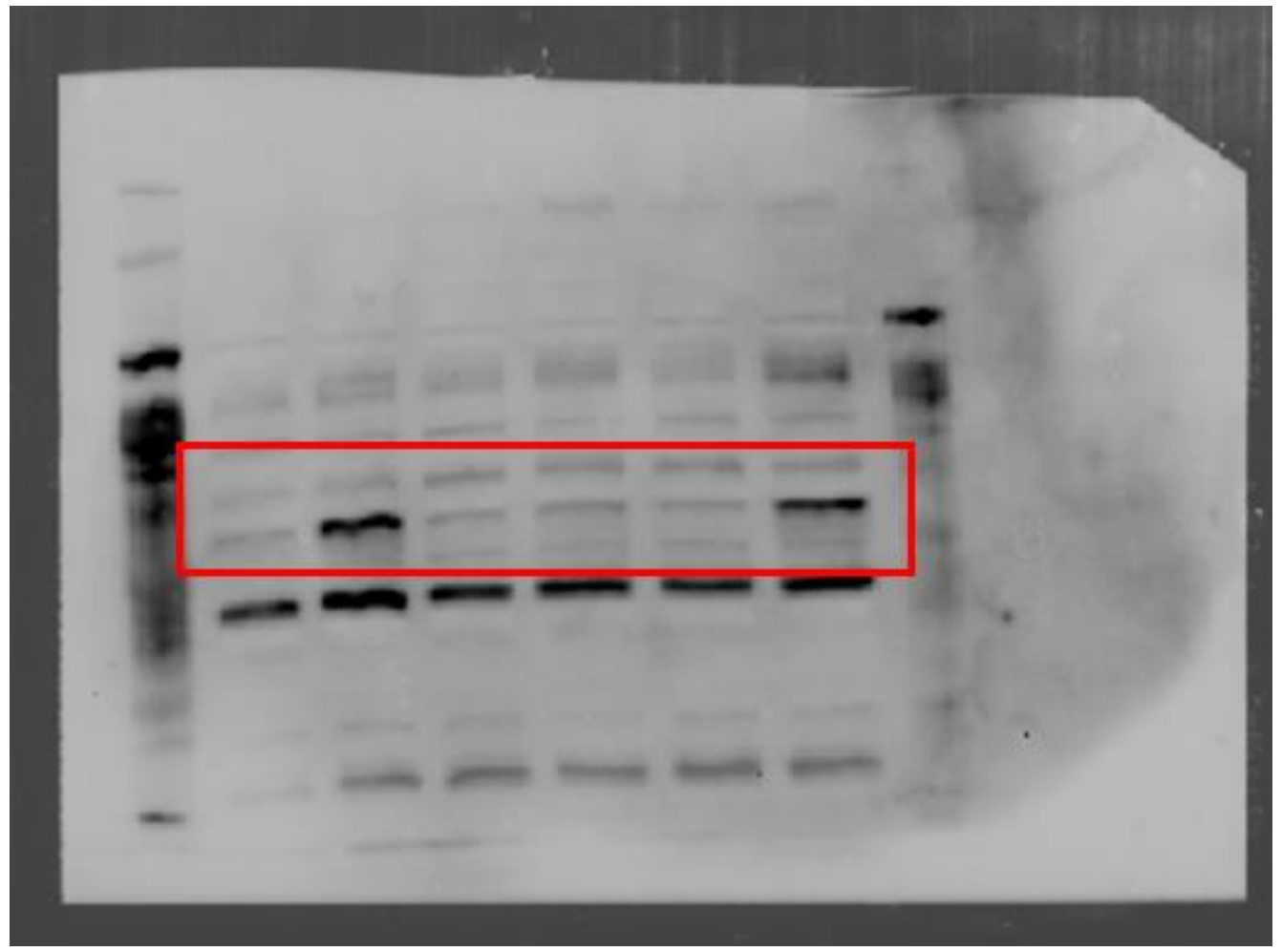

Total Protein

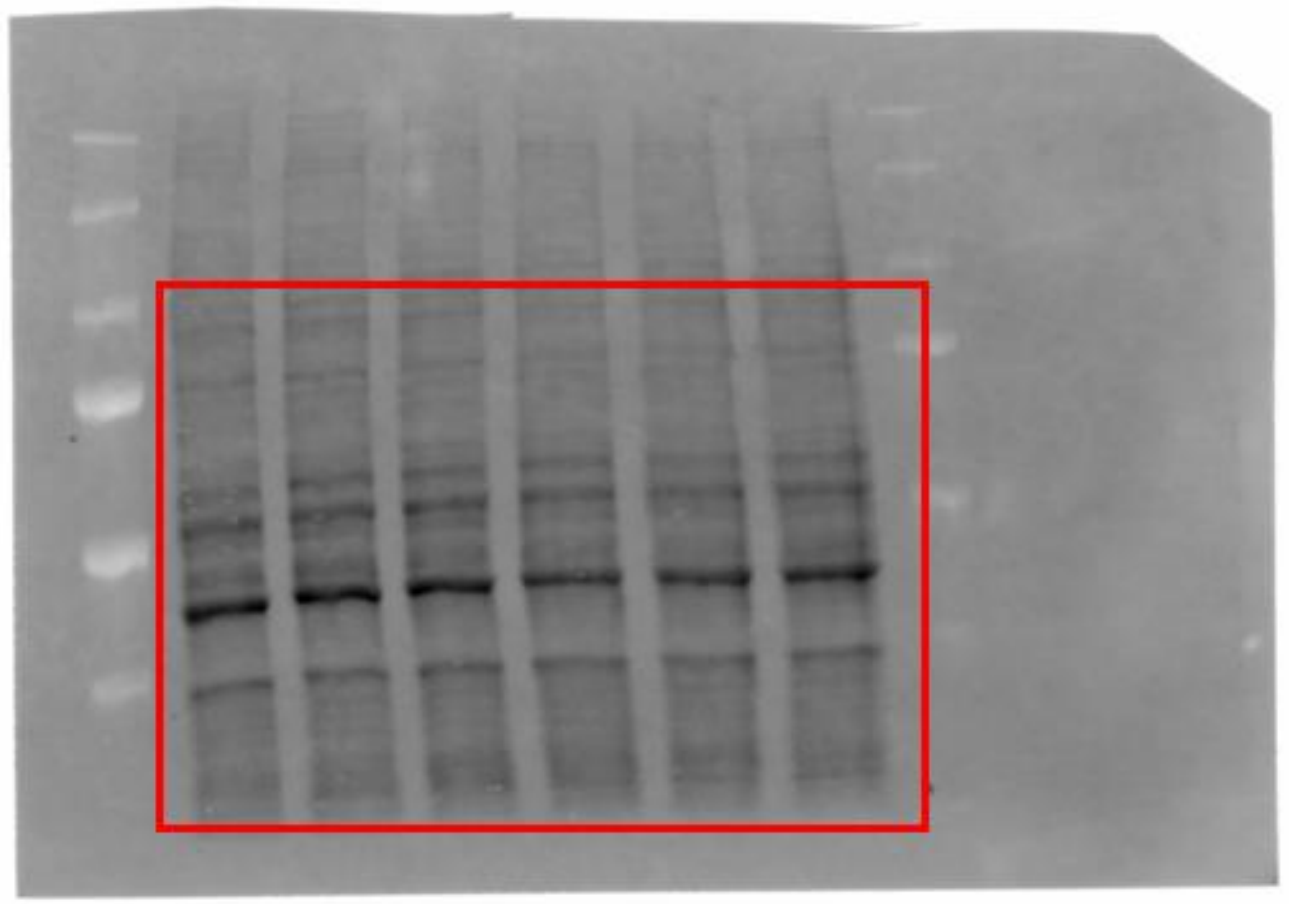

Full unedited gels for Fig. 2G

COL1A1 band

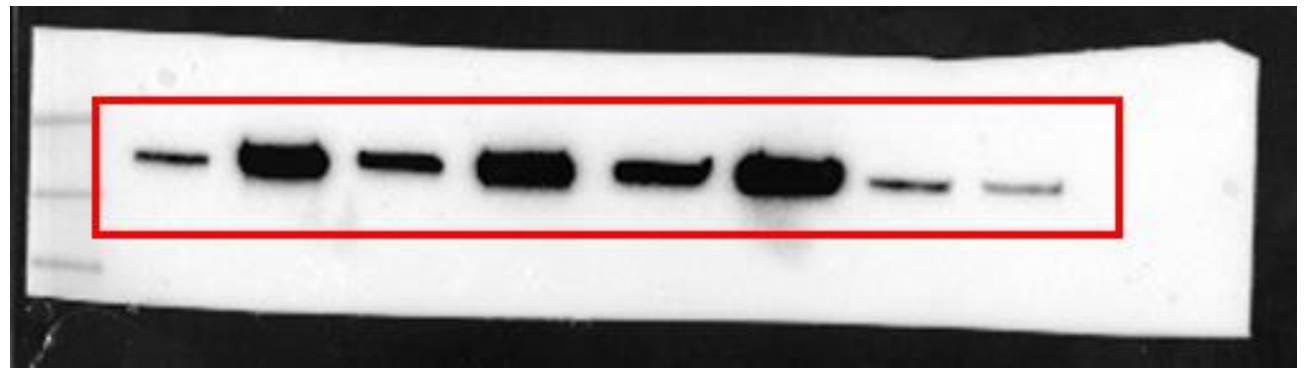

α-SMA band

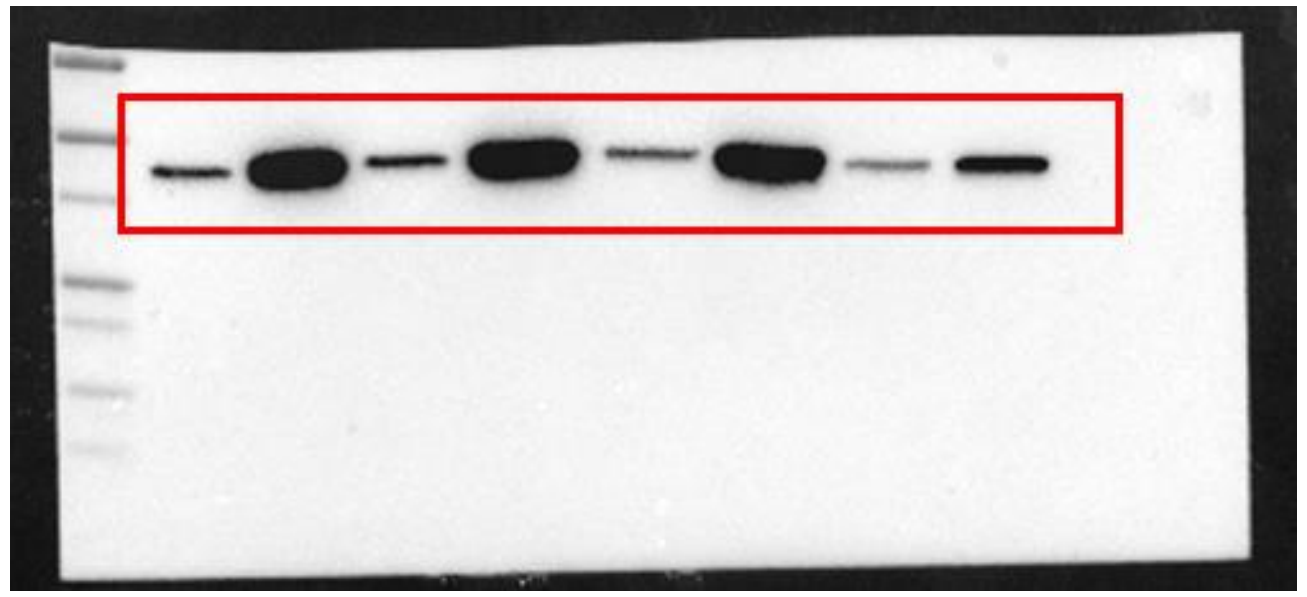

Total Protein

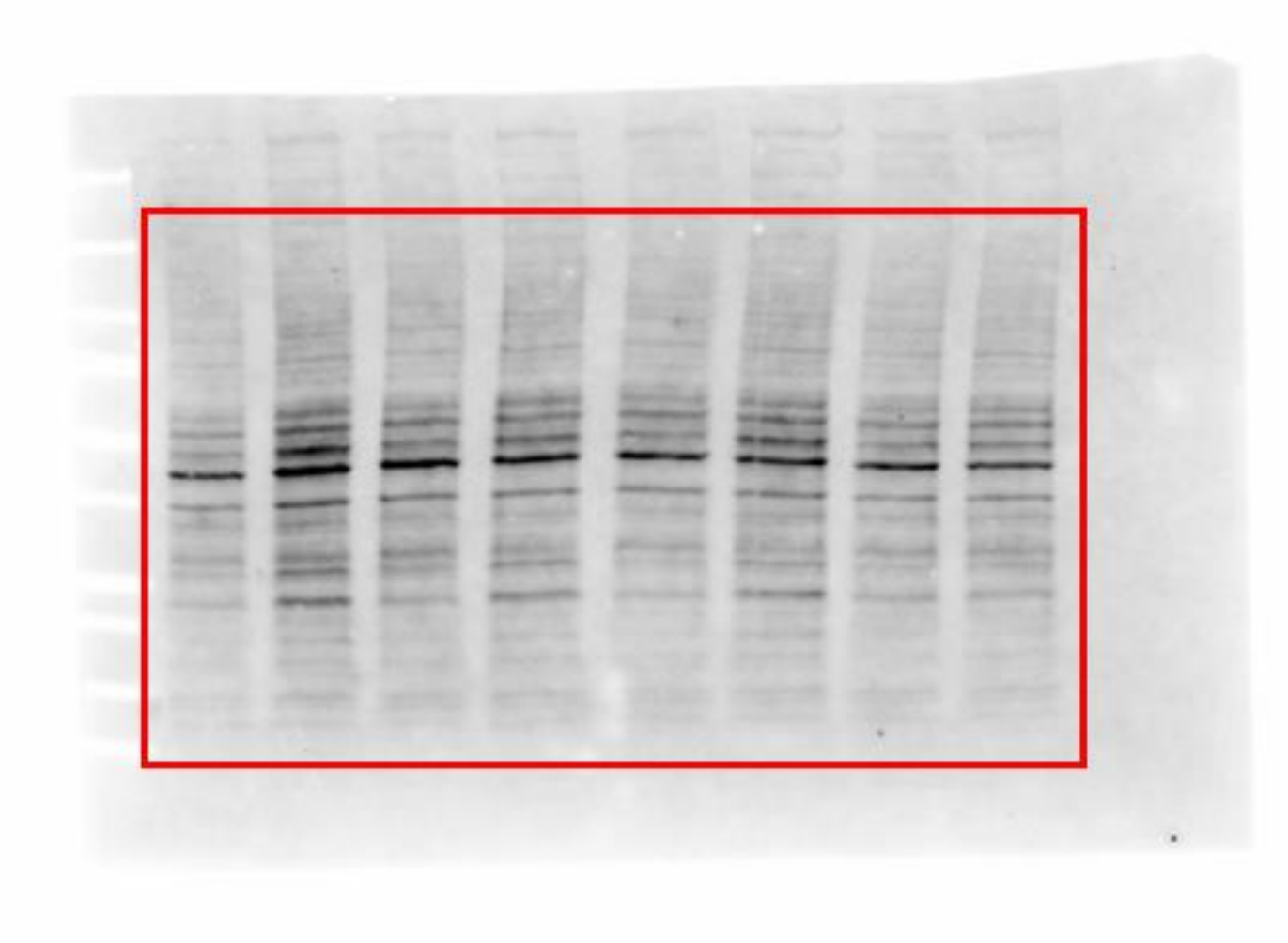

Full unedited gels for Fig. 2I

COL1A1 band

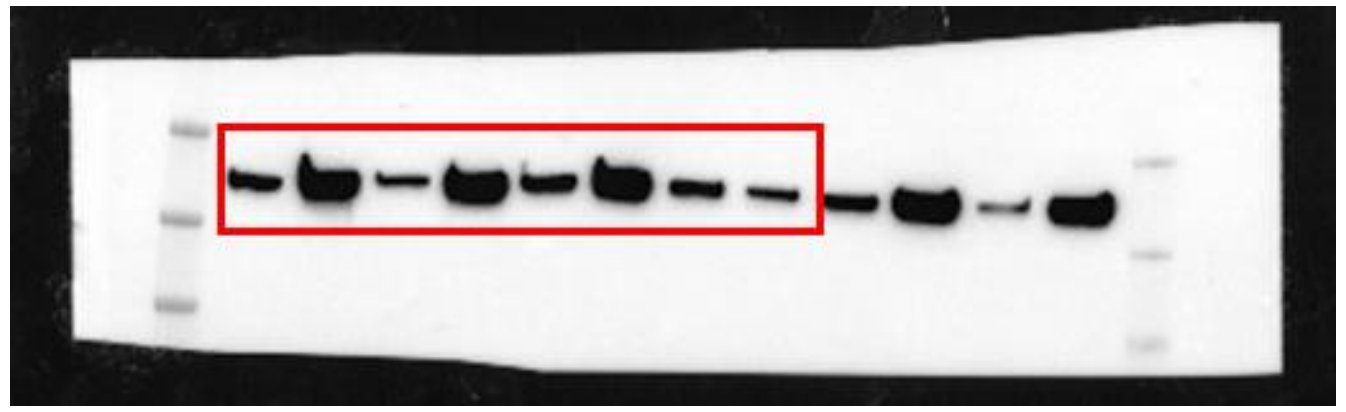

α-SMA band

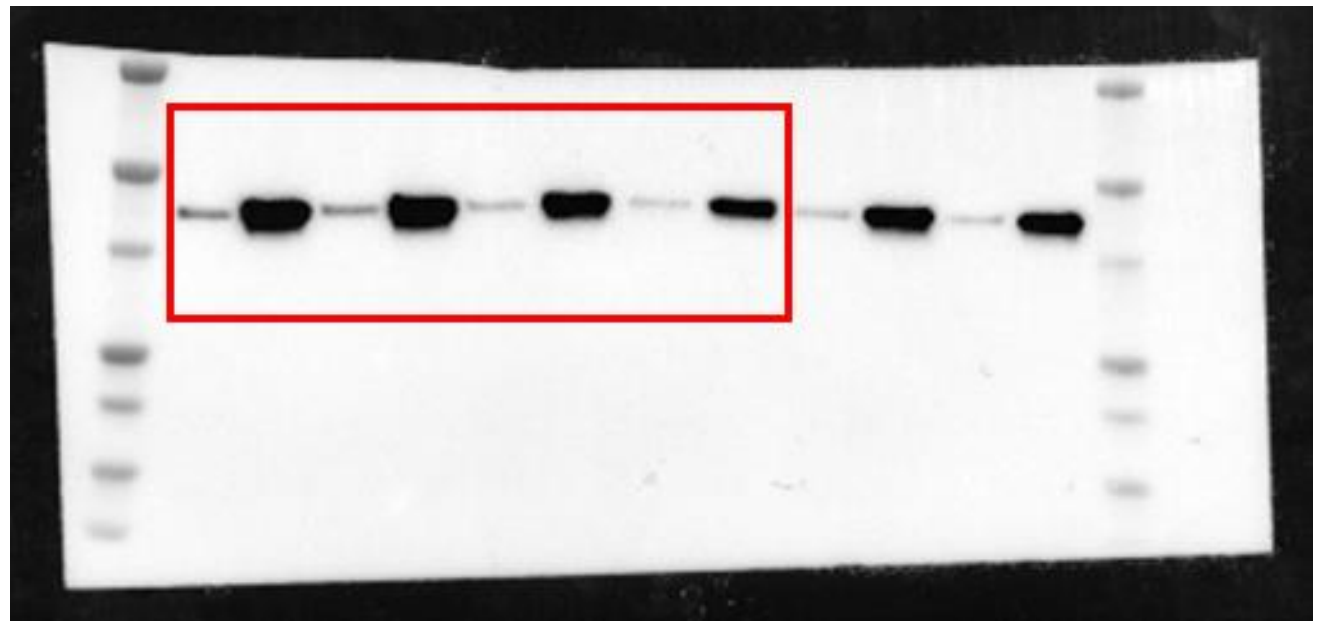

Total Protein

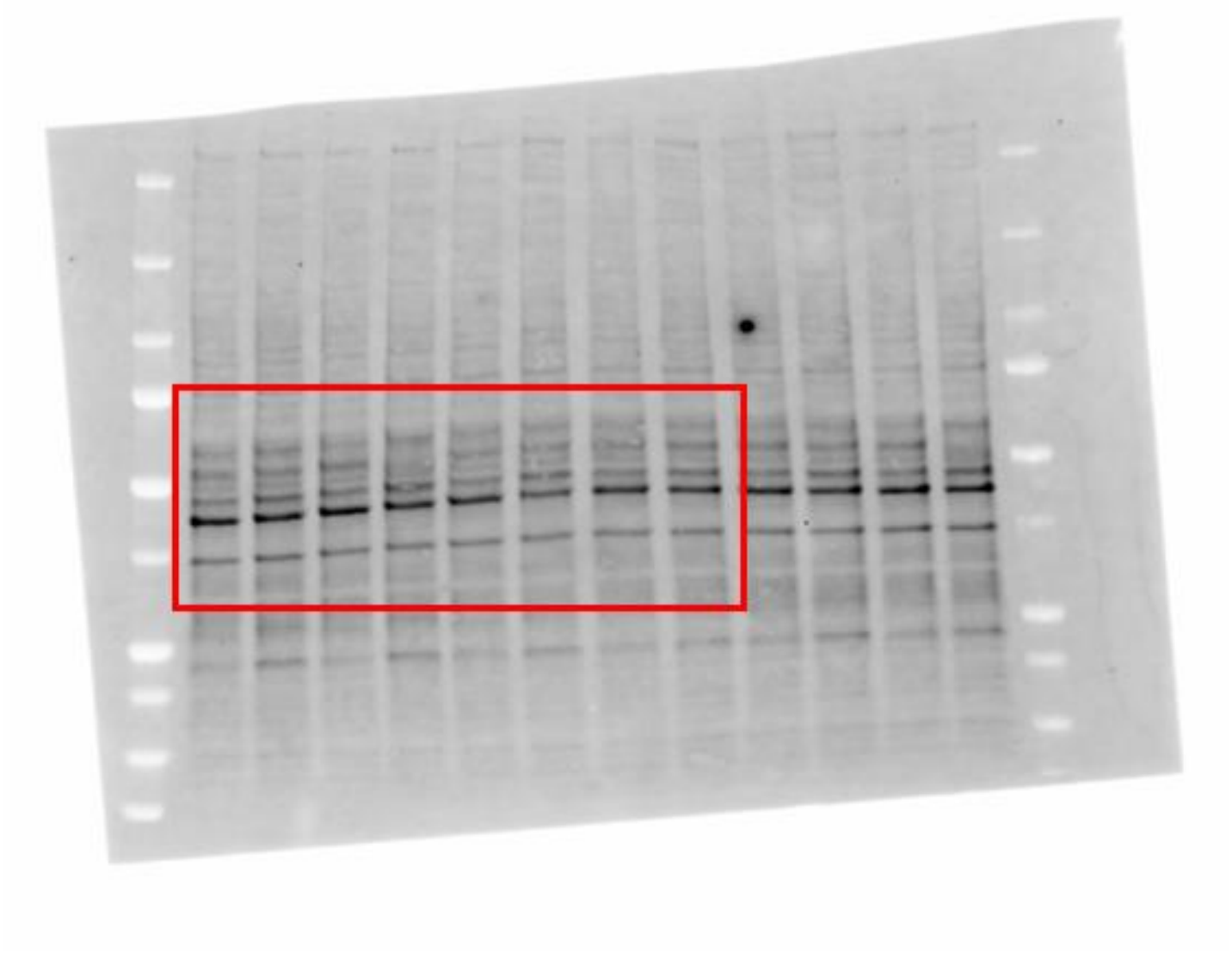

Full unedited gels for Fig. 2K

COL1A1 band

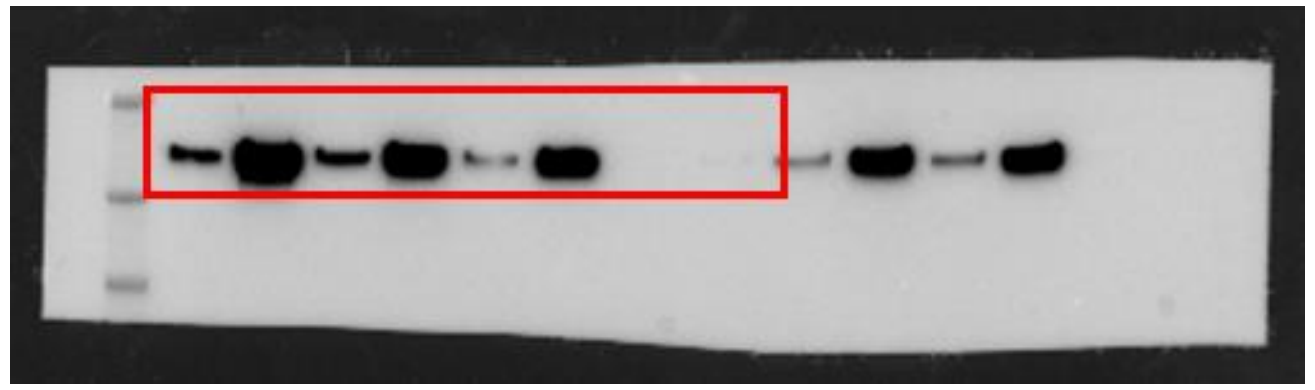

α-SMA band

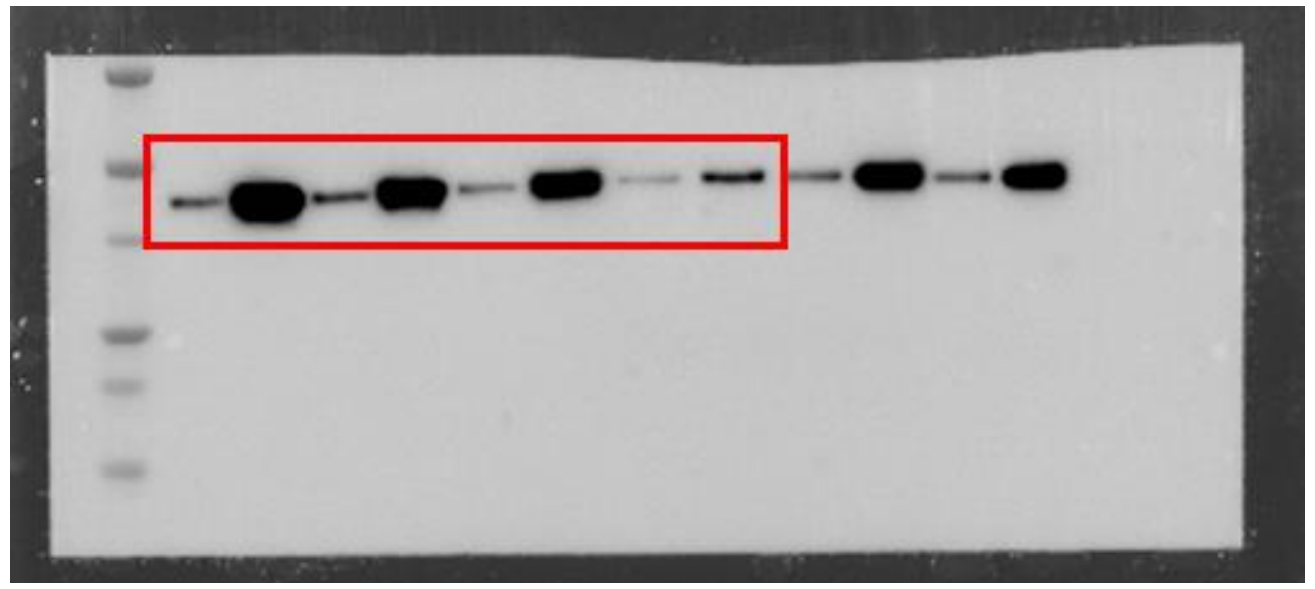

Total Protein

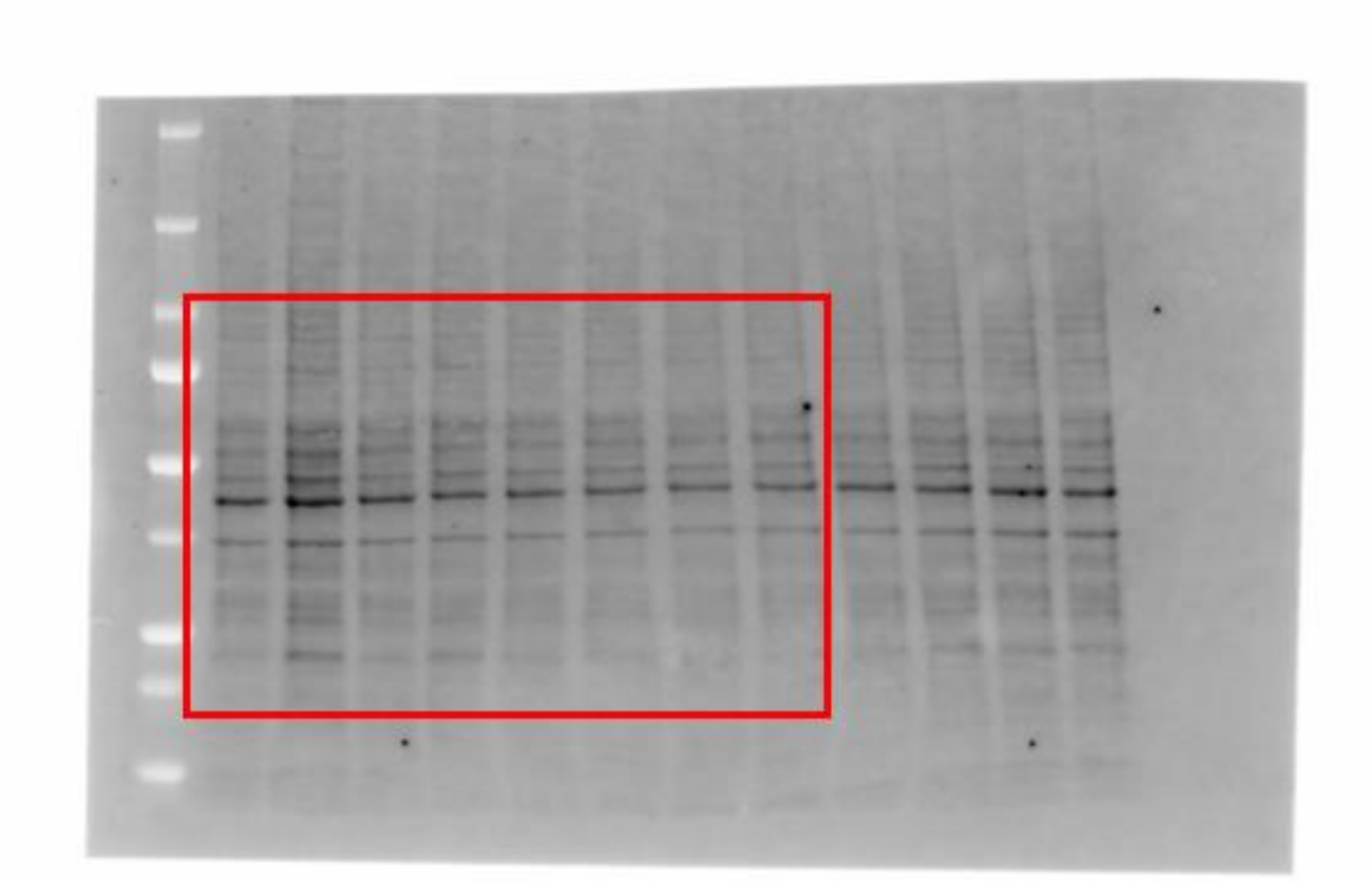

Full unedited gels for Fig. 2L

COL1A1 band

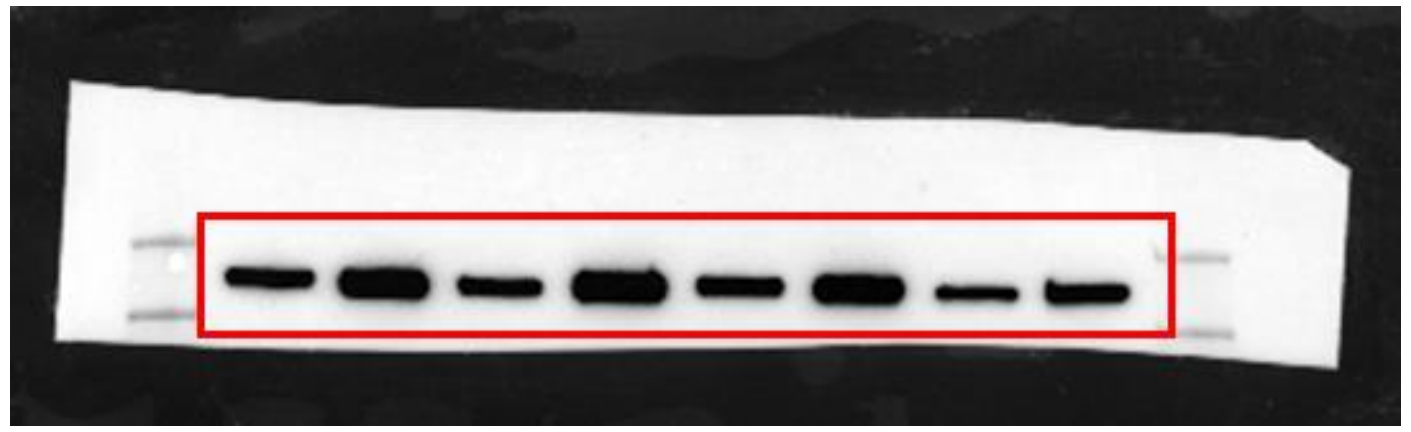

α-SMA band

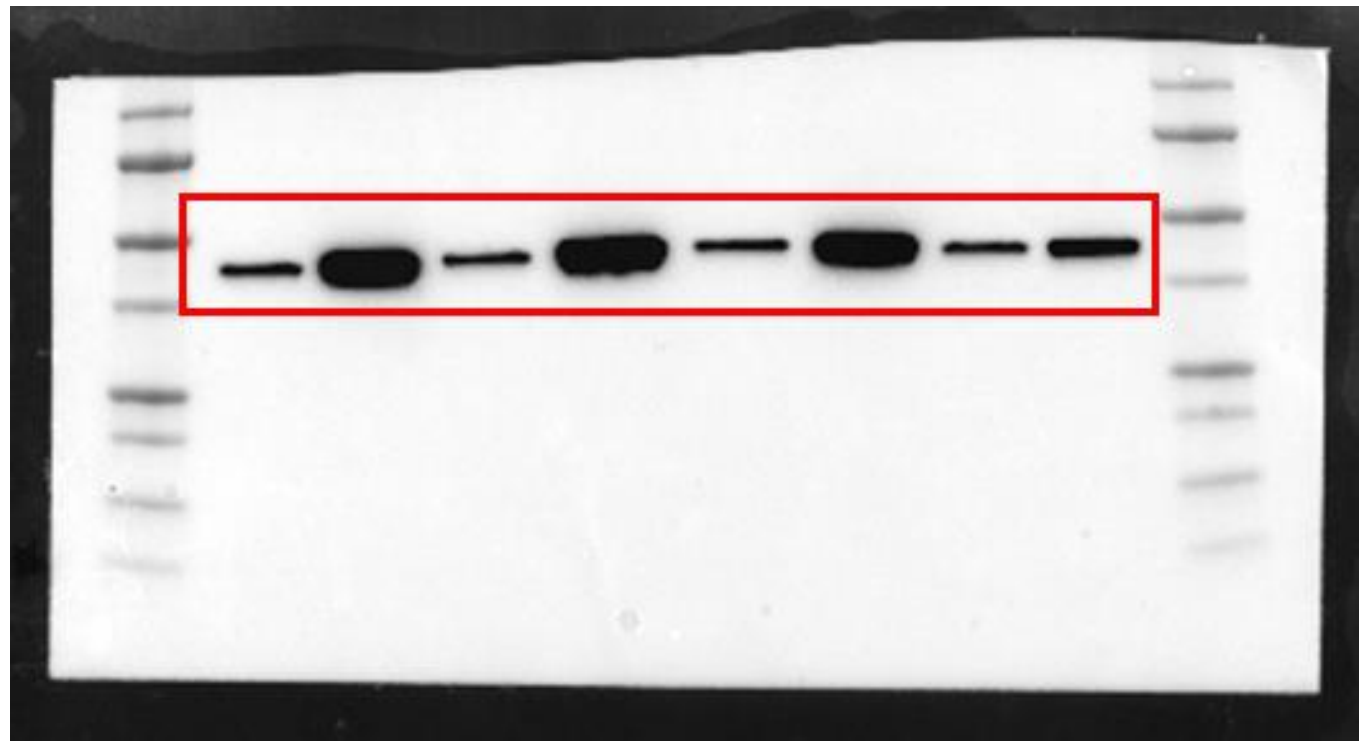

GPT2 band

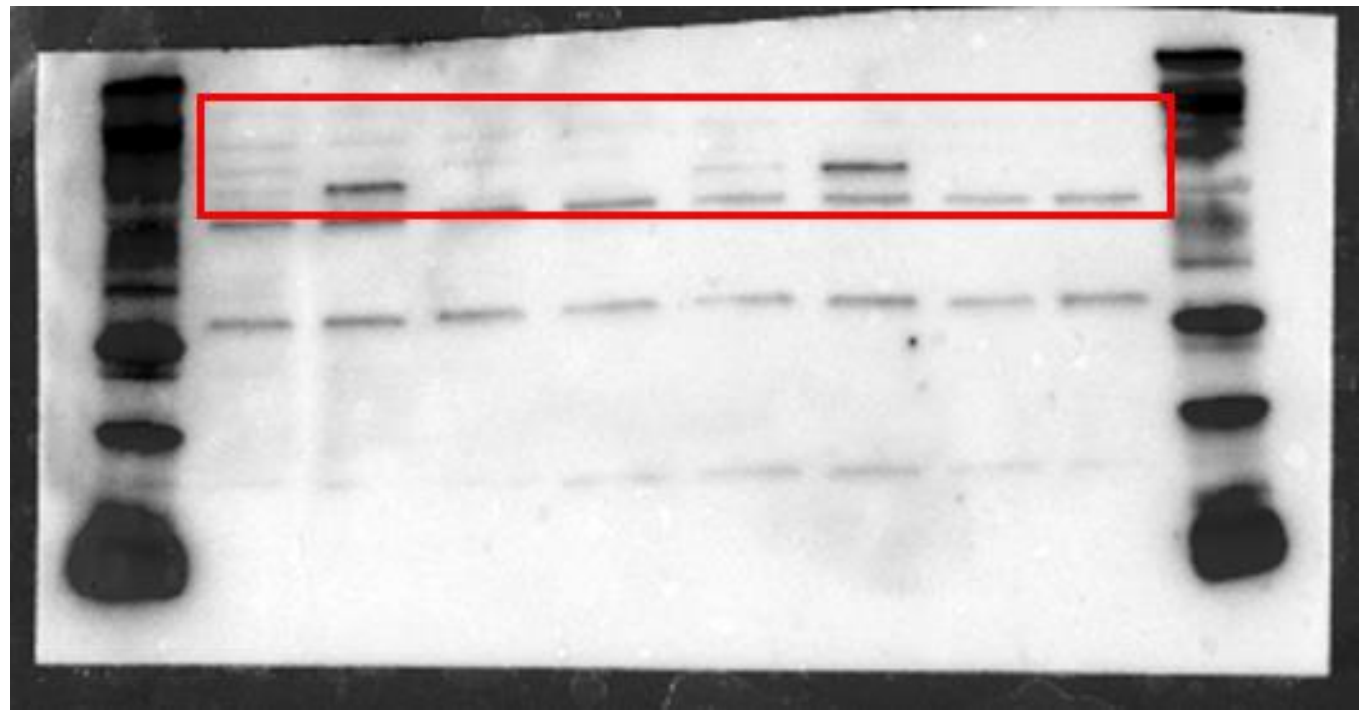

Total Protein

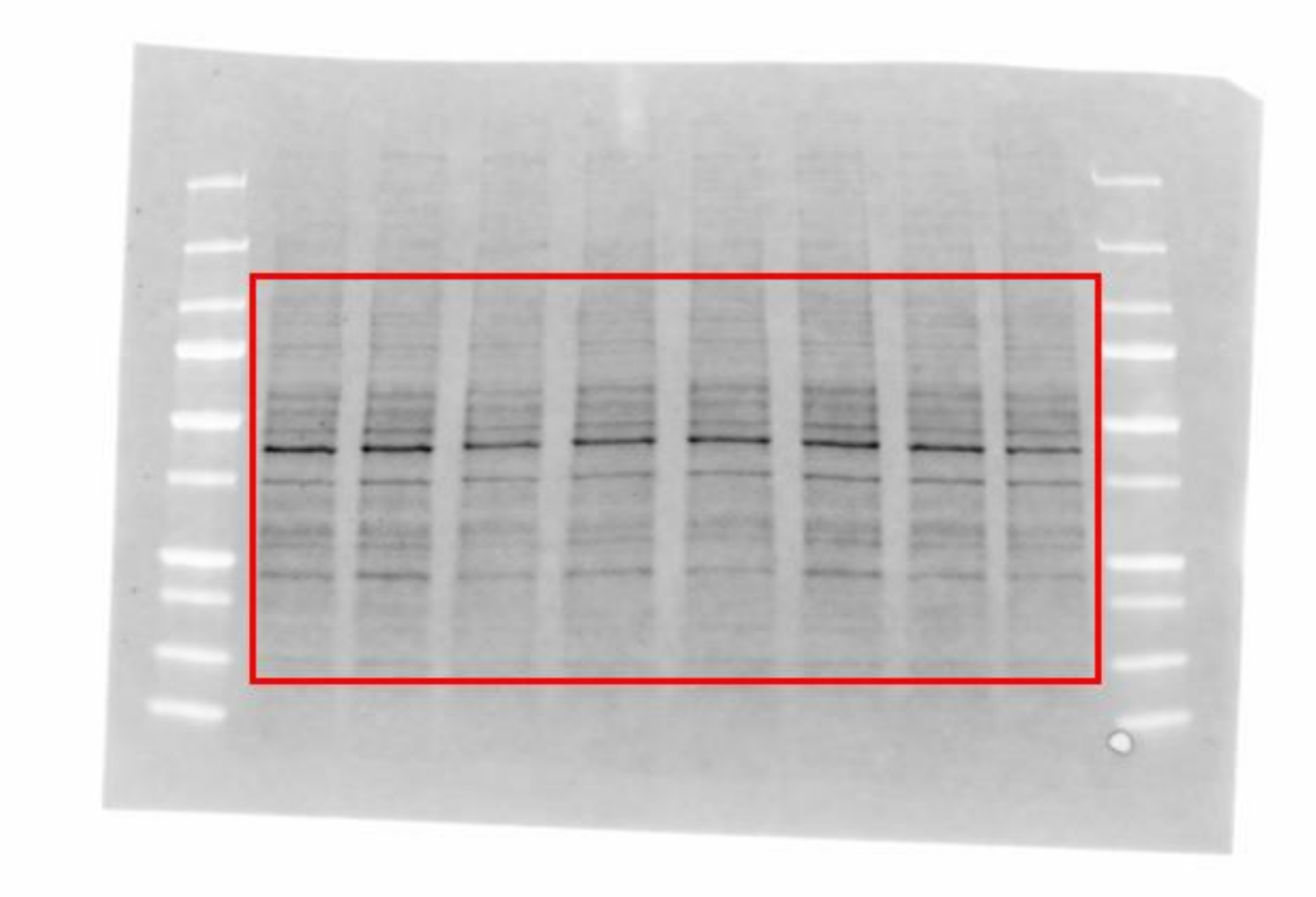

Full unedited gels for Fig. 3C

COL1A1 band

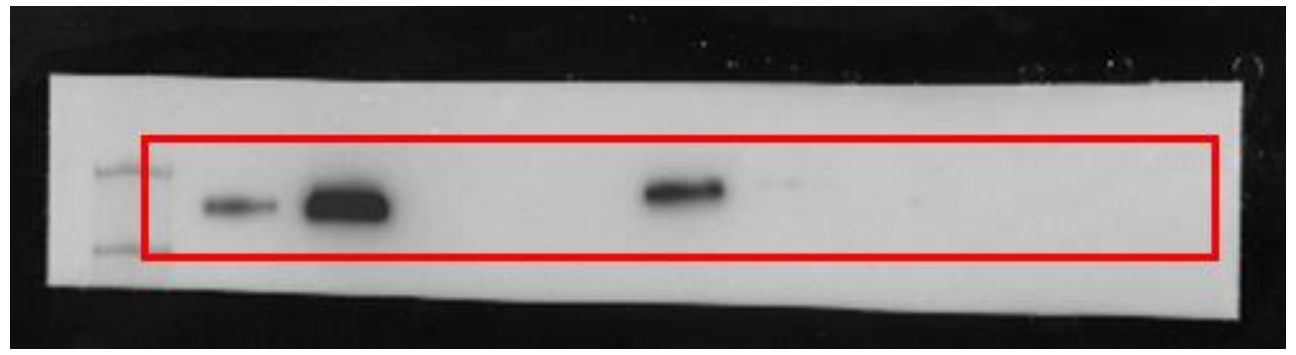

α-SMA band

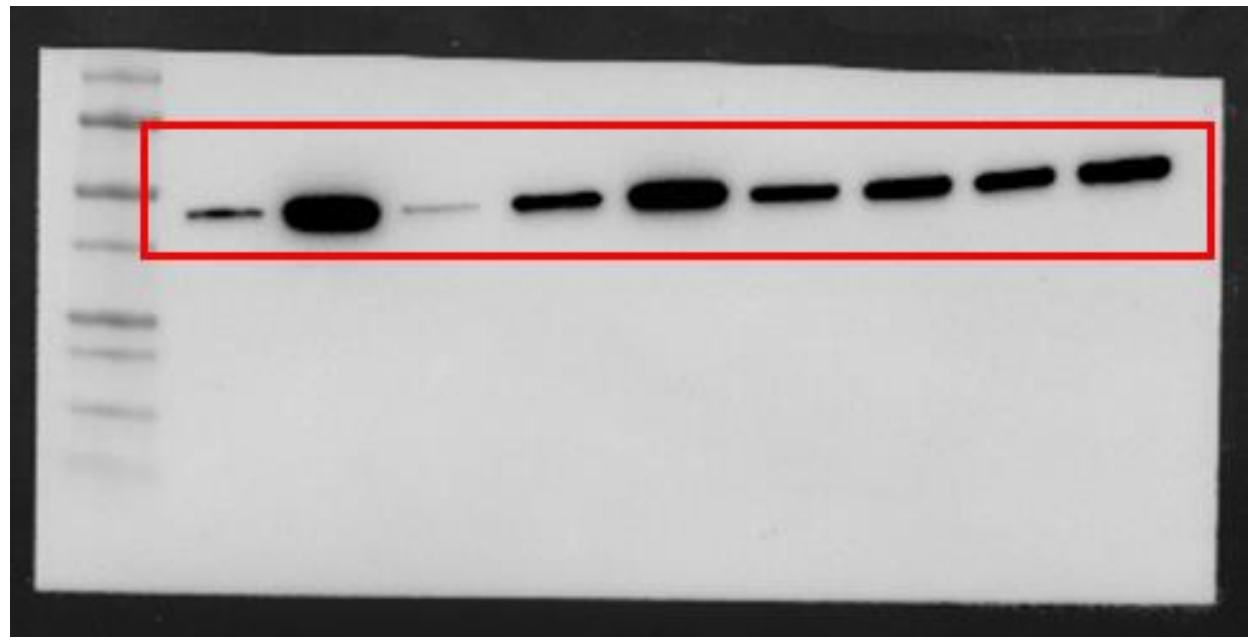

Total Protein

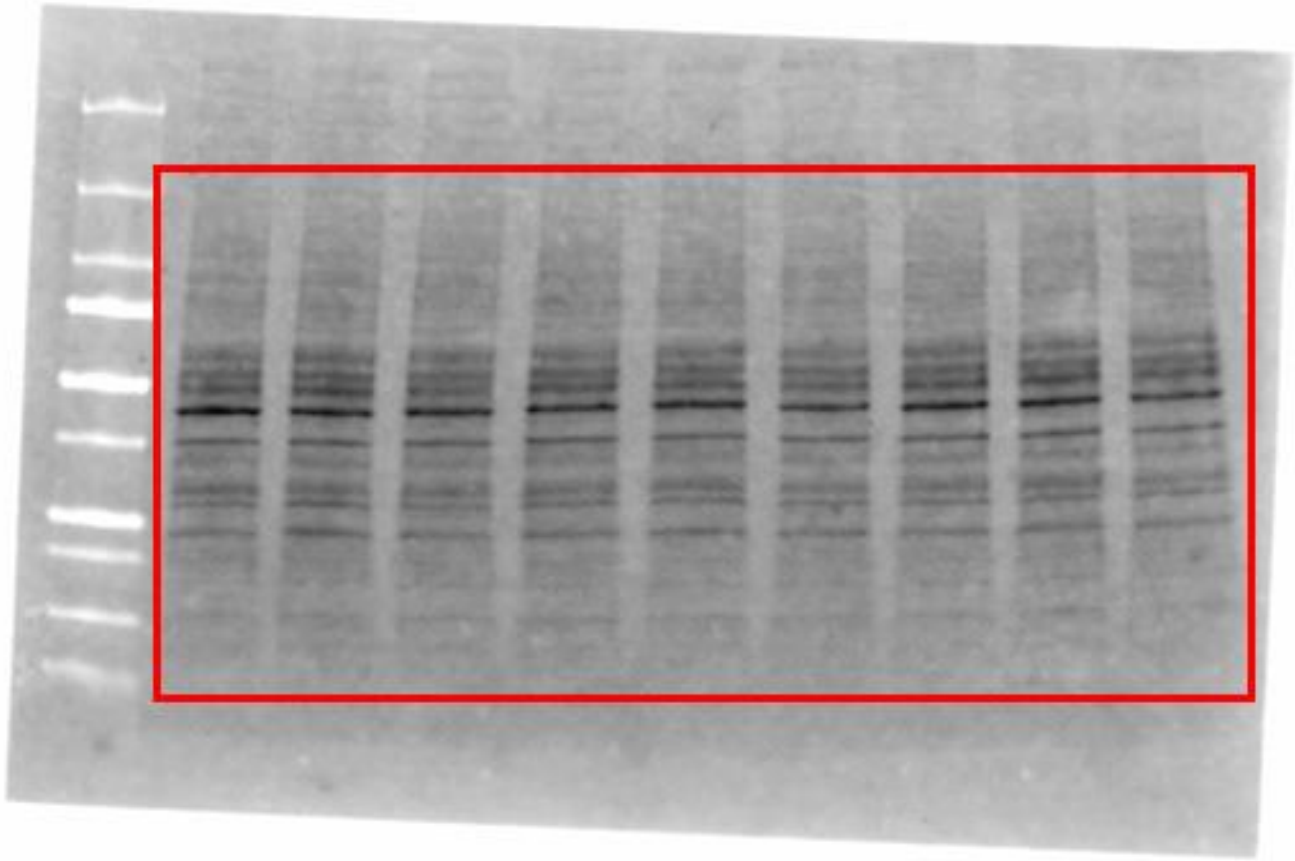

Full unedited gels for Fig. 3D

COL1A1 band

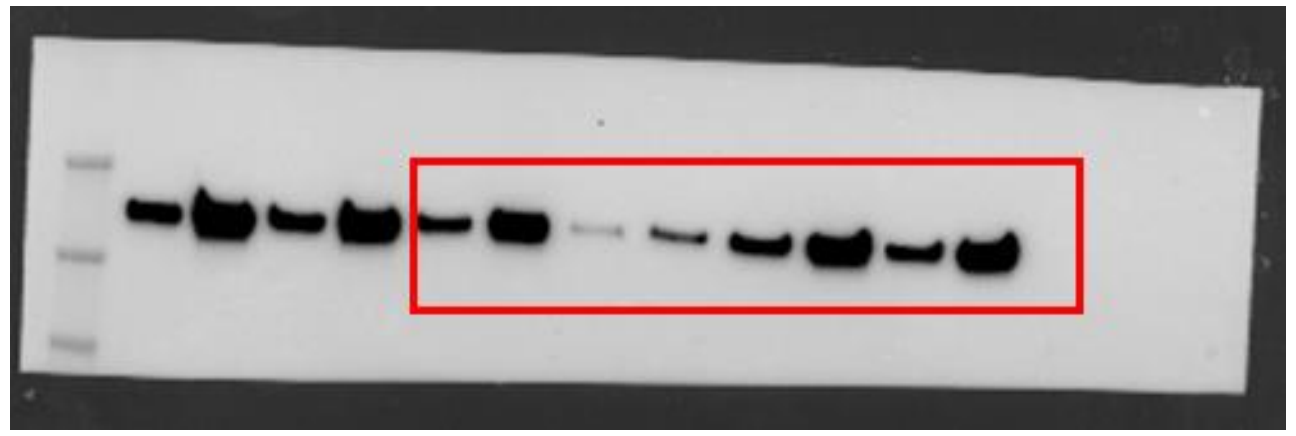

α-SMA band

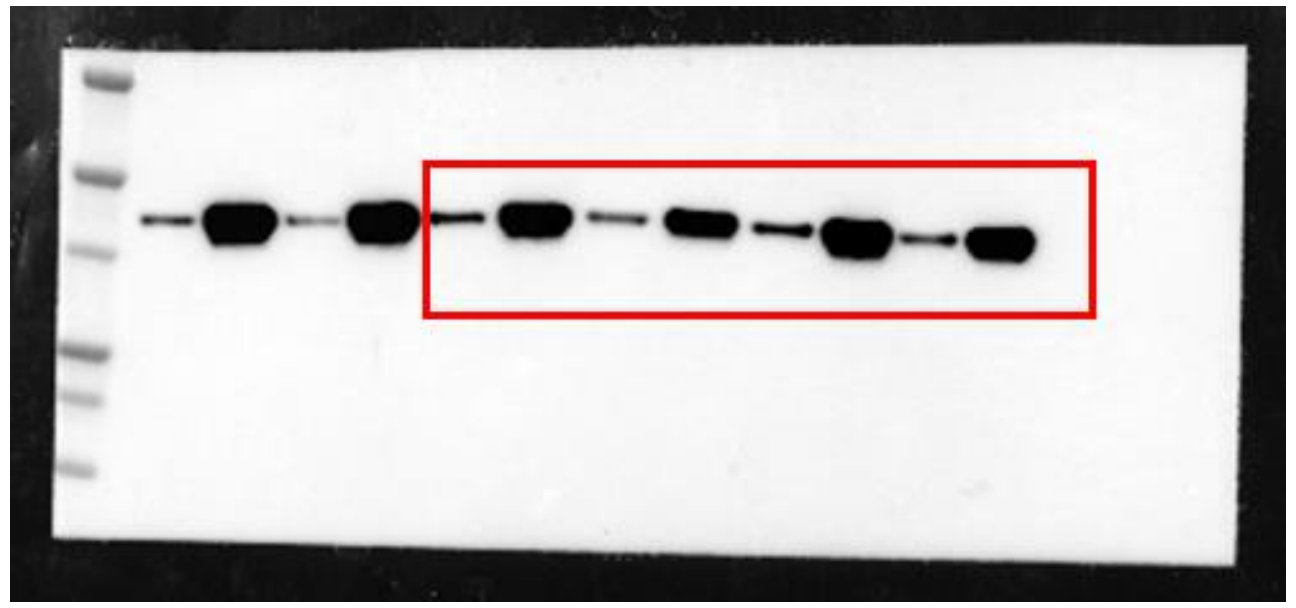

Total Protein

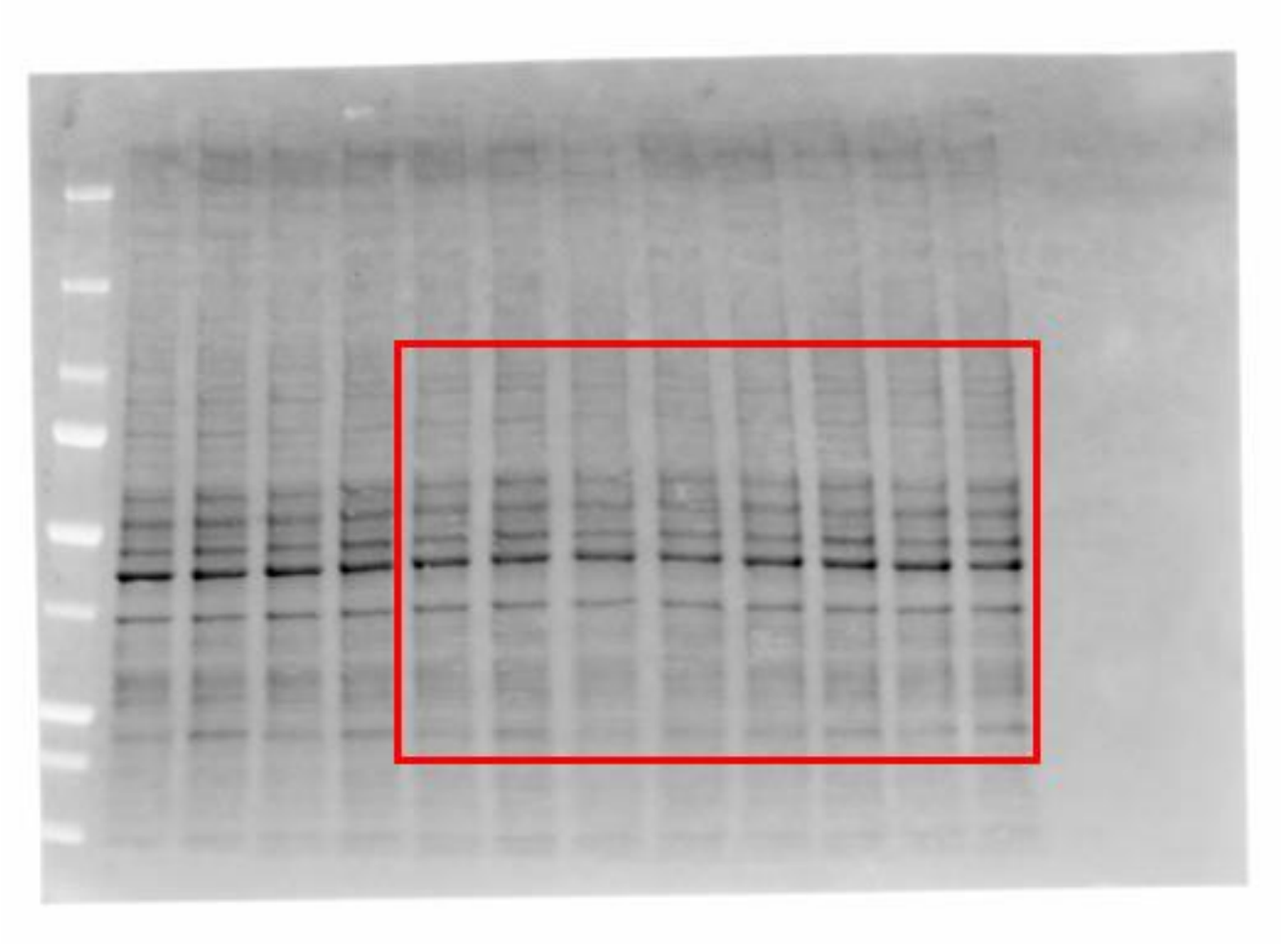

Full unedited gels for Fig. 3E

COL1A1 band

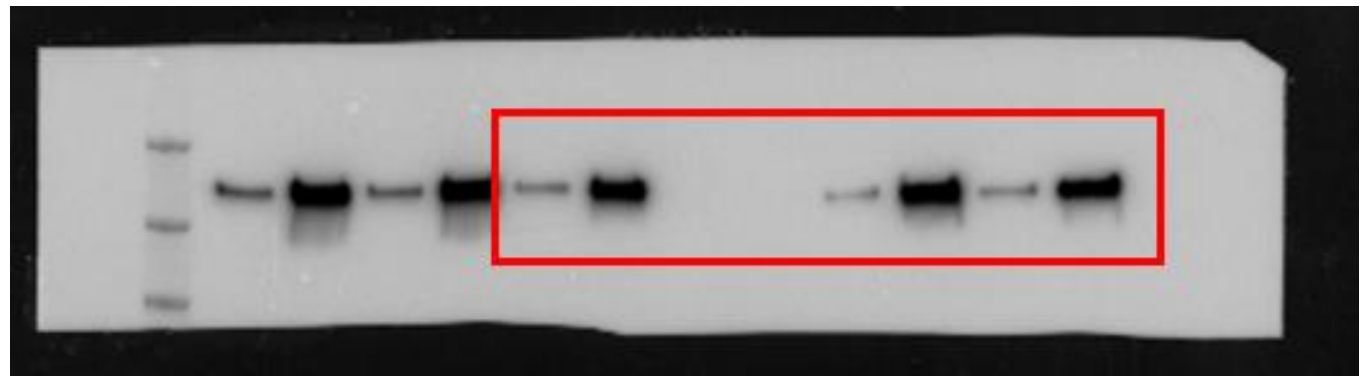

α-SMA band

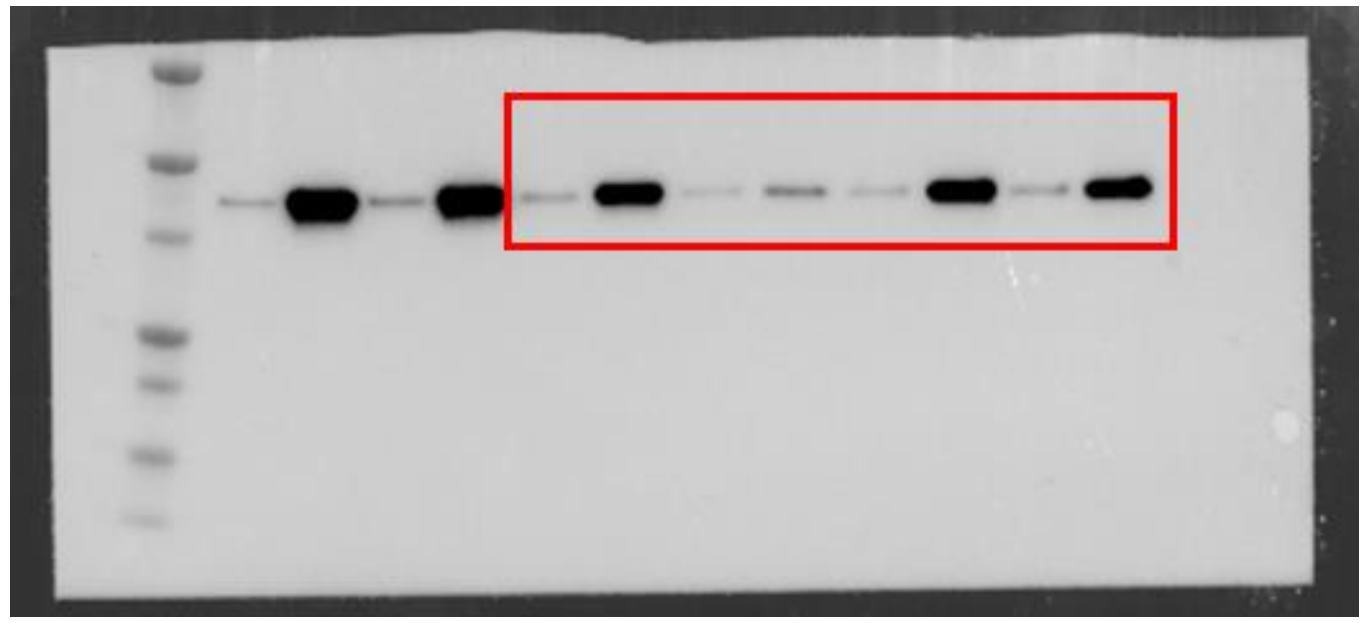

Total Protein

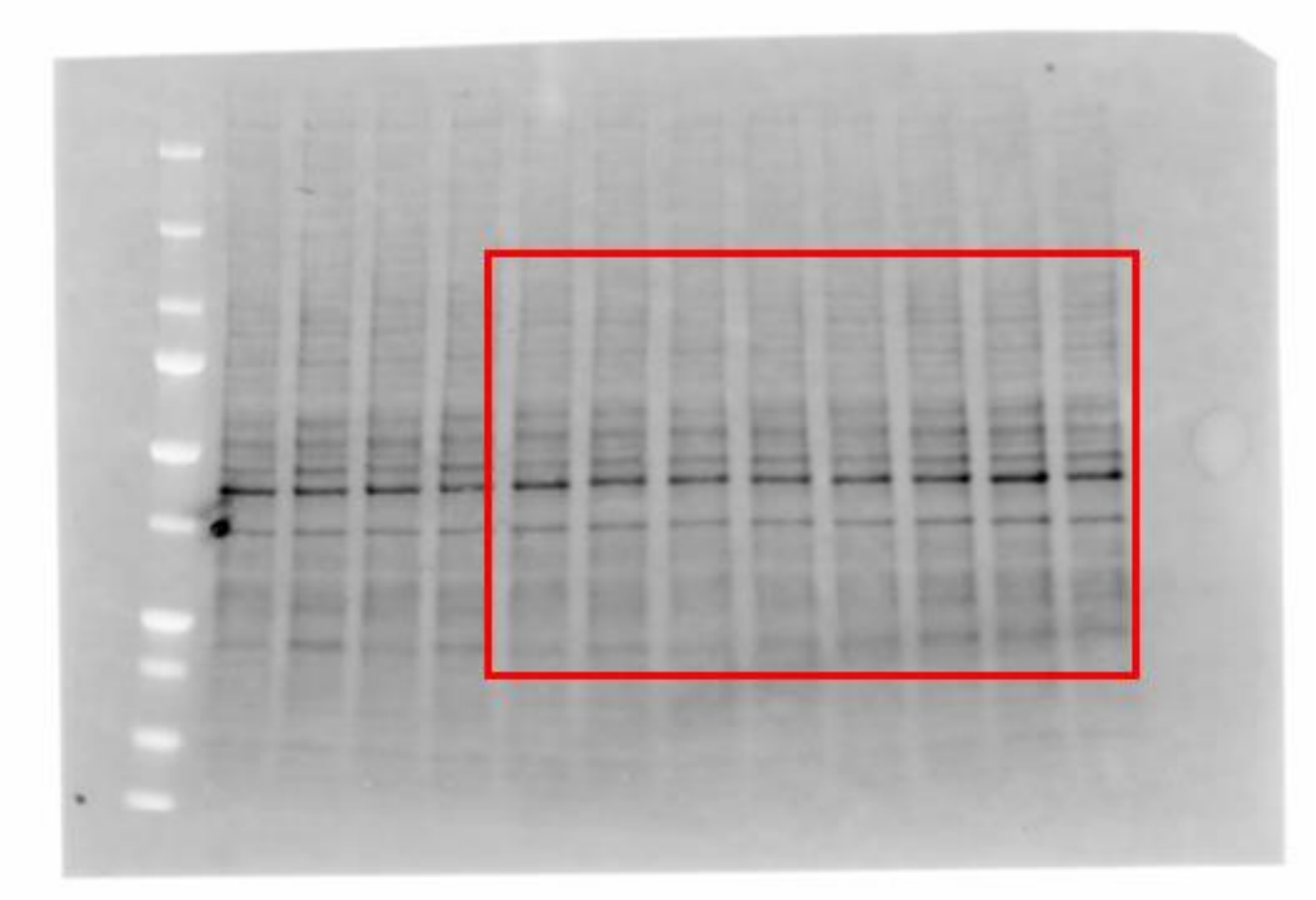

Full unedited gels for Fig. 3F

COL1A1 band

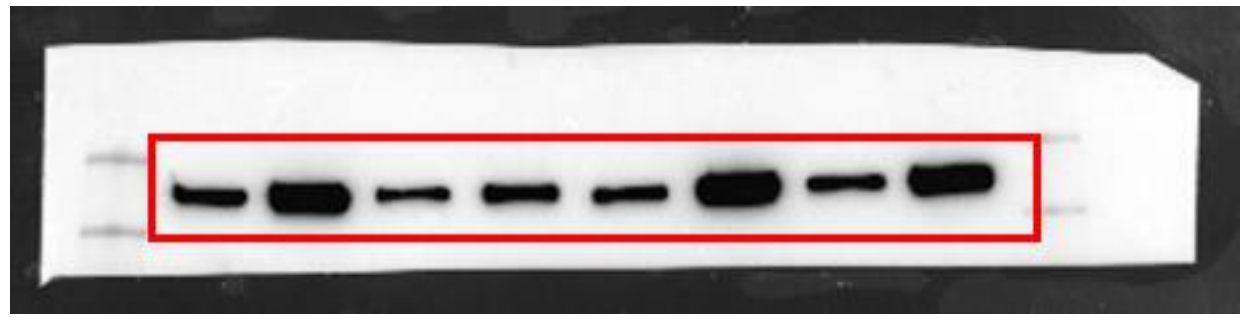

α-SMA band

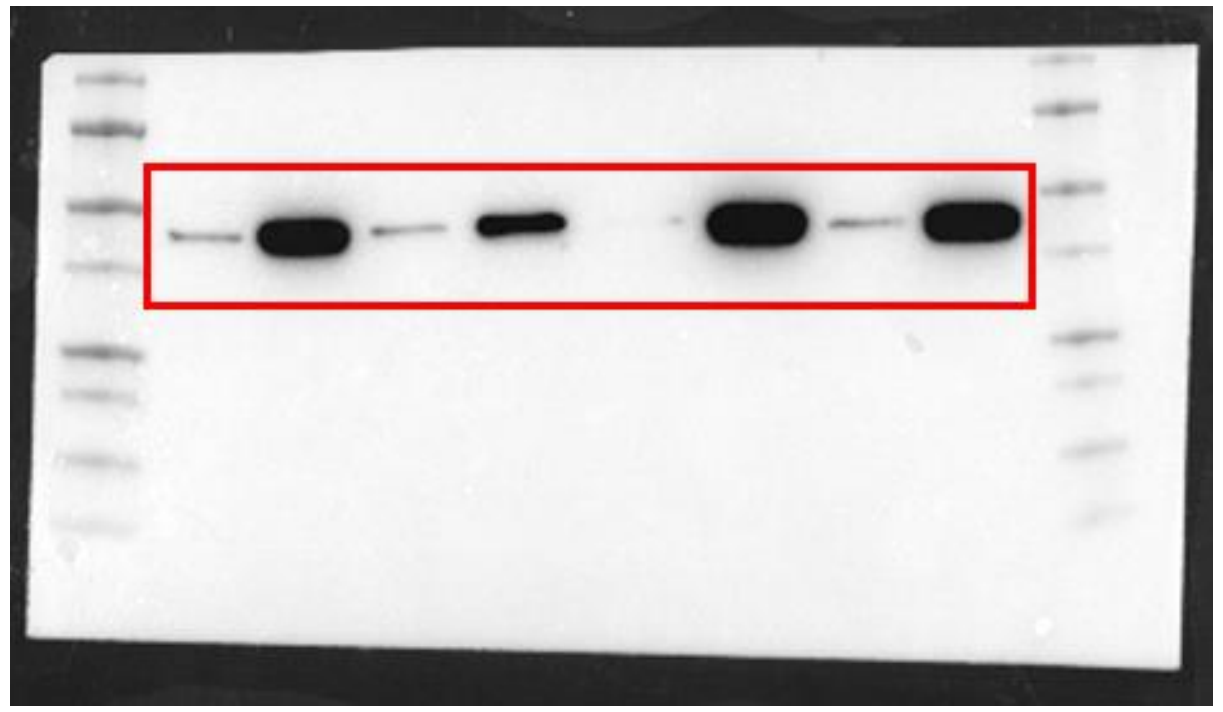

GPT2 band

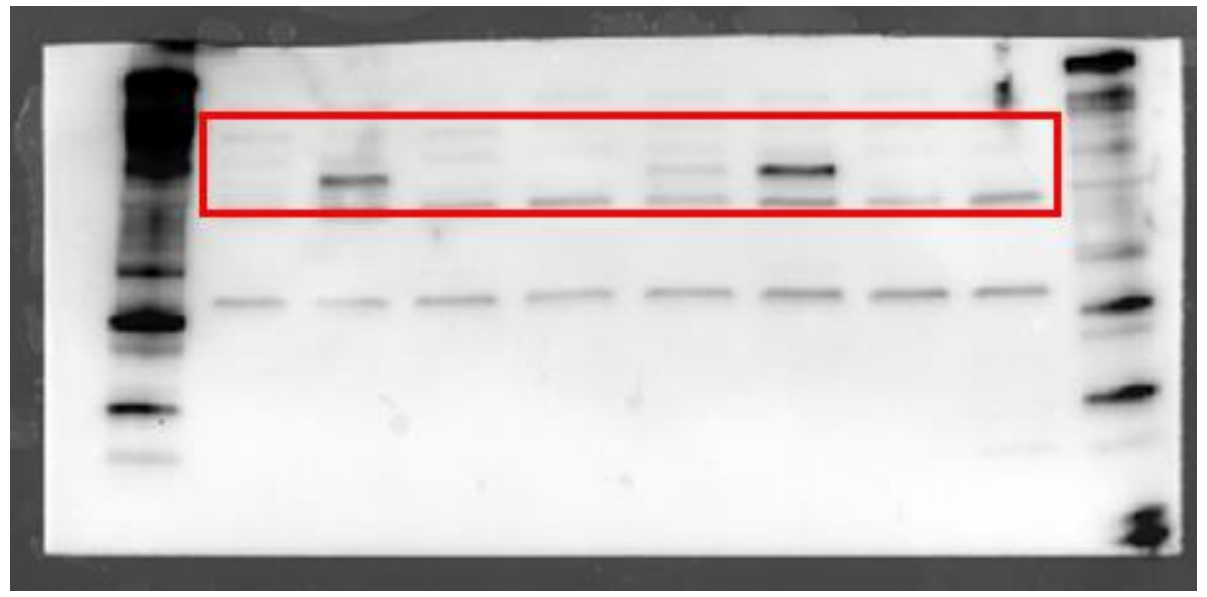

Total Protein

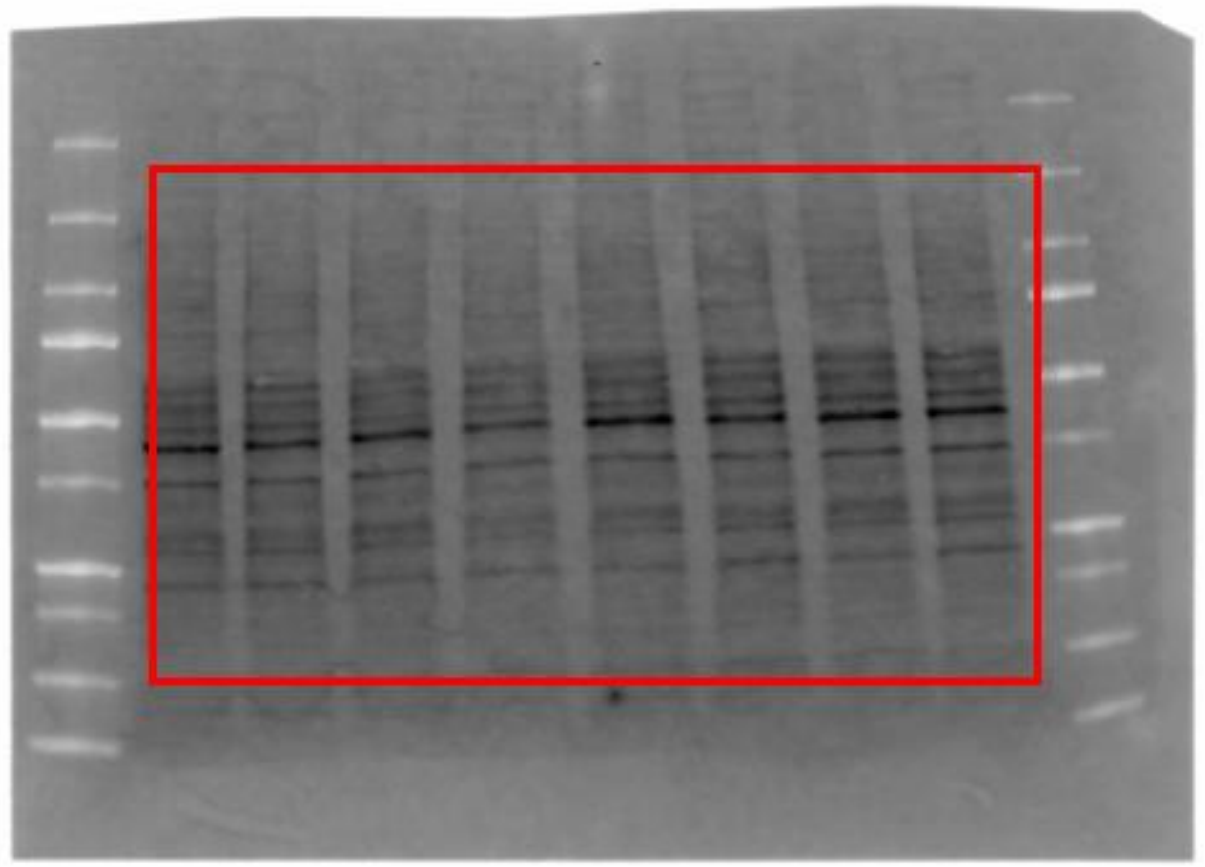

Full unedited gels for Fig. 4C

SLC38A2 band

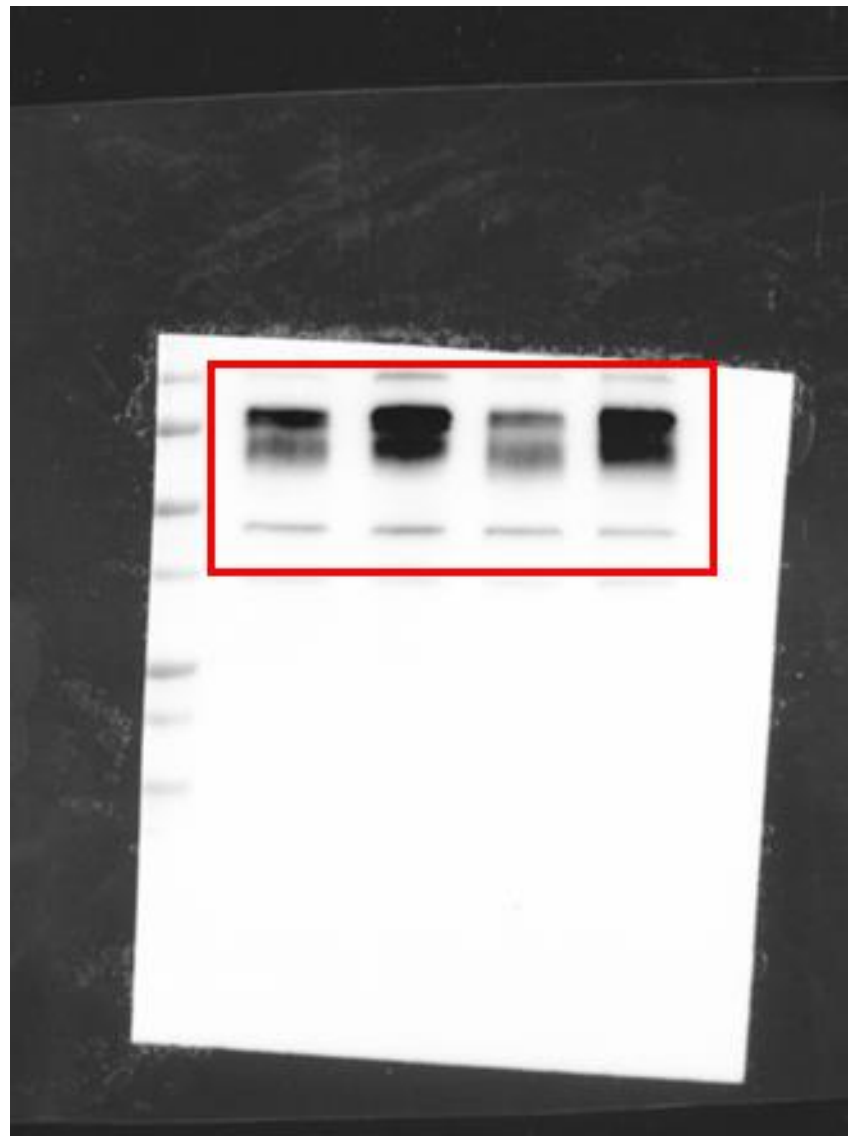

Total Protein

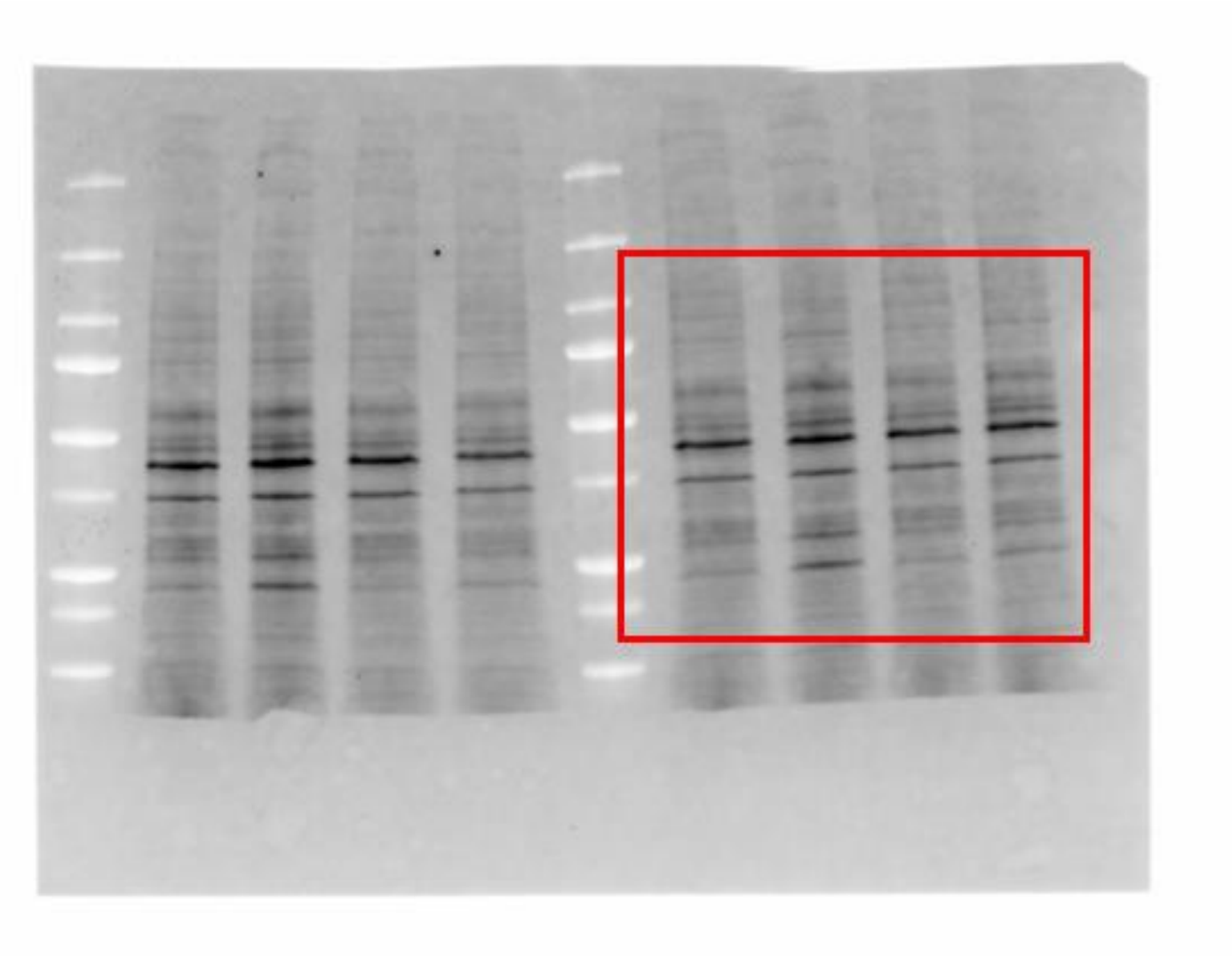

Full unedited gels for Fig. 4D

SLC38A2 band

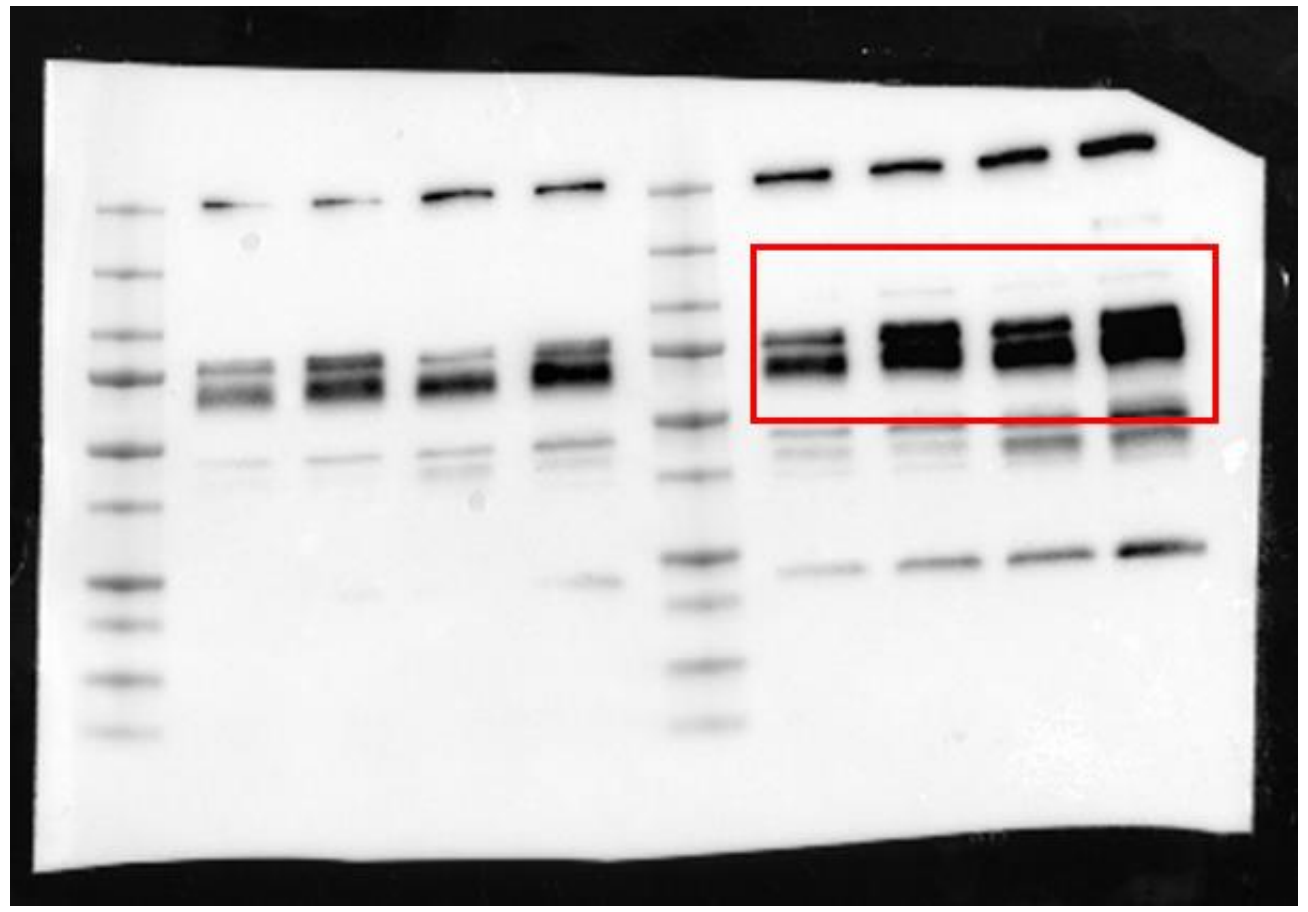

Total Protein

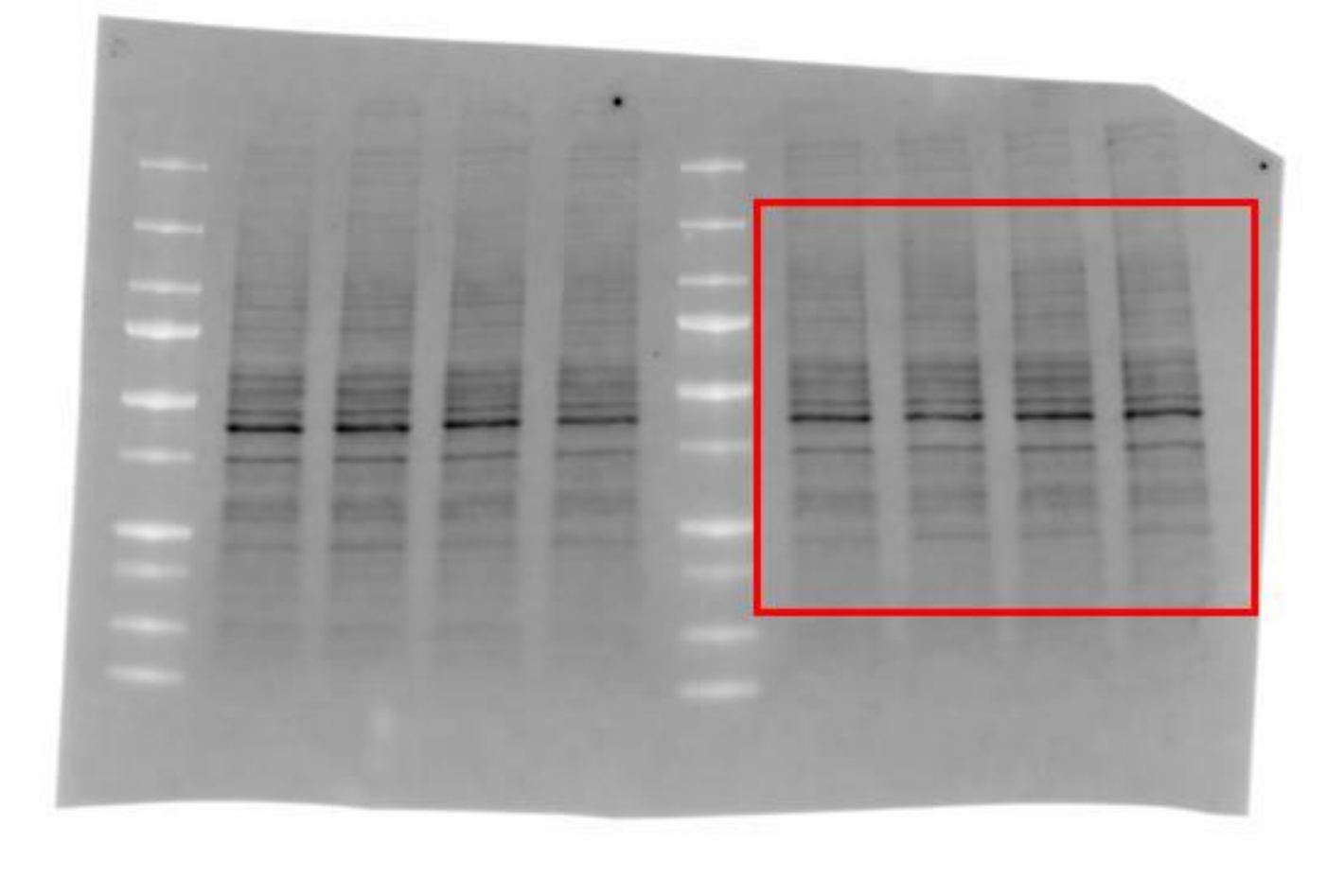

Full unedited gels for Fig. 4E

SLC38A2 band

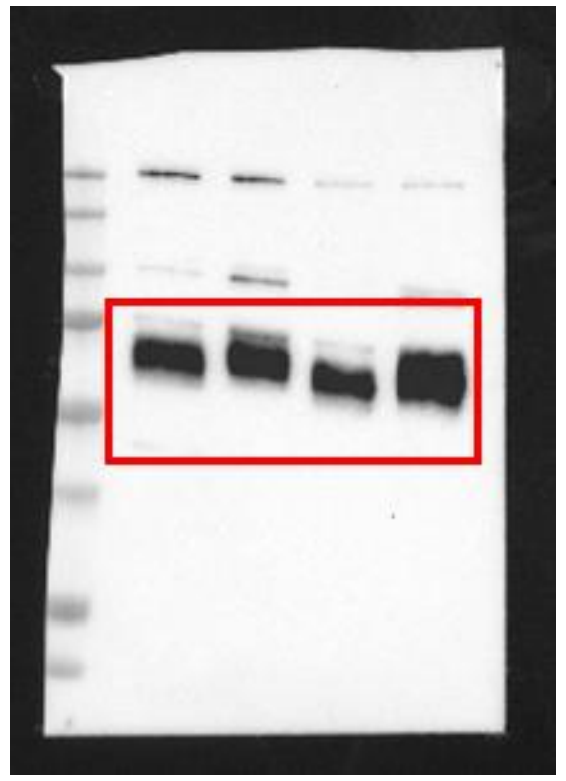

GPT2 band

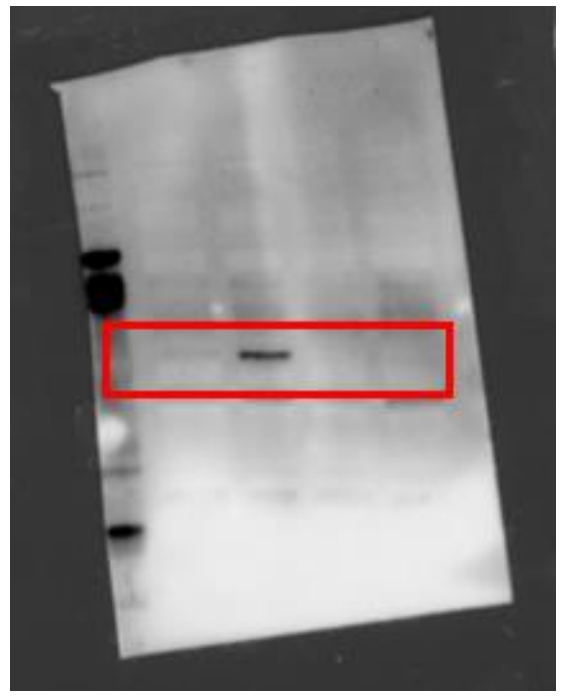

Total Protein

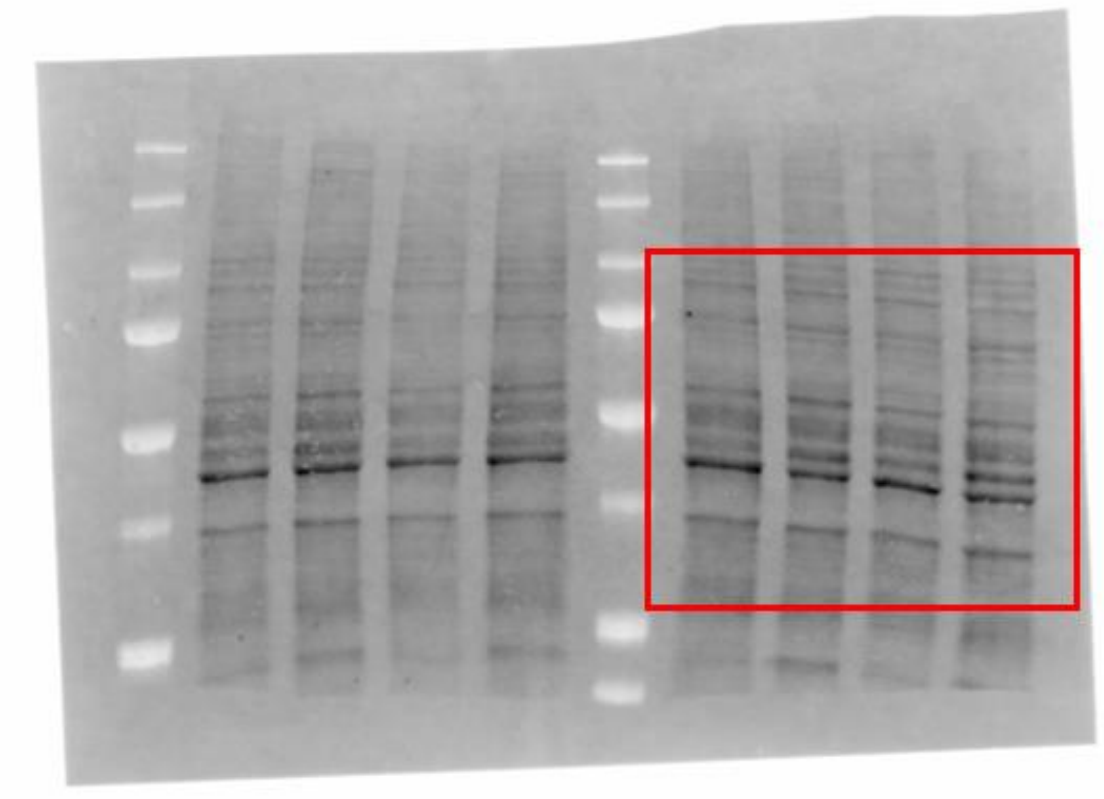

Full unedited gels for Fig. 4G

SLC38A2 band

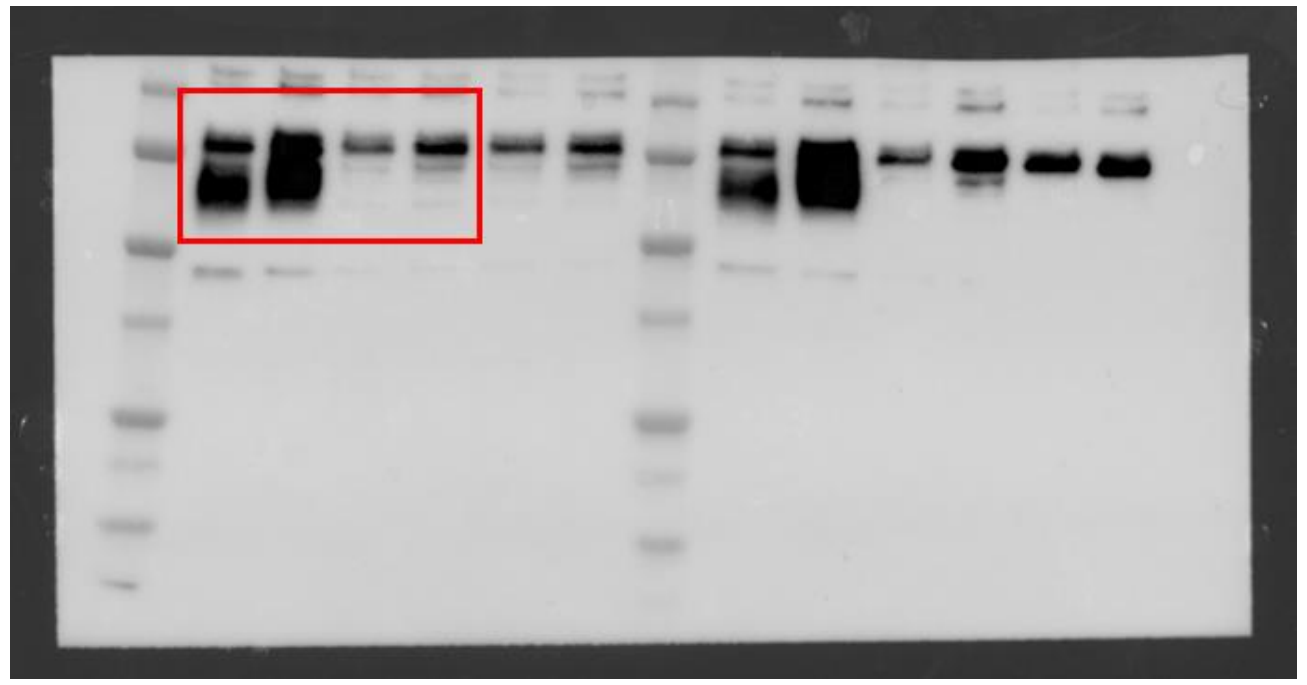

GPT2 band

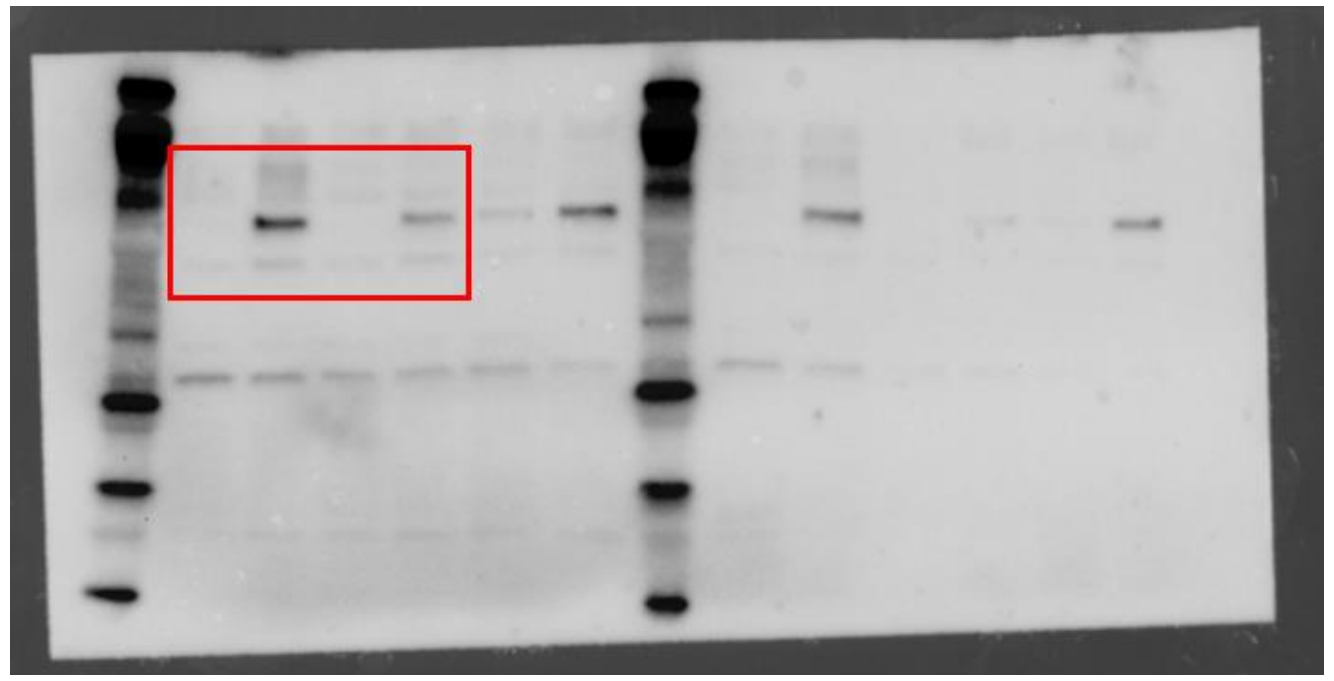

Total Protein

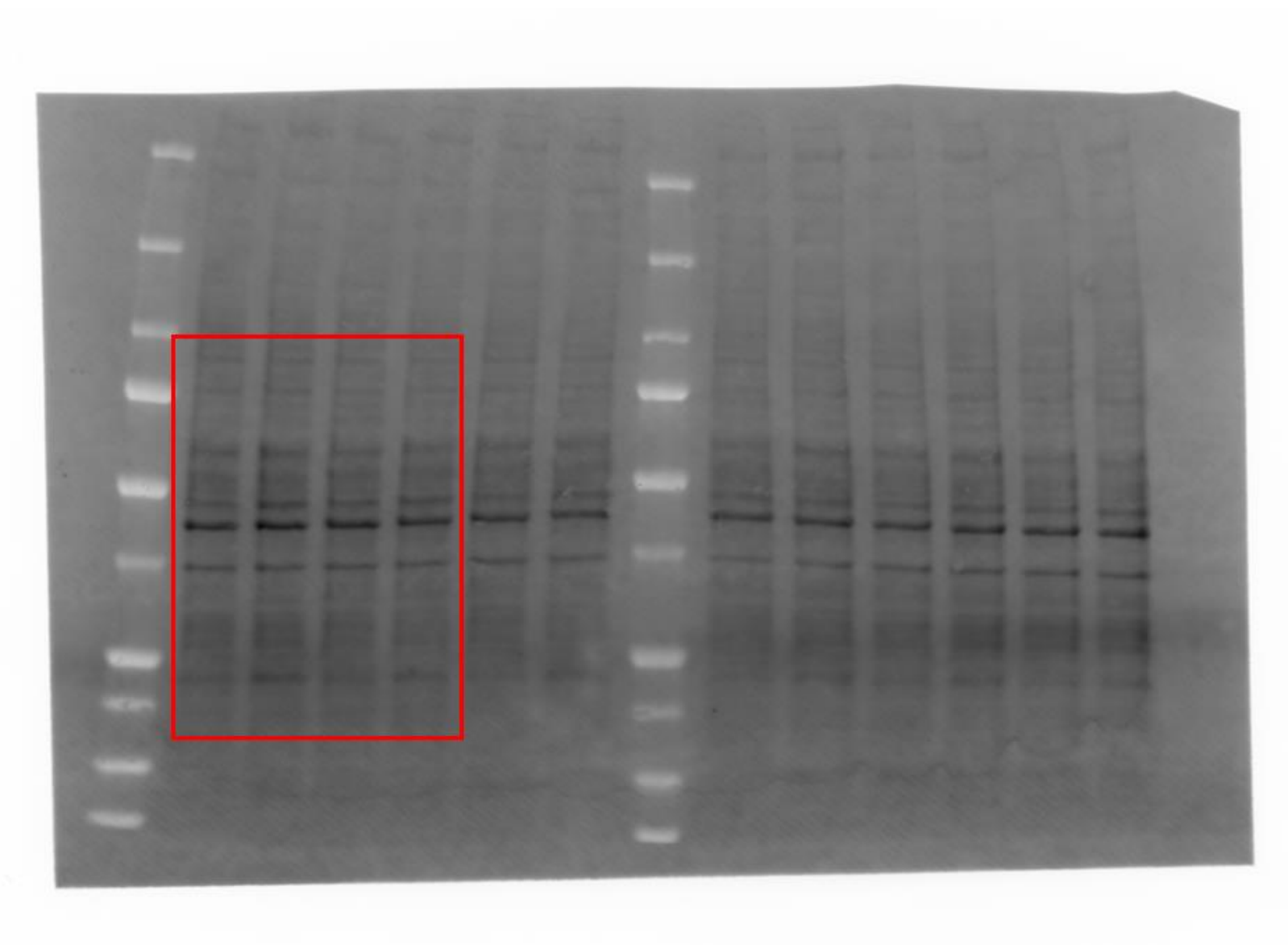

Full unedited gels for Fig. 4H

COL1A1 band

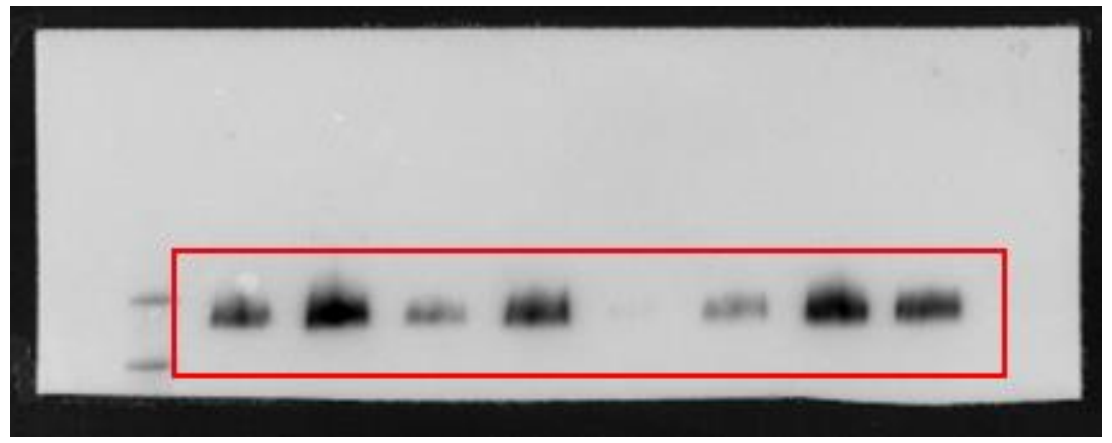

Total Protein

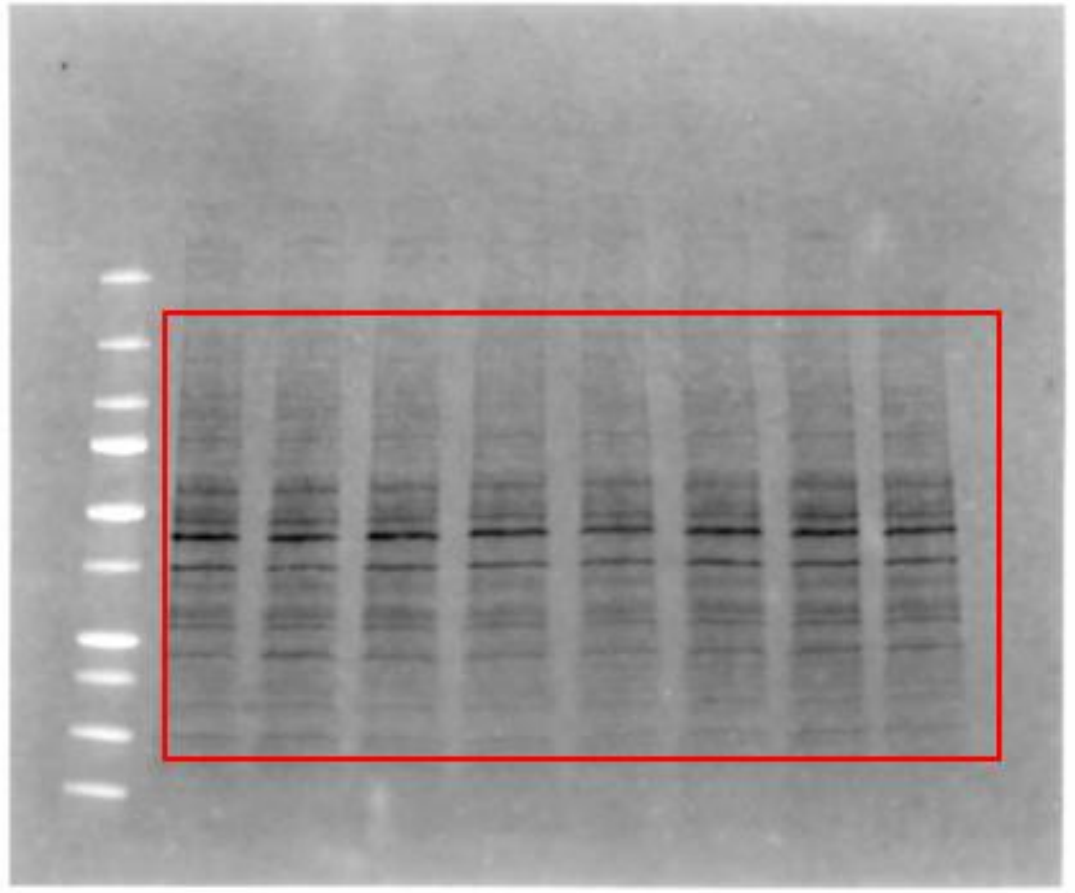

SLC38A2 band

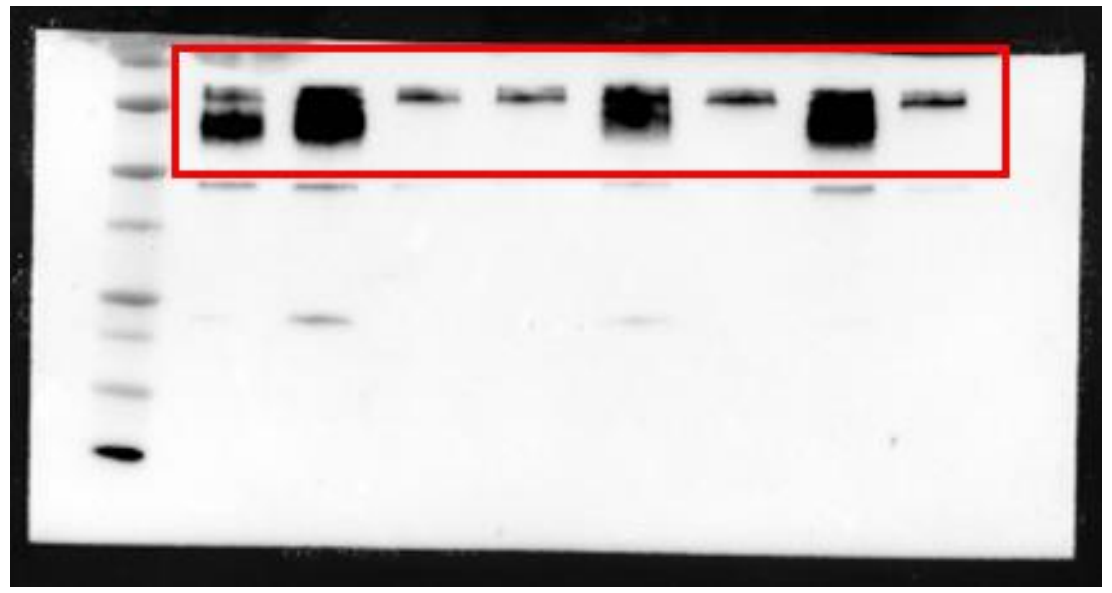

α-SMA band

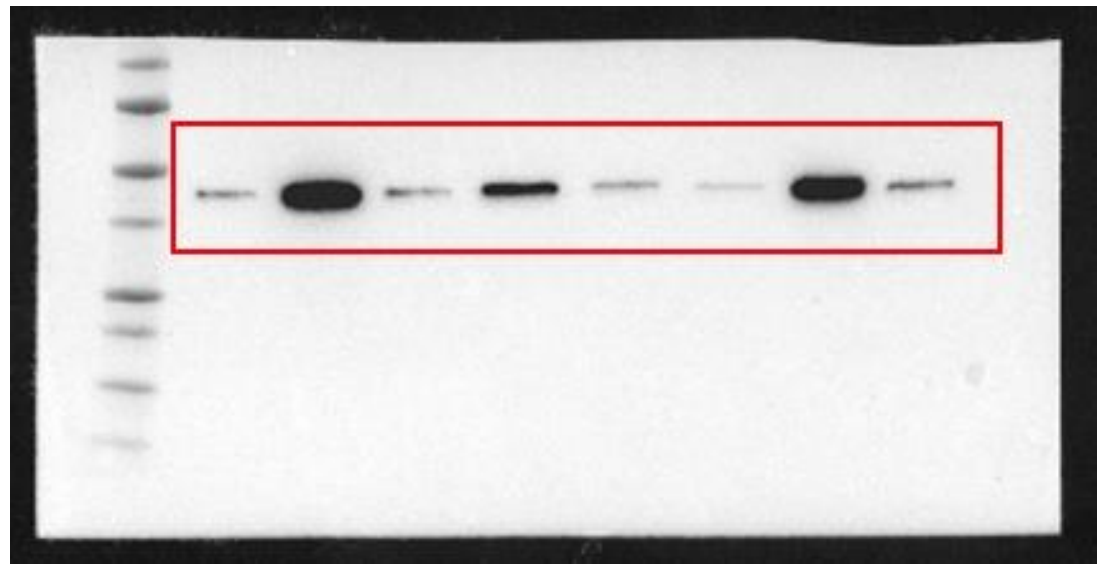

Full unedited gels for Fig. 4I

COL1A1 band

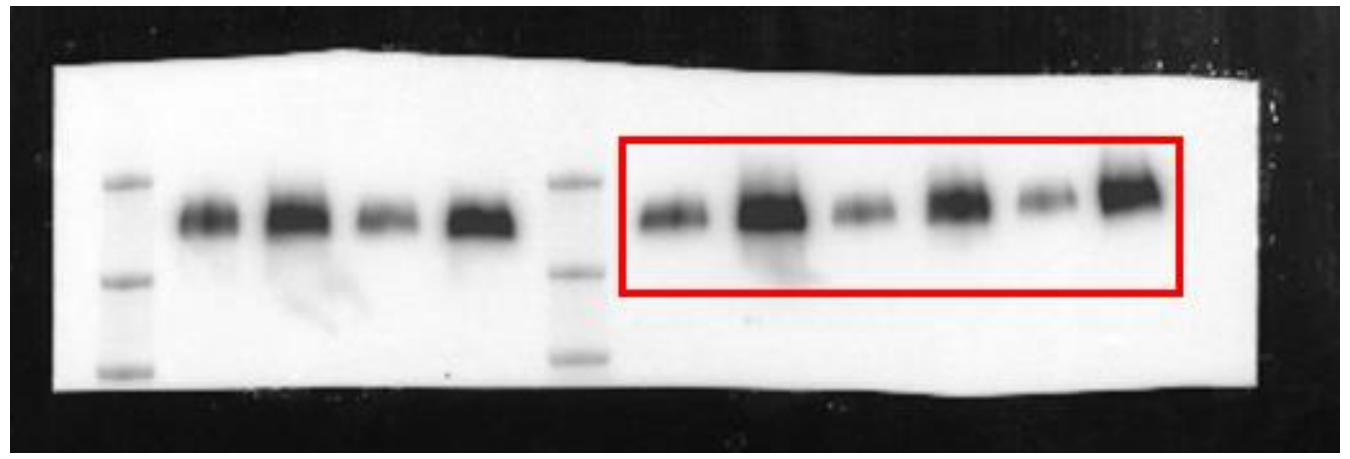

SLC38A2 band

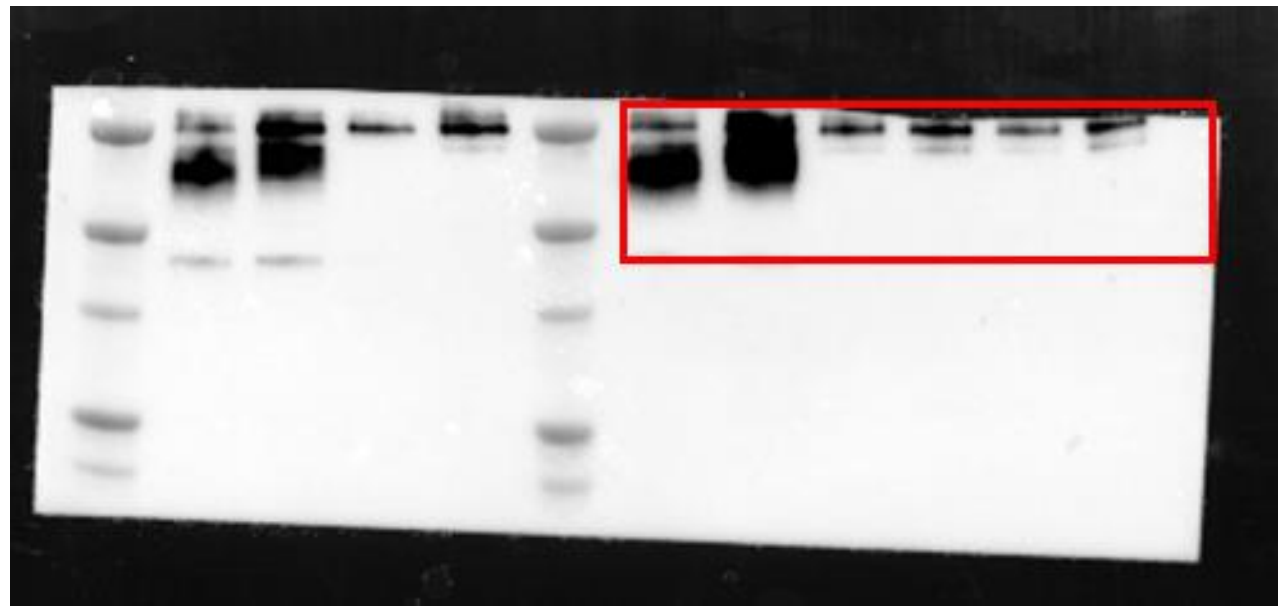

α-SMA band

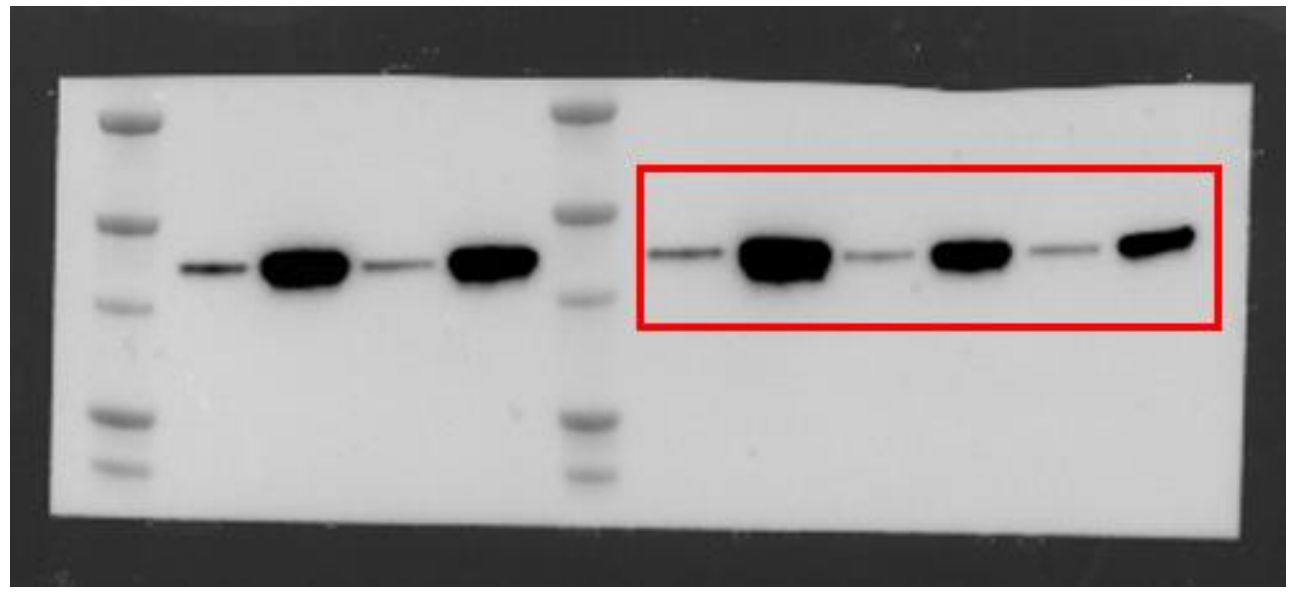

Total Protein

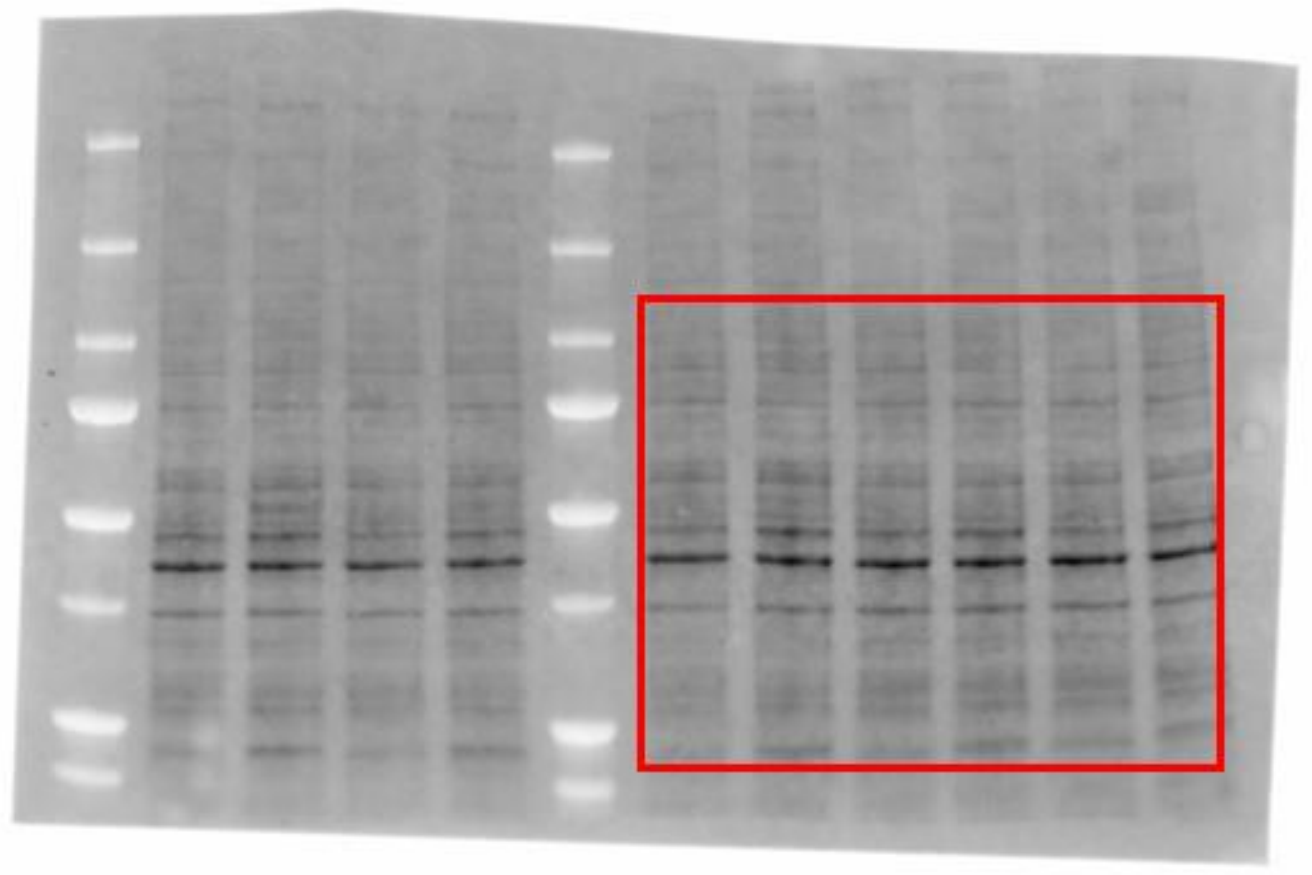

Full unedited gels for Fig. 6J

COL1A1 band

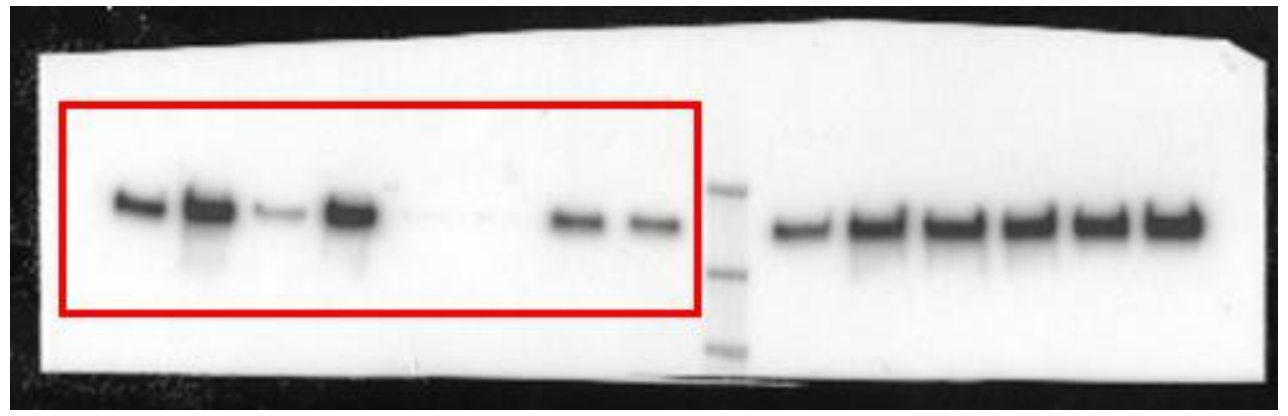

α-SMA band

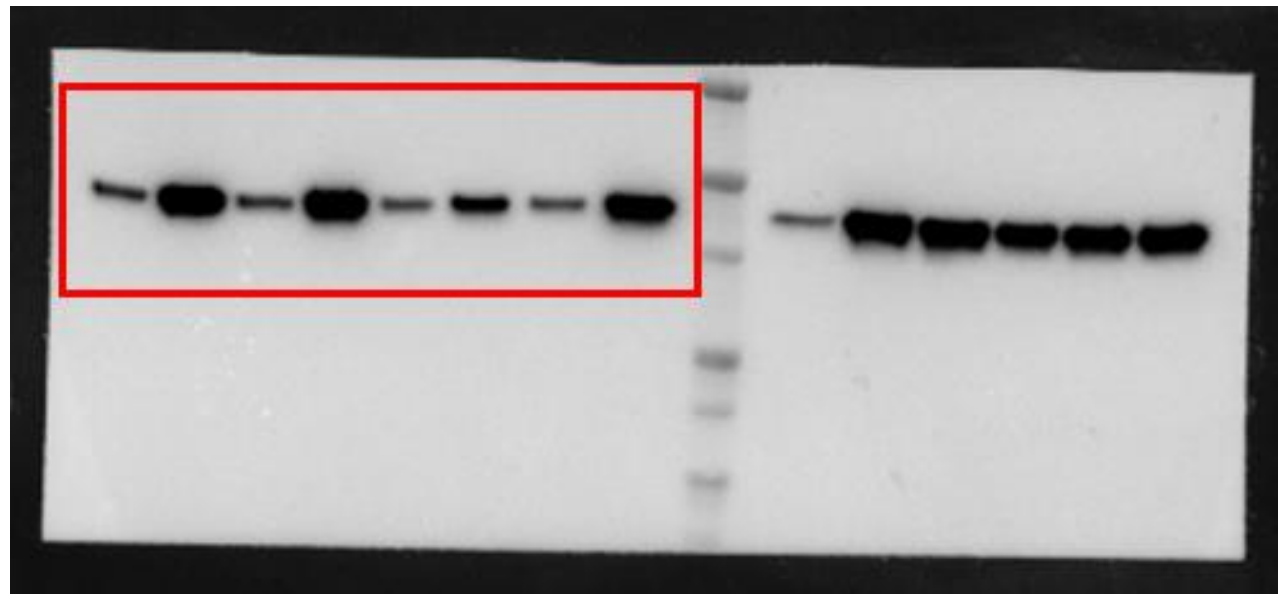

Total Protein

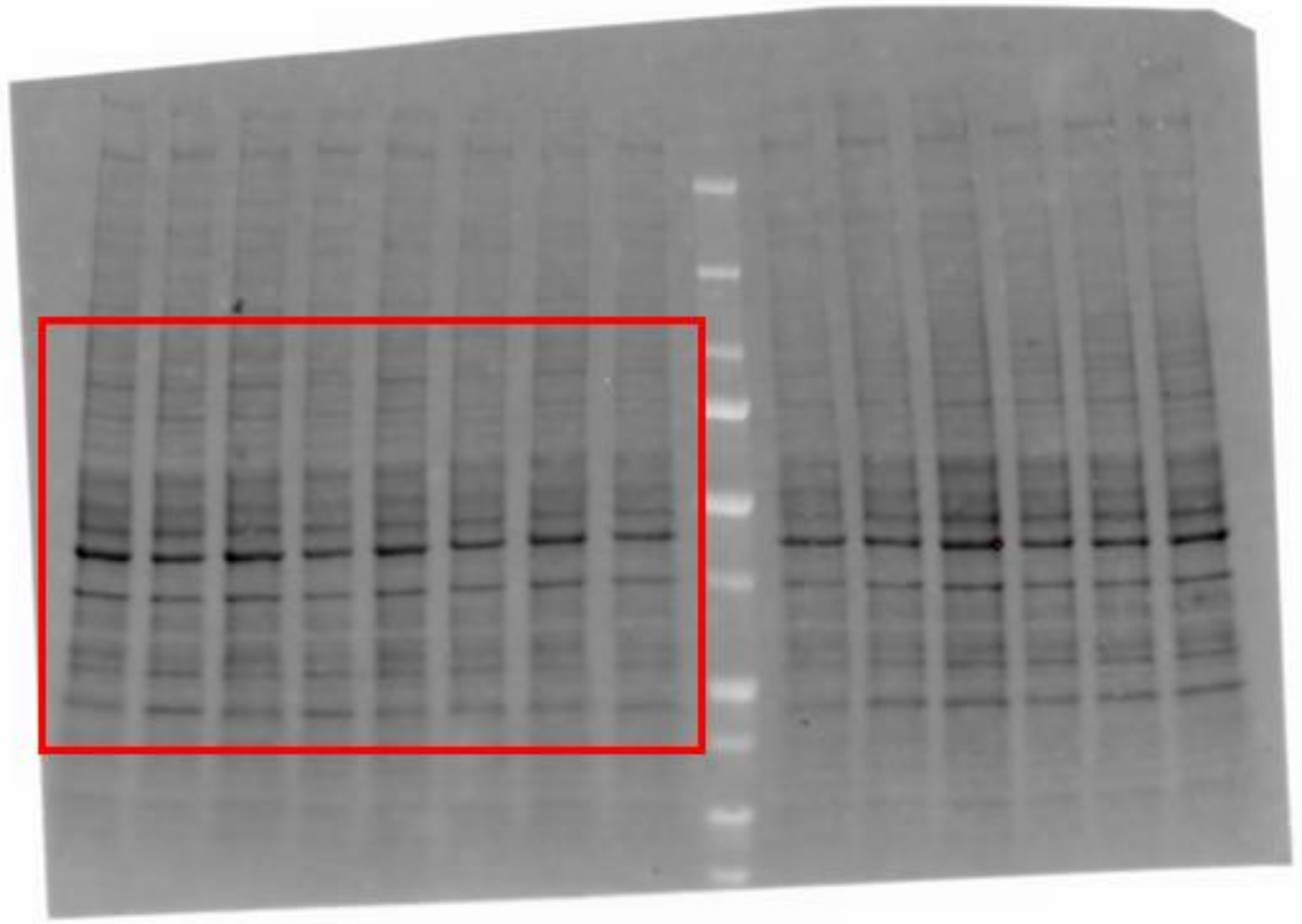

Full unedited gels for Fig. 6K

COL1A1 band

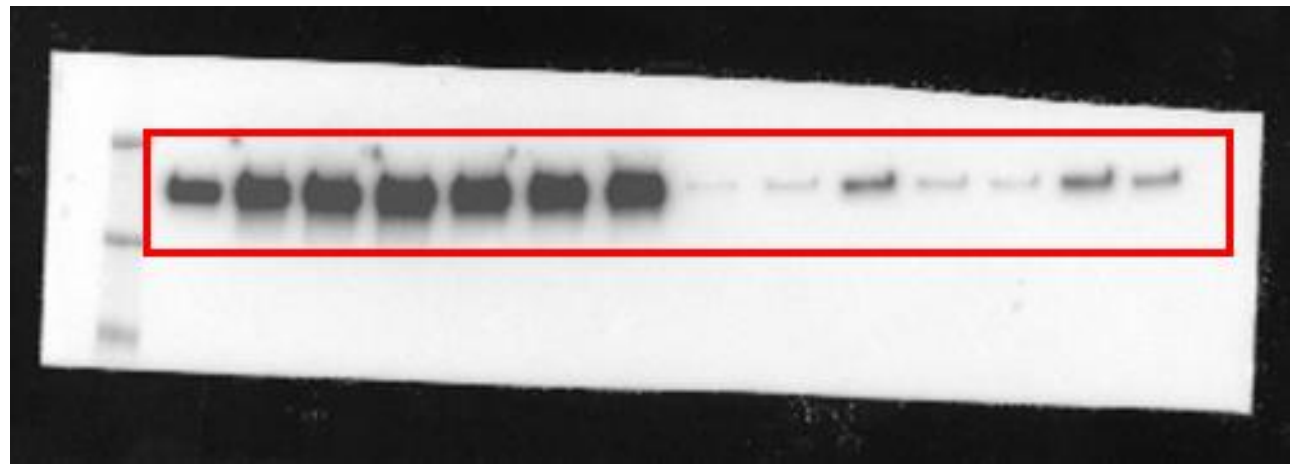

α-SMA band

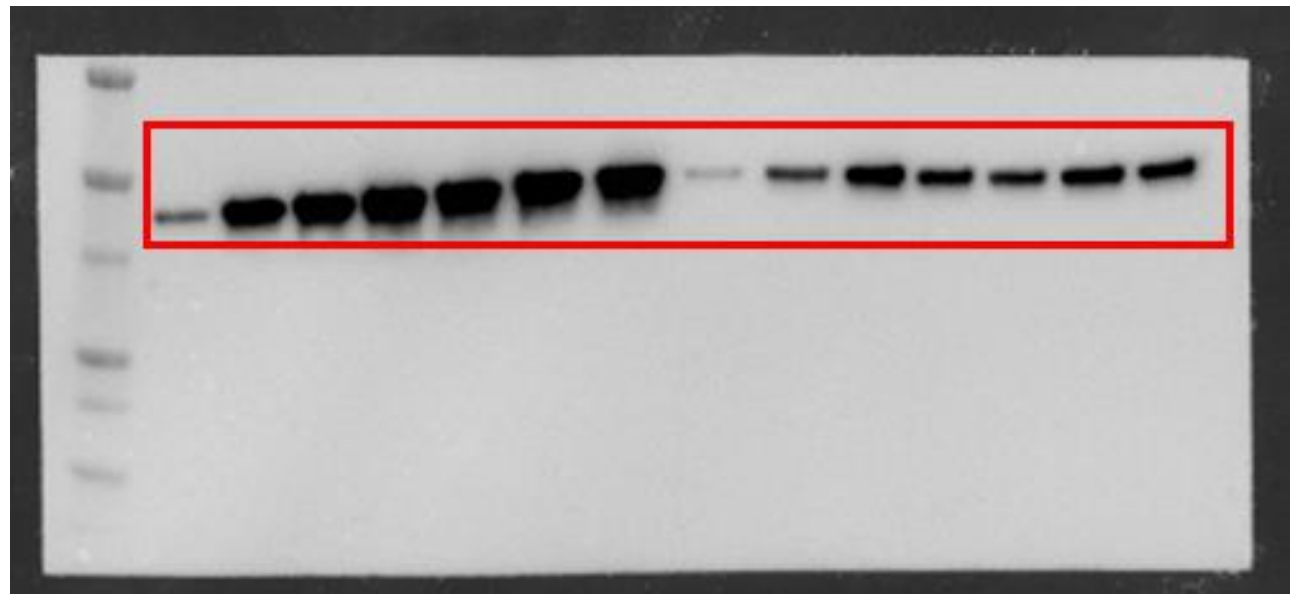

Total Protein

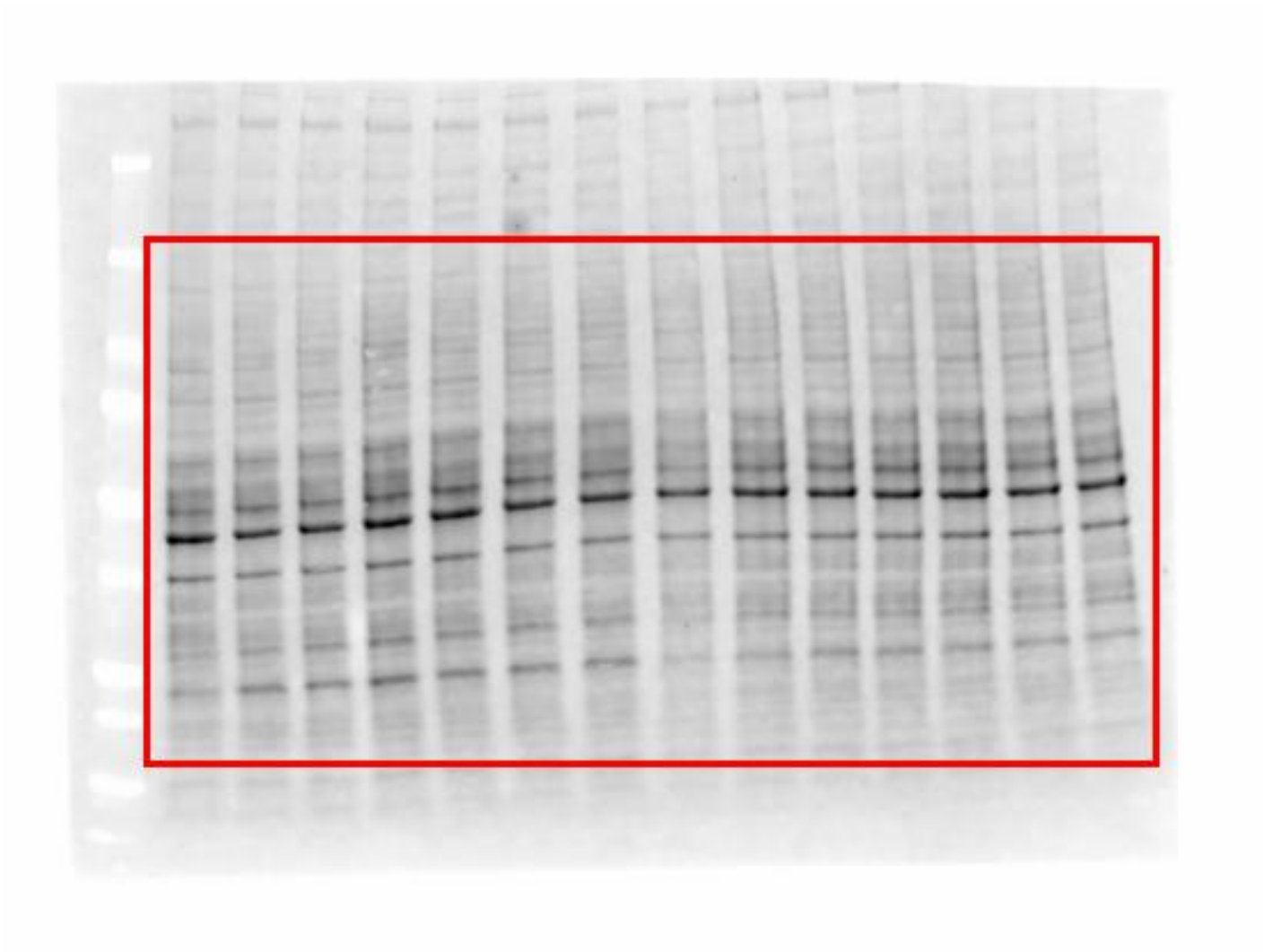

Full unedited gels for Fig. 7M

COL1A1 band

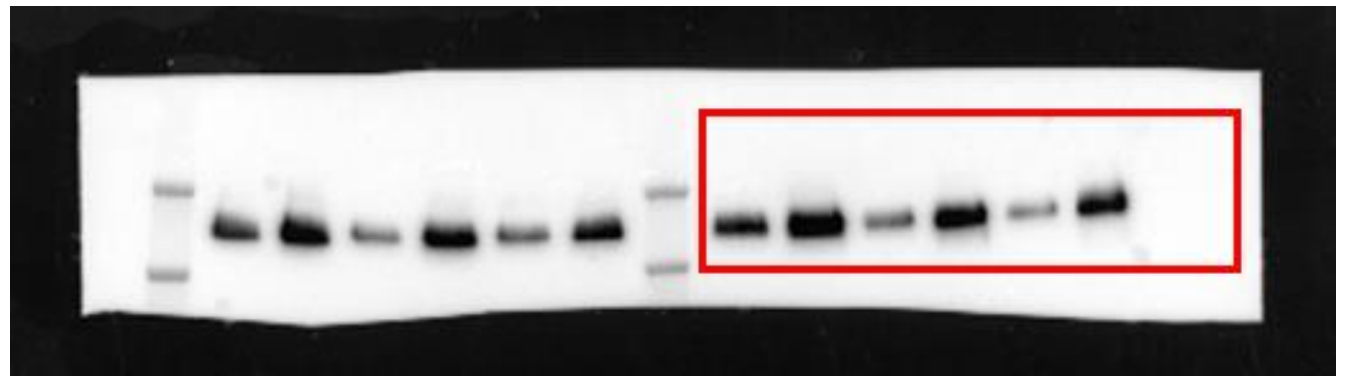

SLC38A2 band

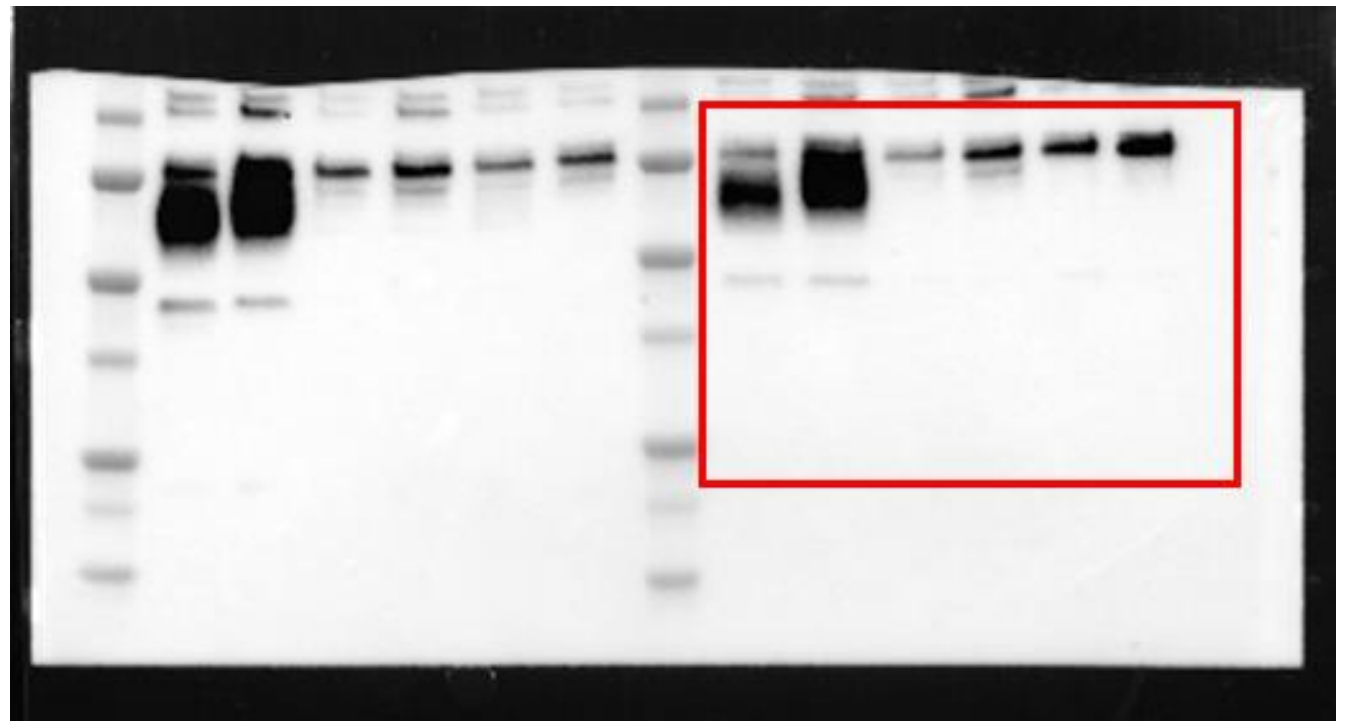

α-SMA band

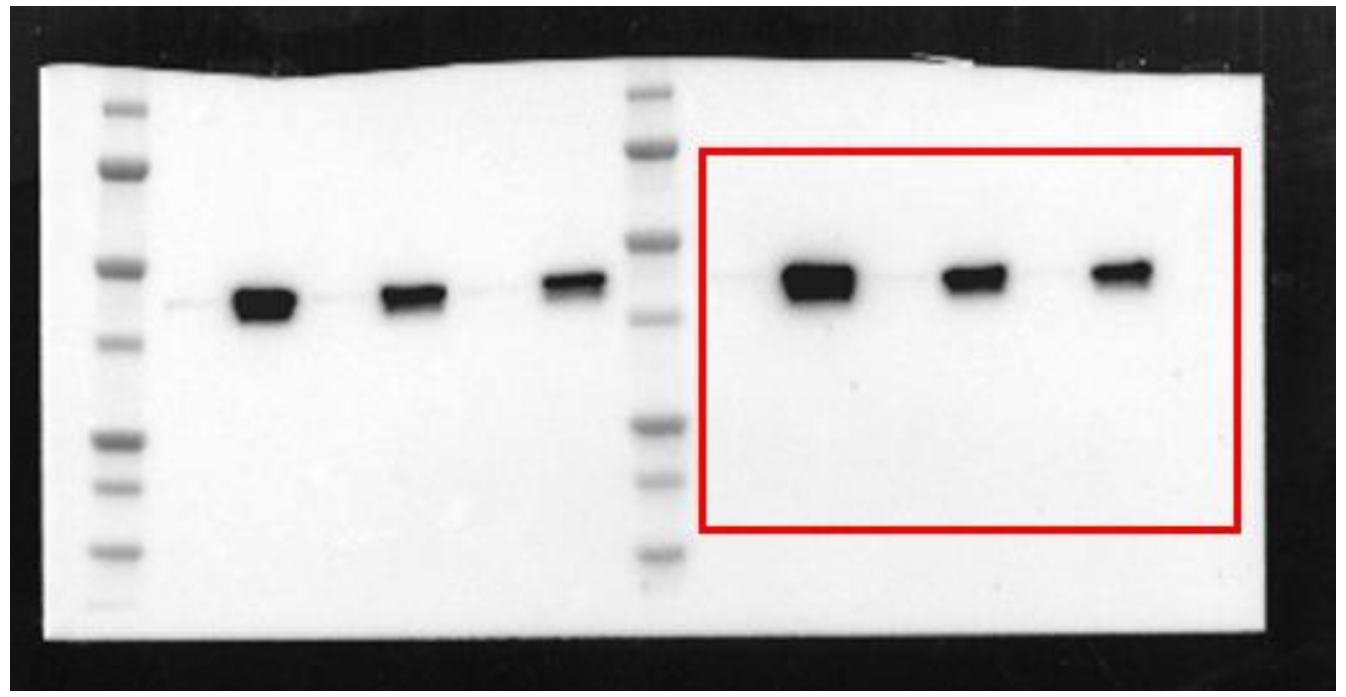

Total Protein

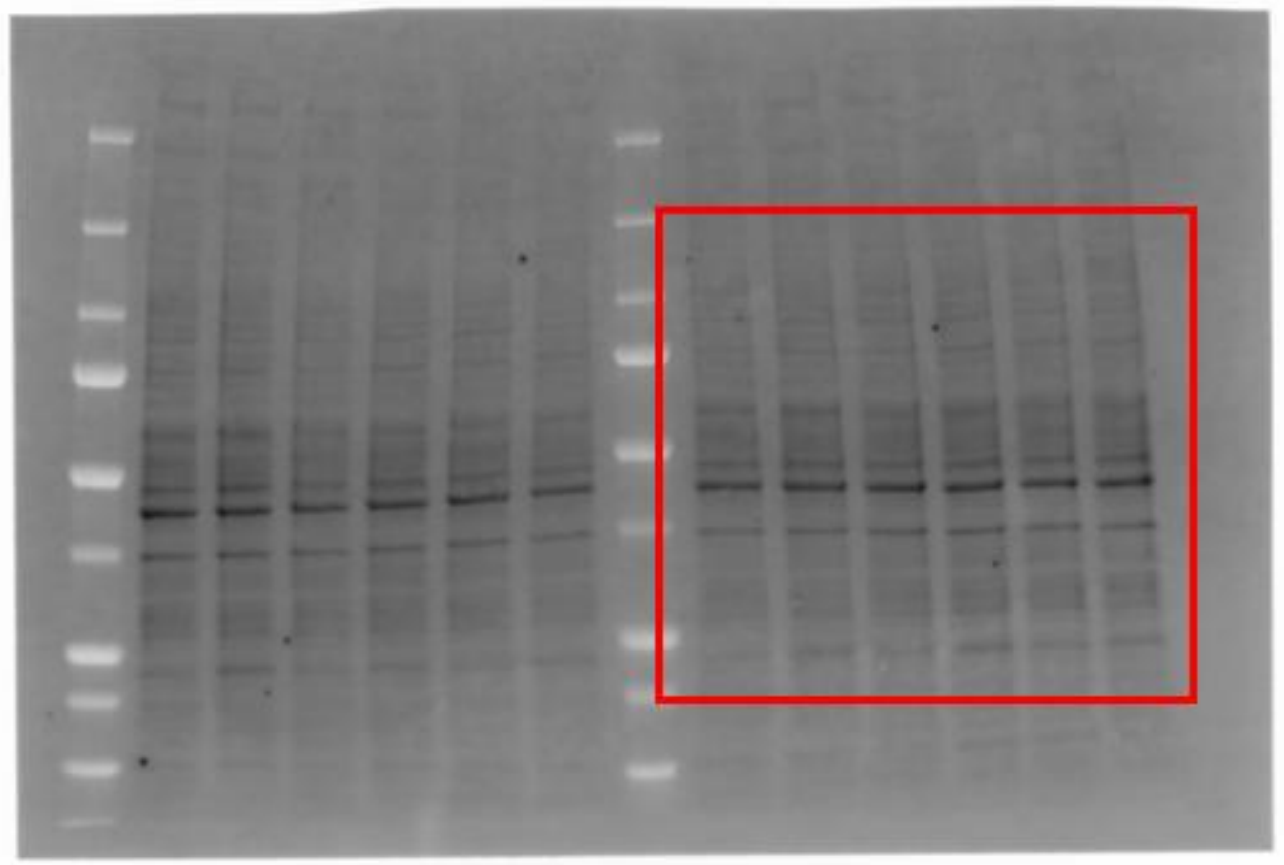

Full unedited gels for Fig. 8C

SLC38A2 band

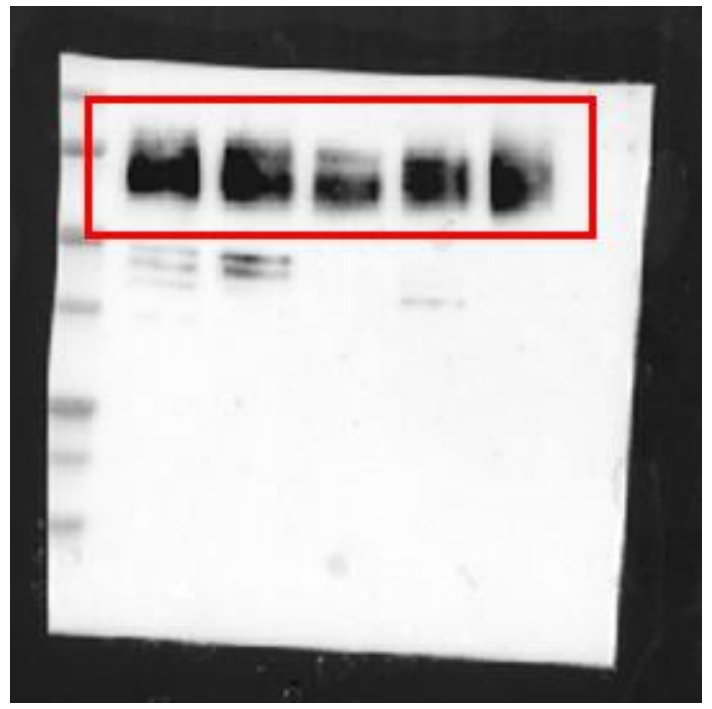

α-SMA band

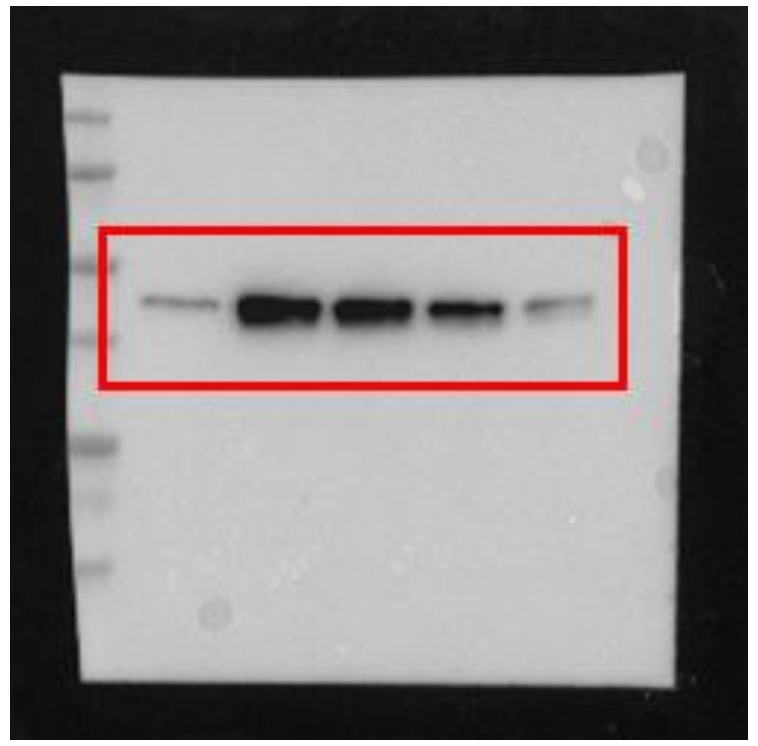

Total Protein

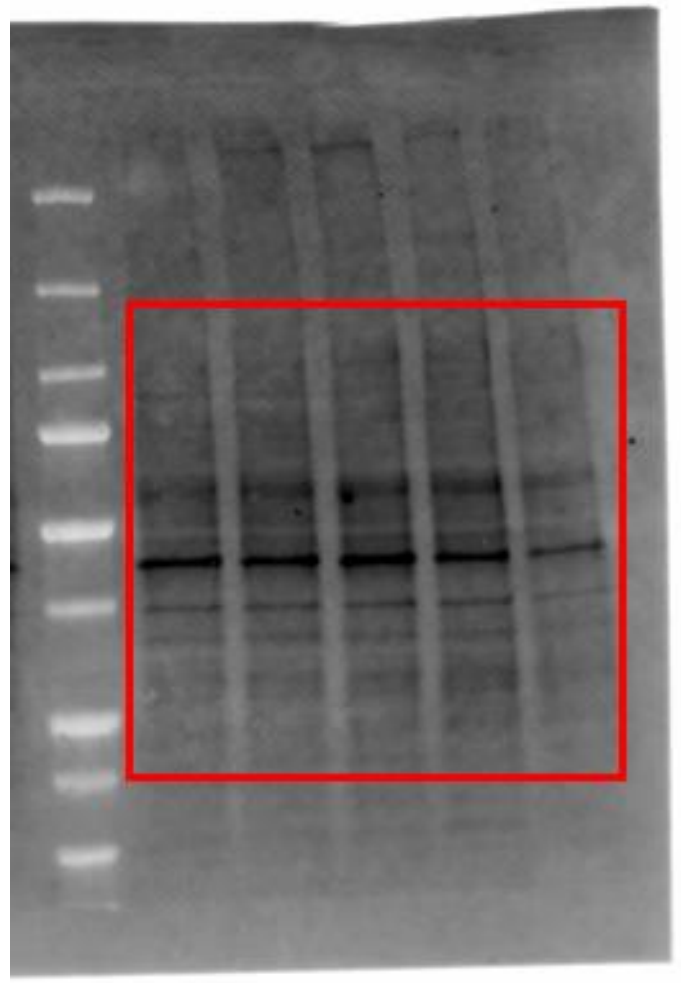

Full unedited gels for Fig. 8F

FN band

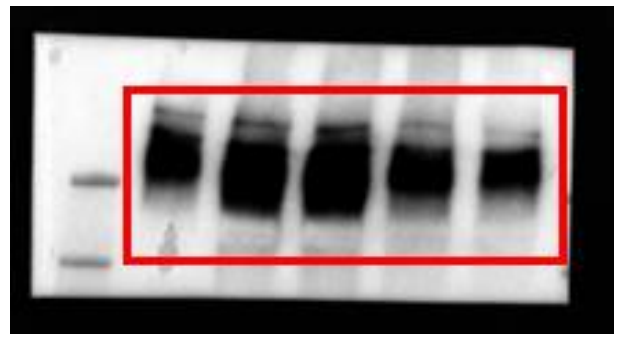

COL1A1 band

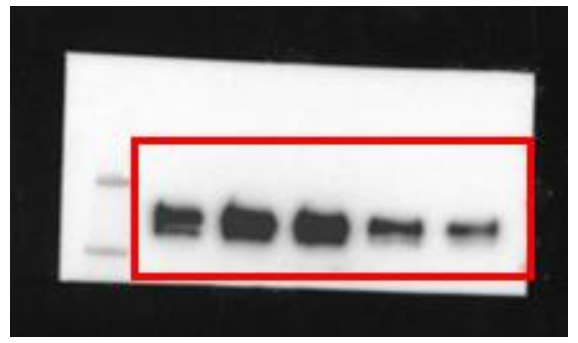

SLC38A2 band

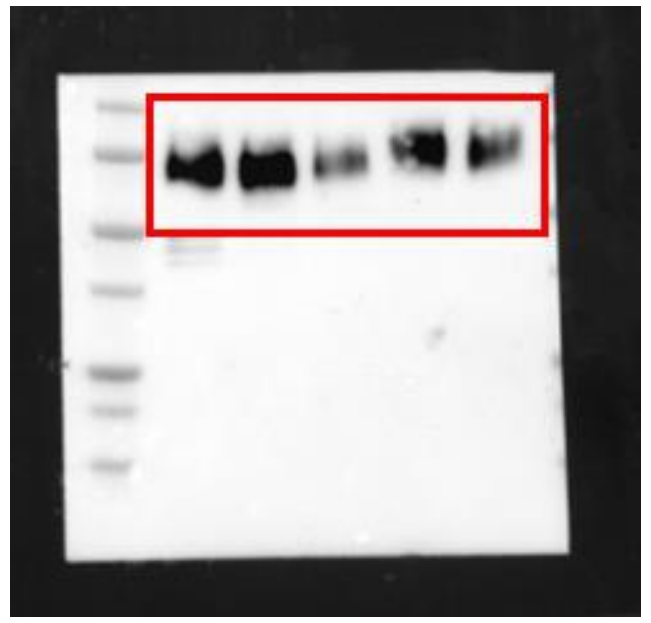

Total Protein

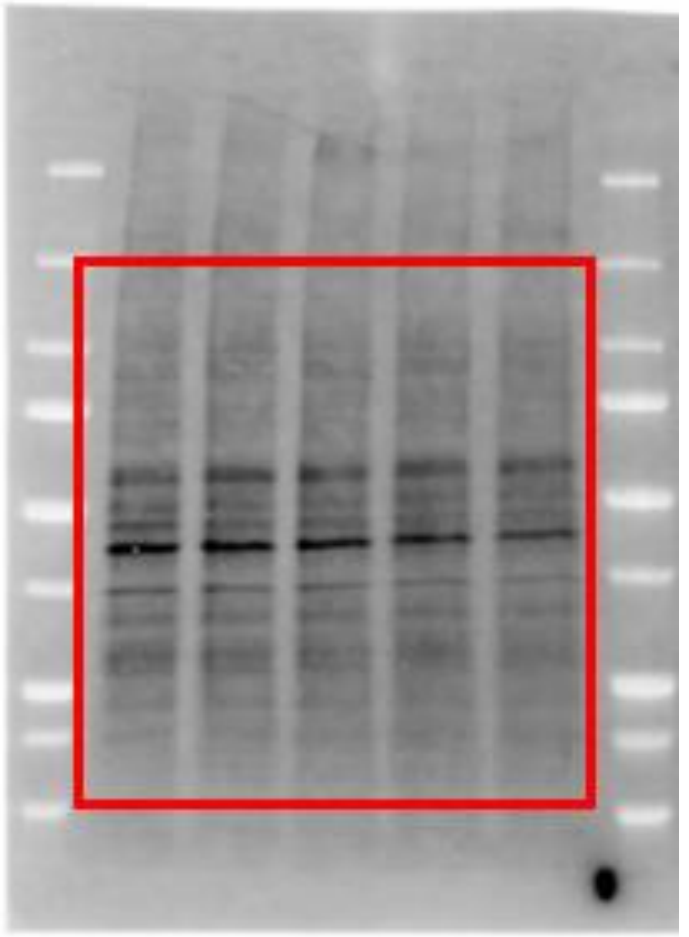

Full unedited gels for Fig. S2A

GPT2 band

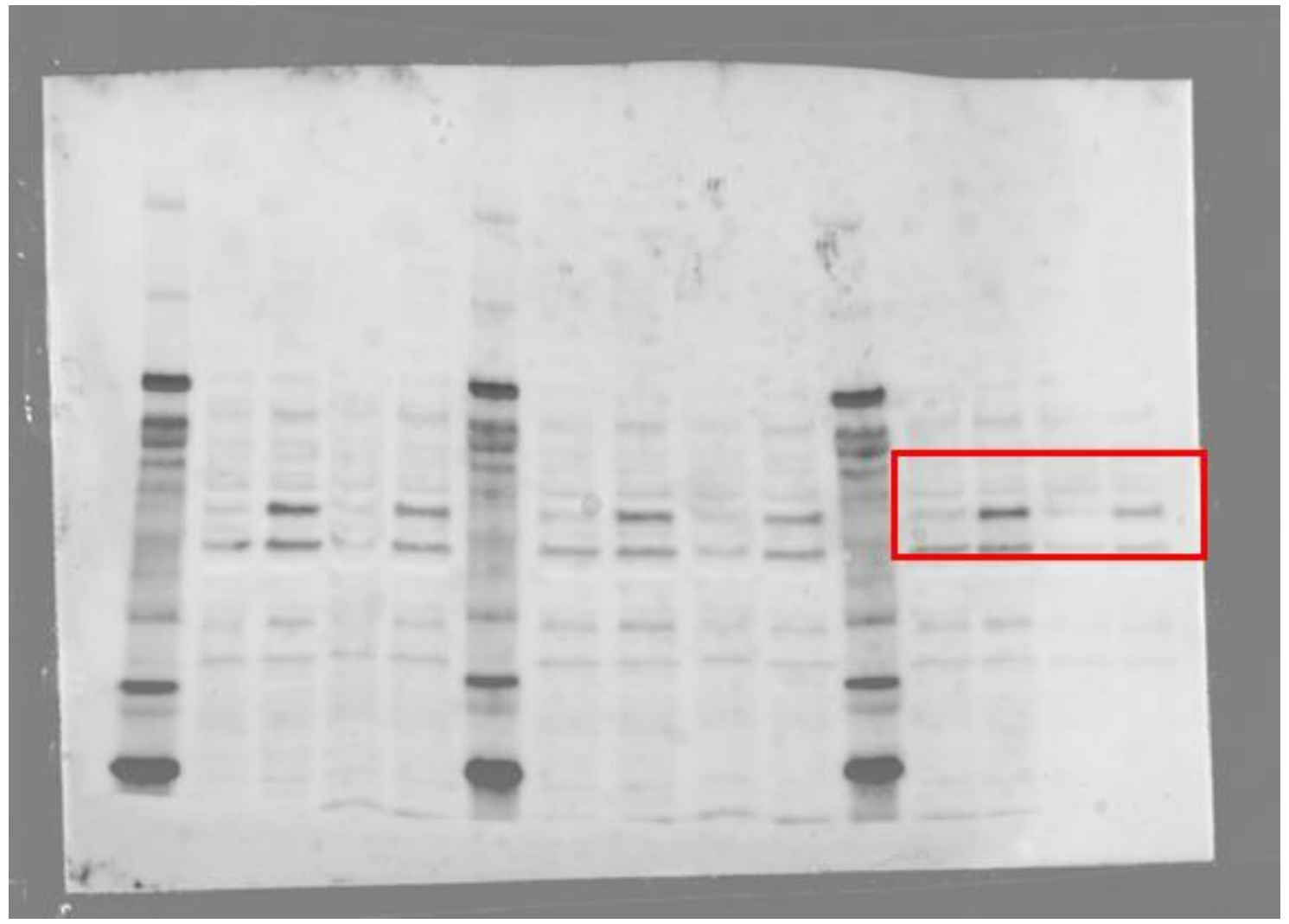

SLC1A5 band

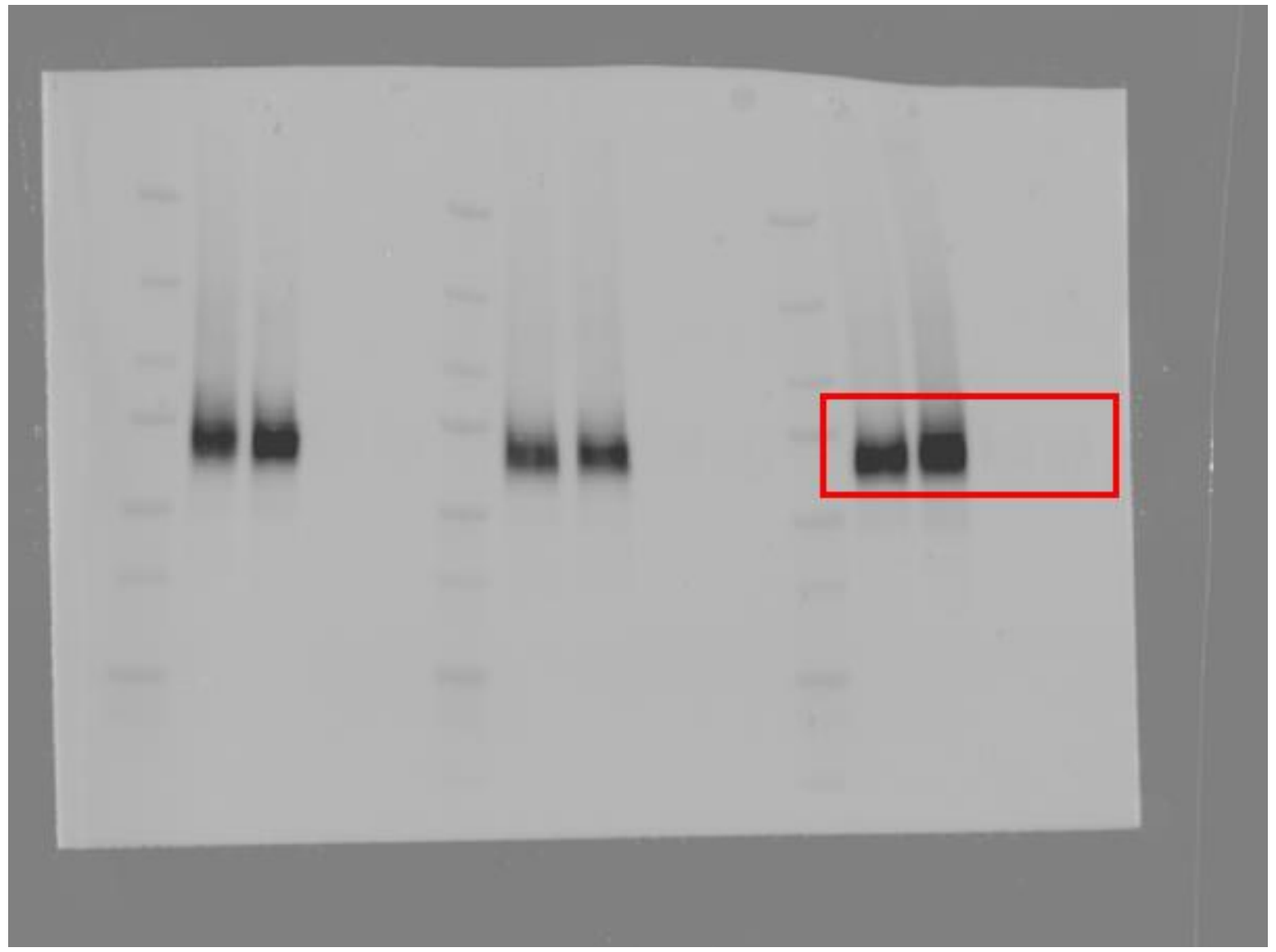

Total Protein

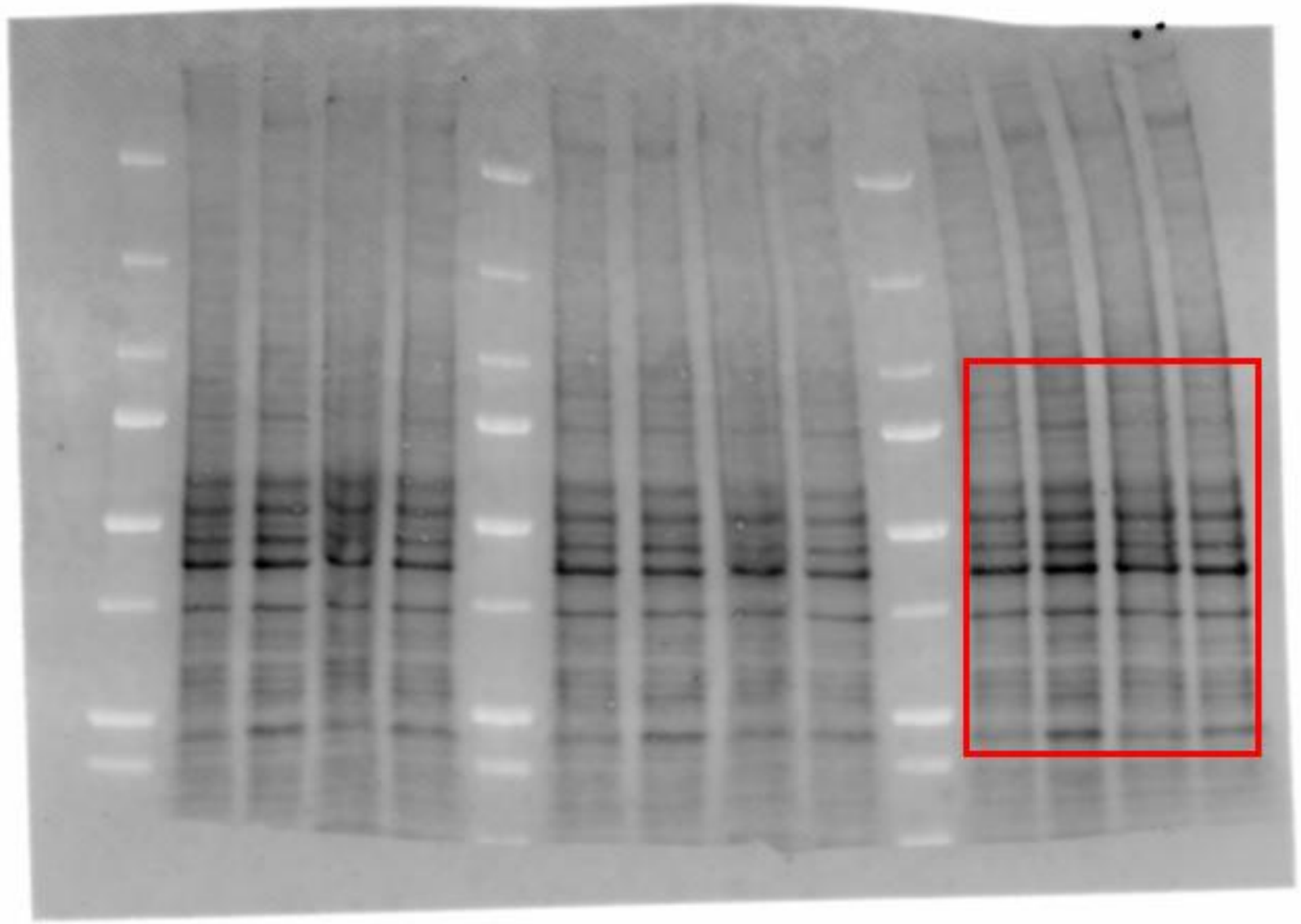

Full unedited gels for Fig. S2B

GPT2 band

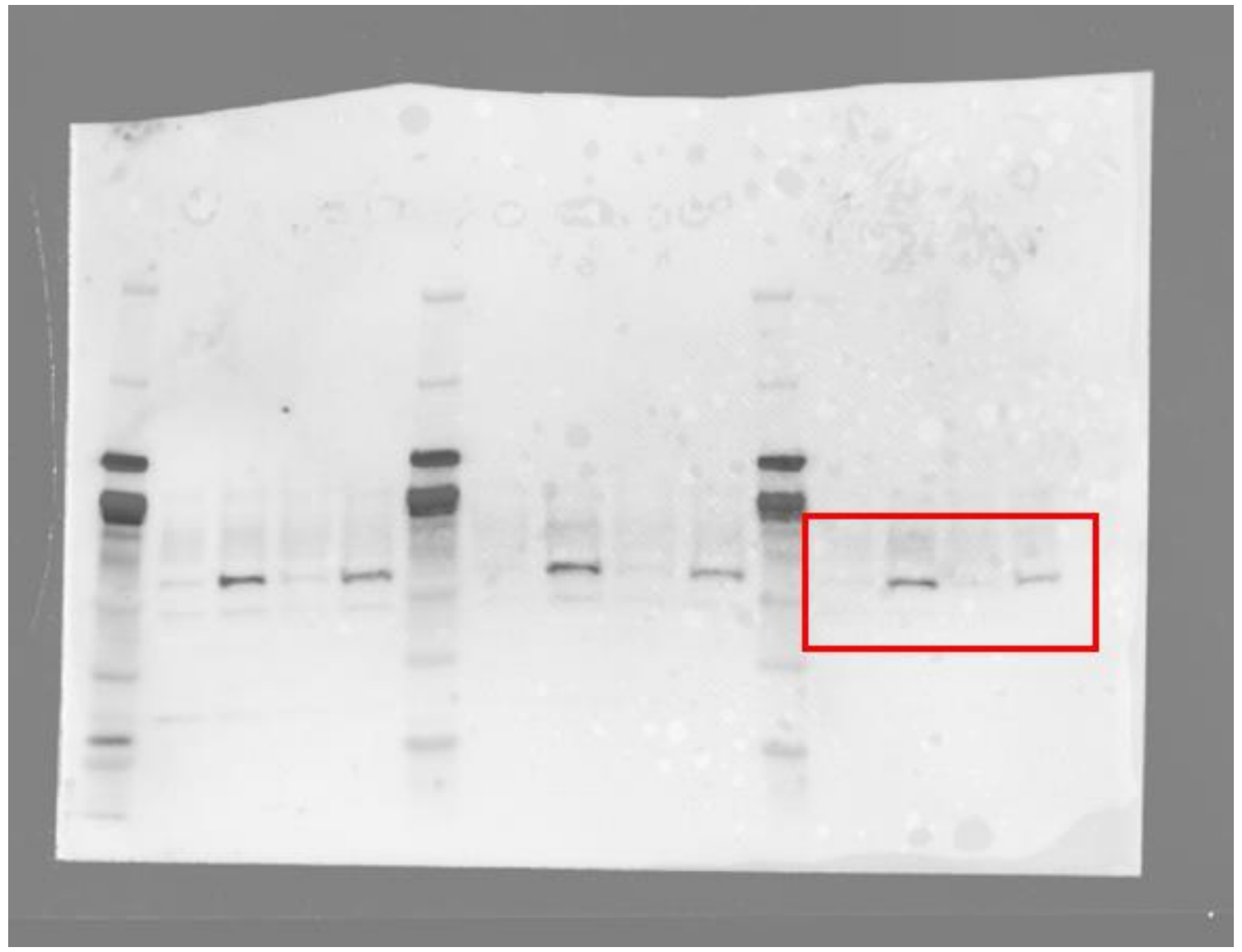

Total Protein

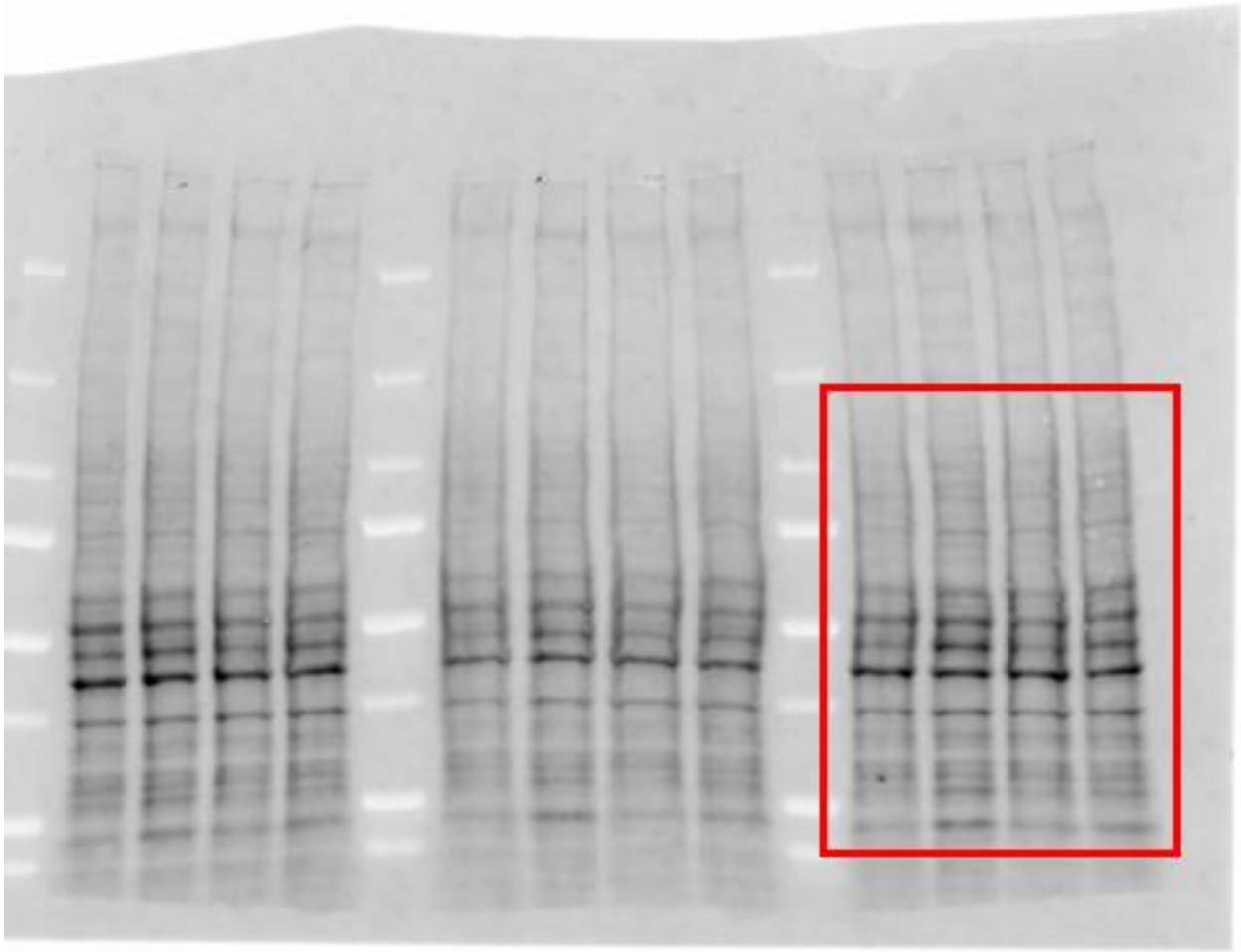

Full unedited gels for Fig. S2G

COL1A1 band

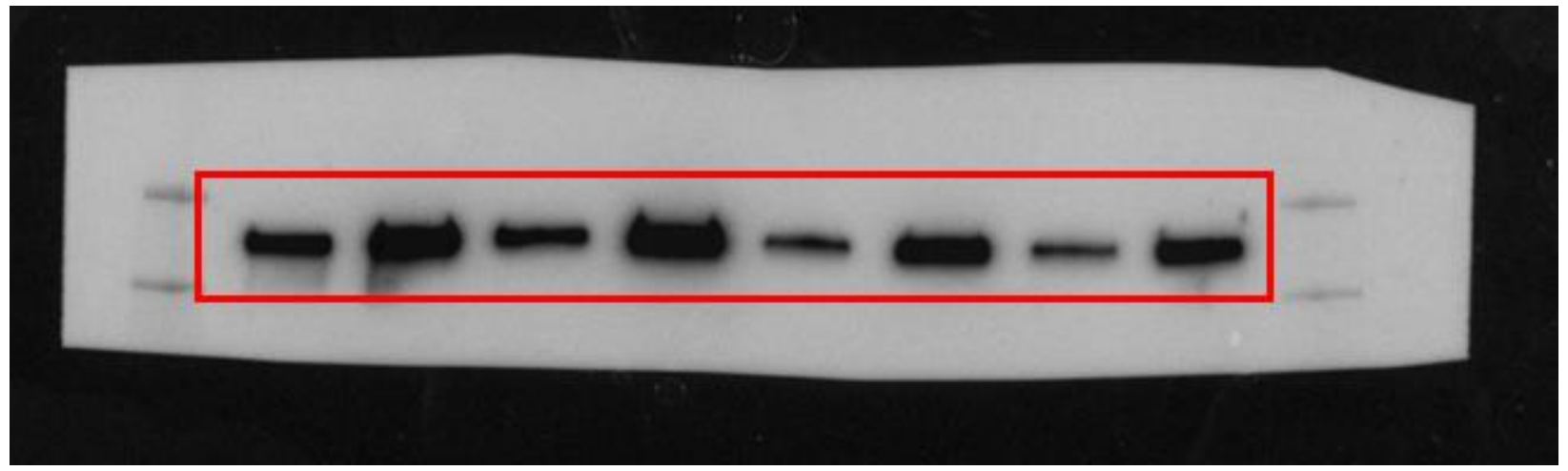

GPT1 band

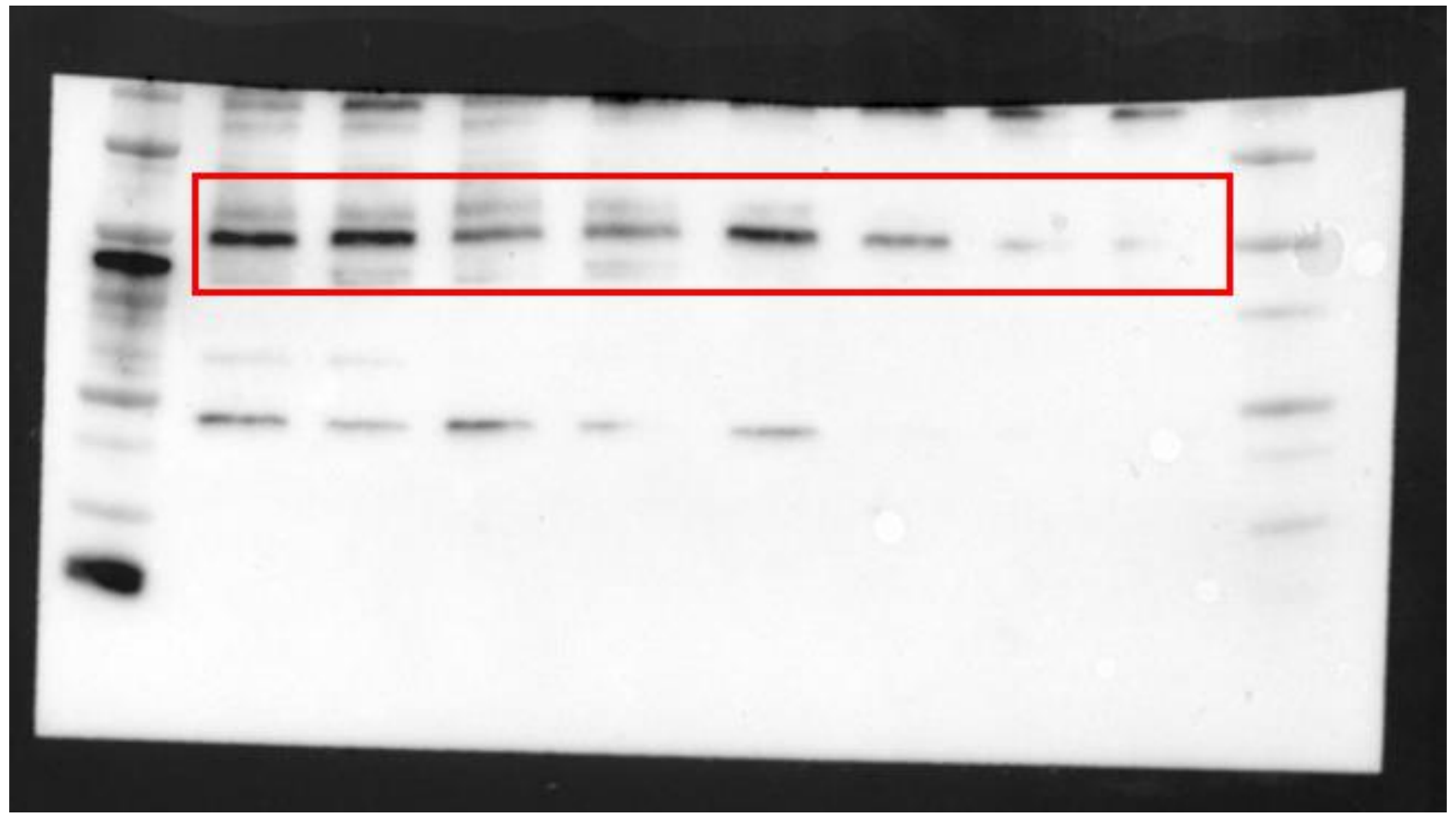

α-SMA band

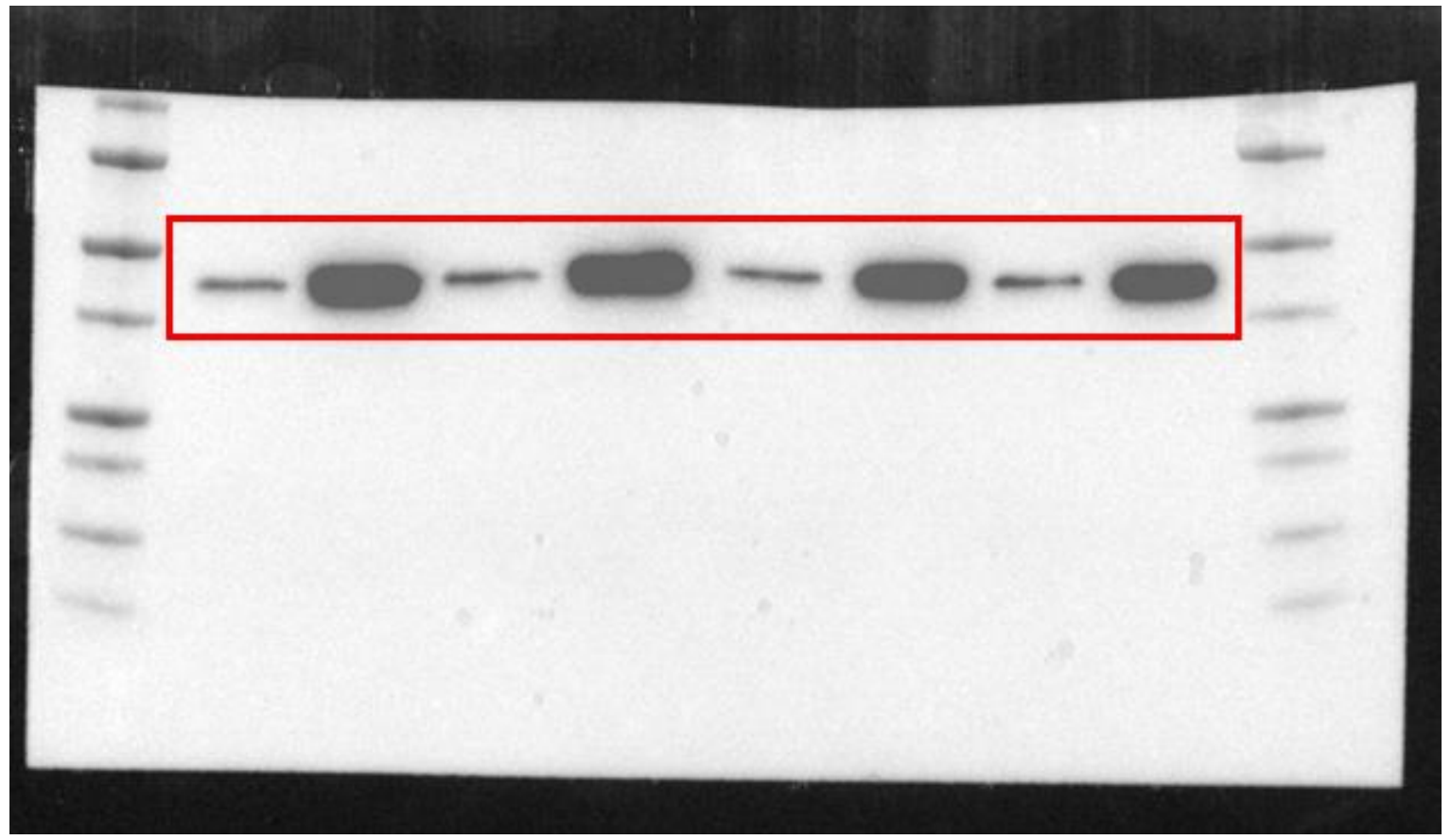

Total Protein

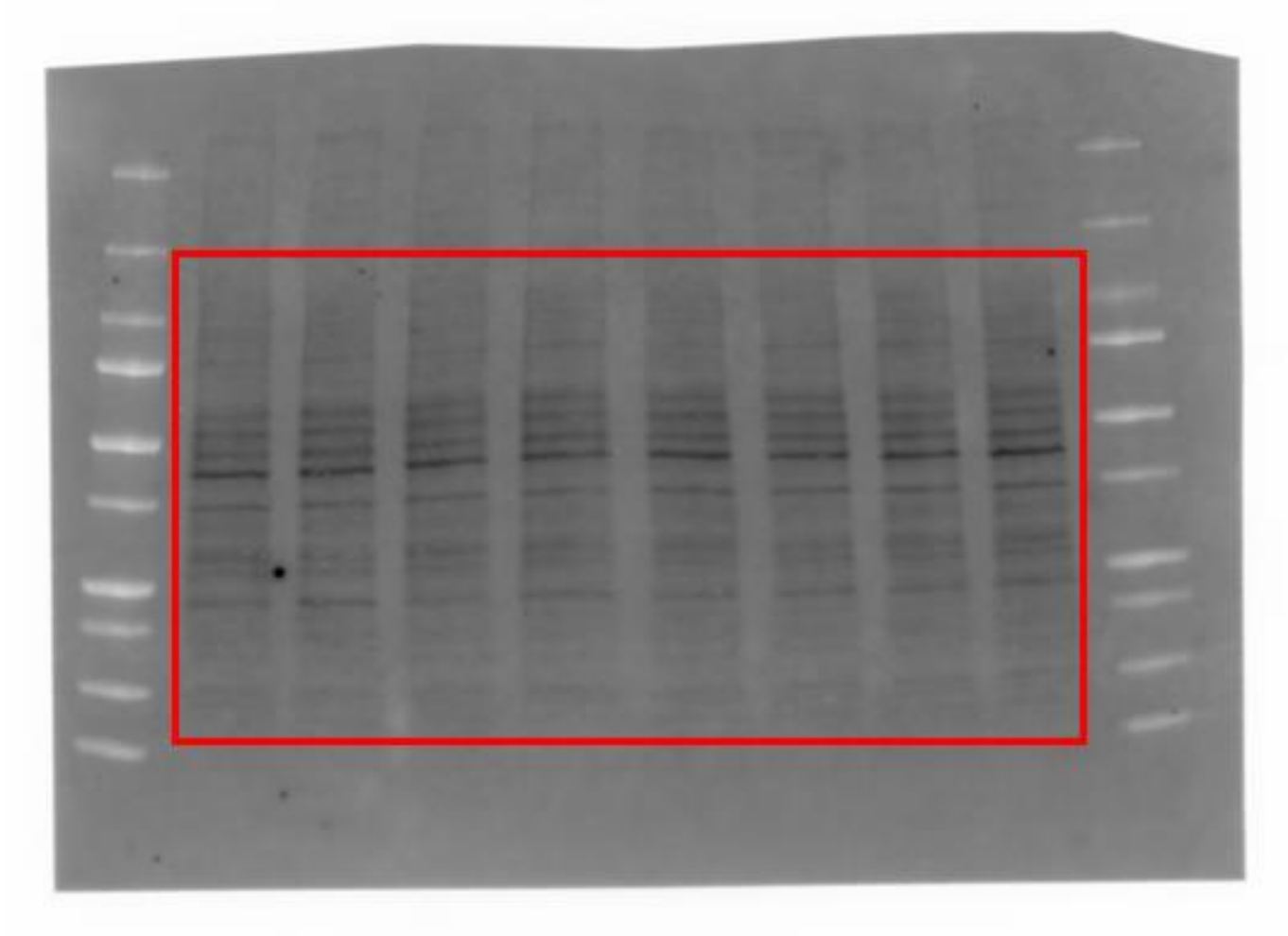

Full unedited gels for Fig. S2I

P-smad3 band

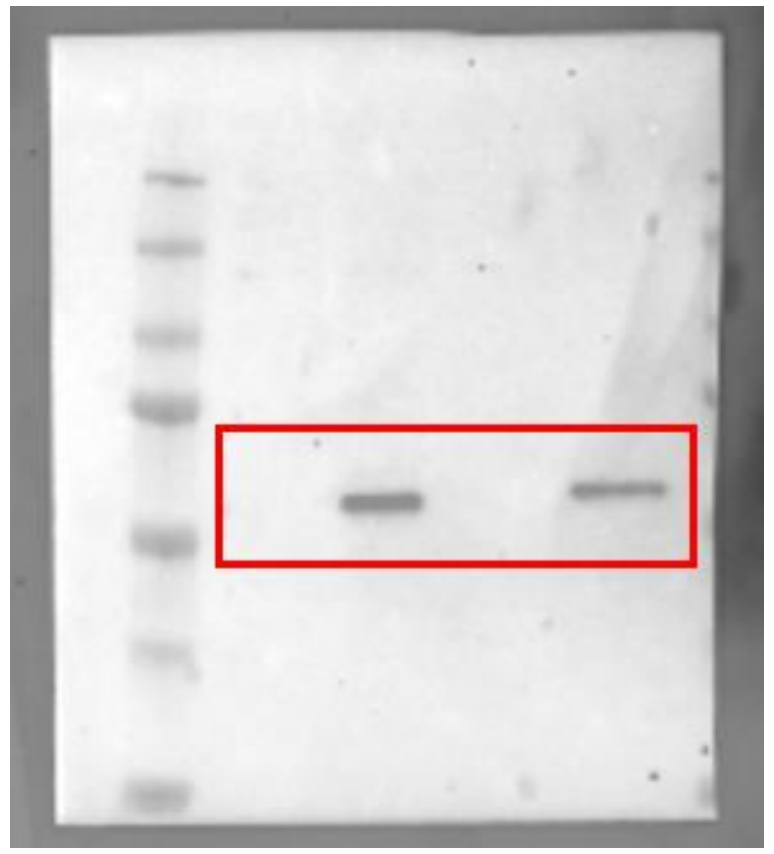

Smad 3 band

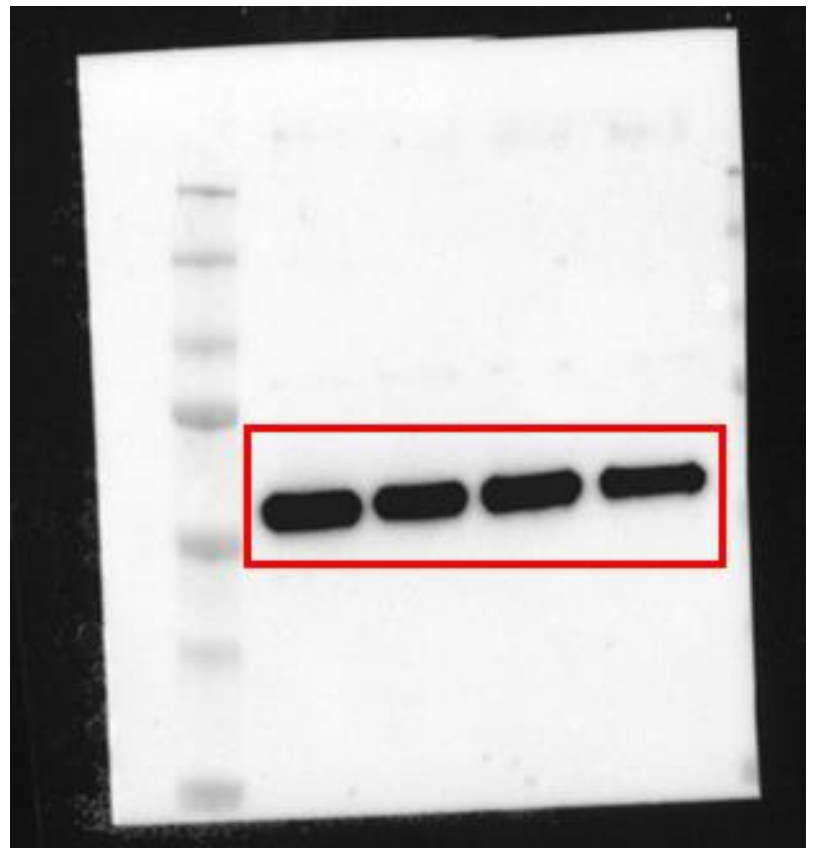

Total Protein

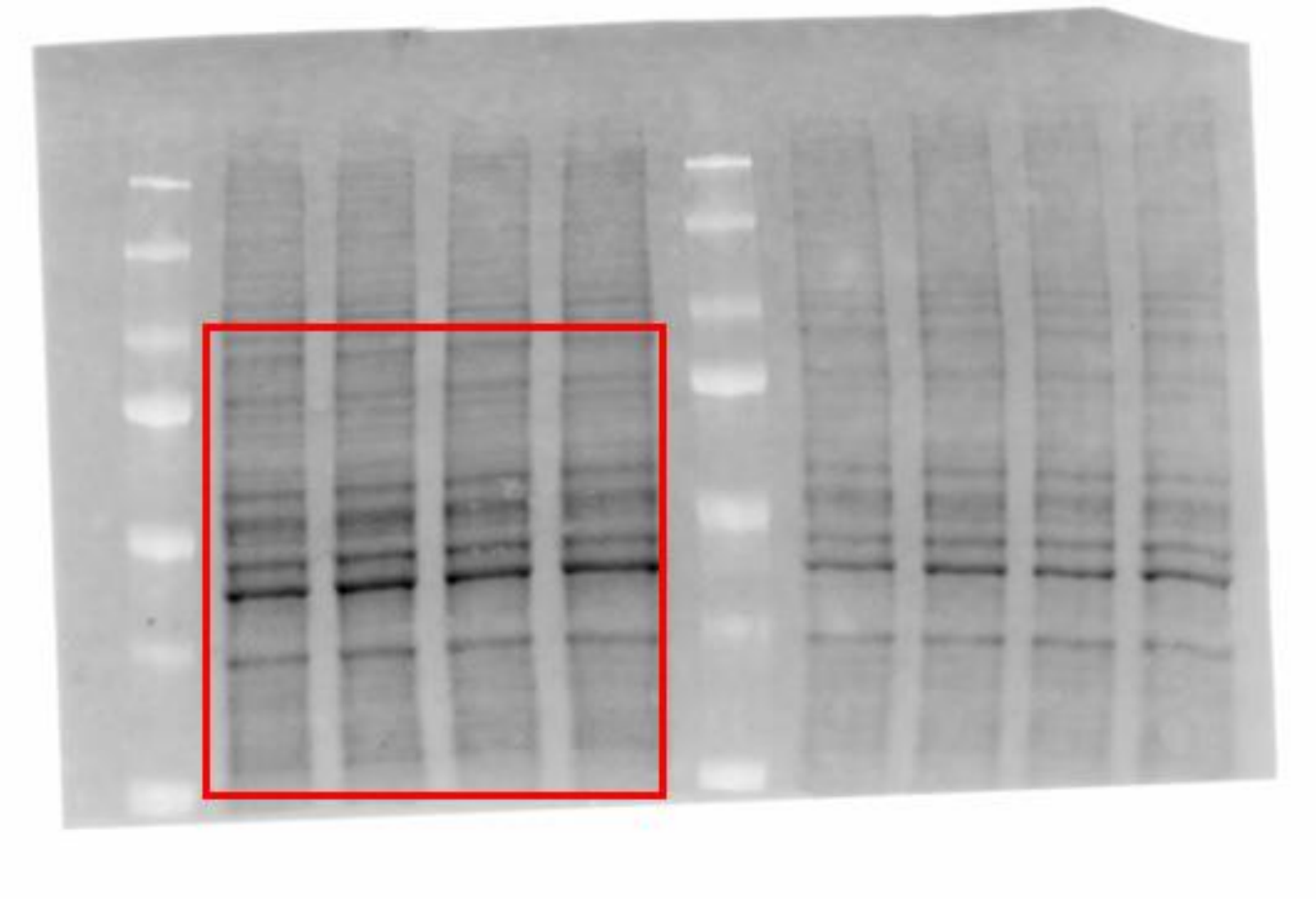

Full unedited gels for Fig. S4A

SLC38A2 band GPT2 band α-SMA band COL1A1 band

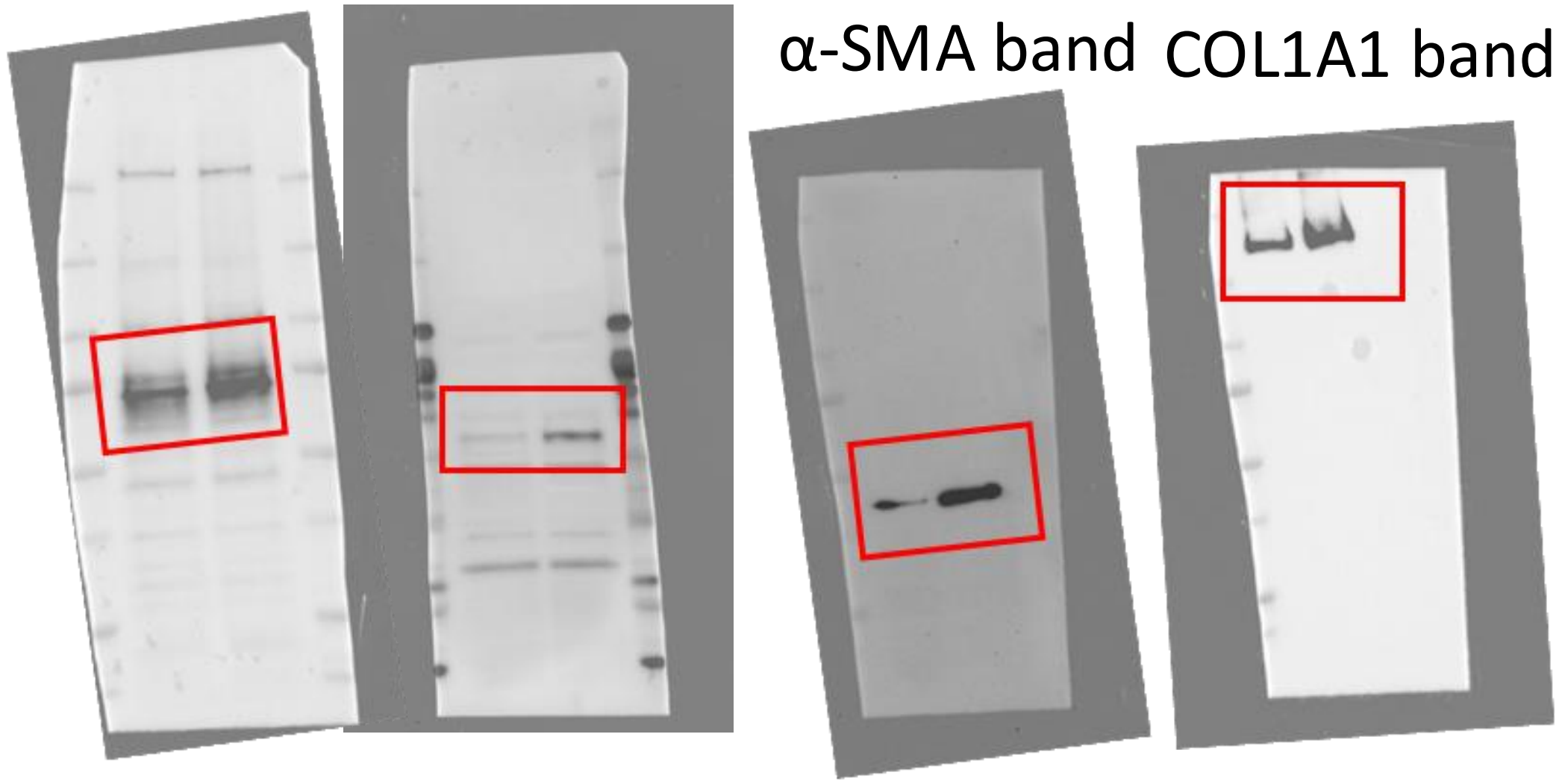

Total Protein

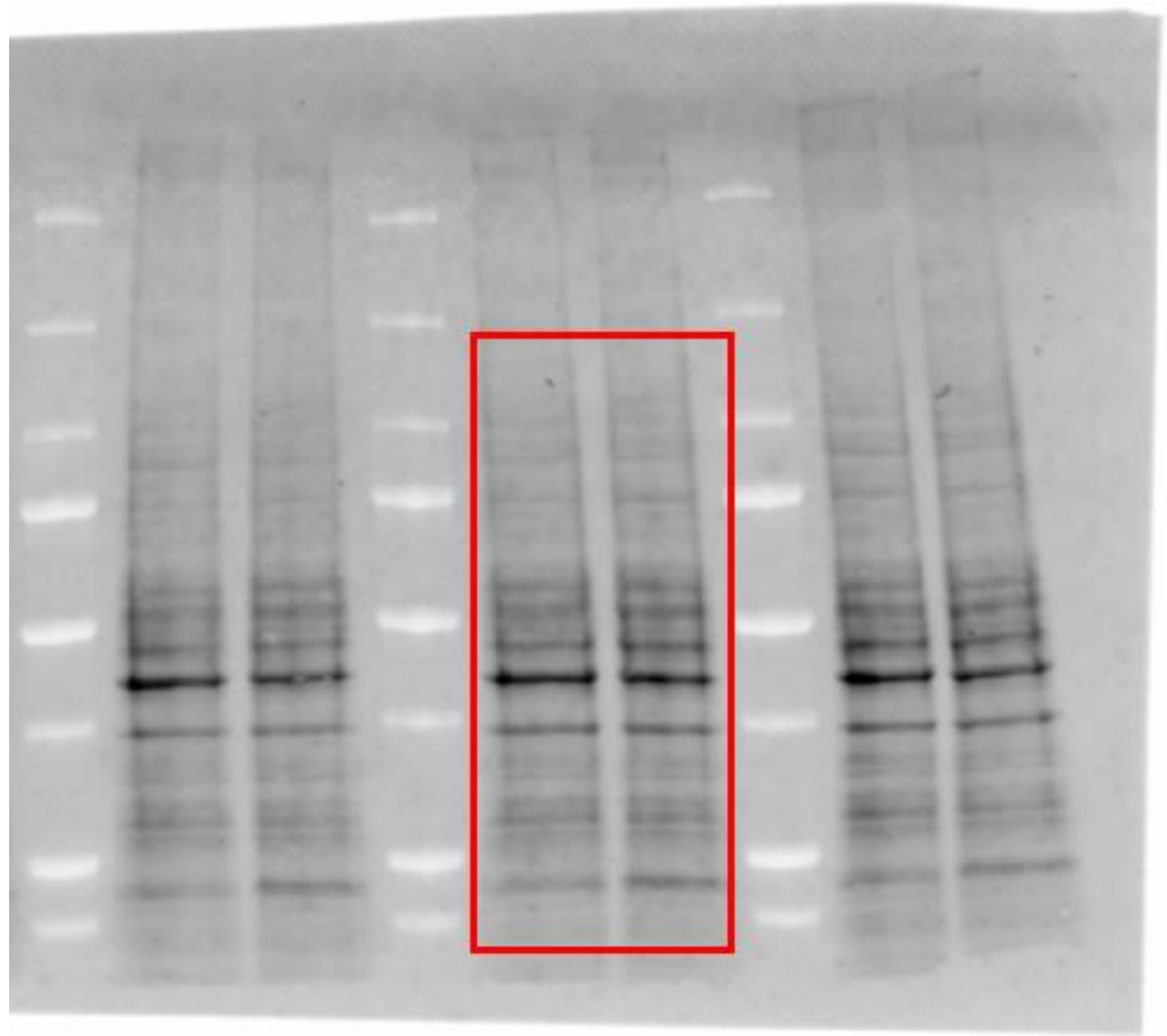

Full unedited gels for Fig. S5B

GLUT1 band

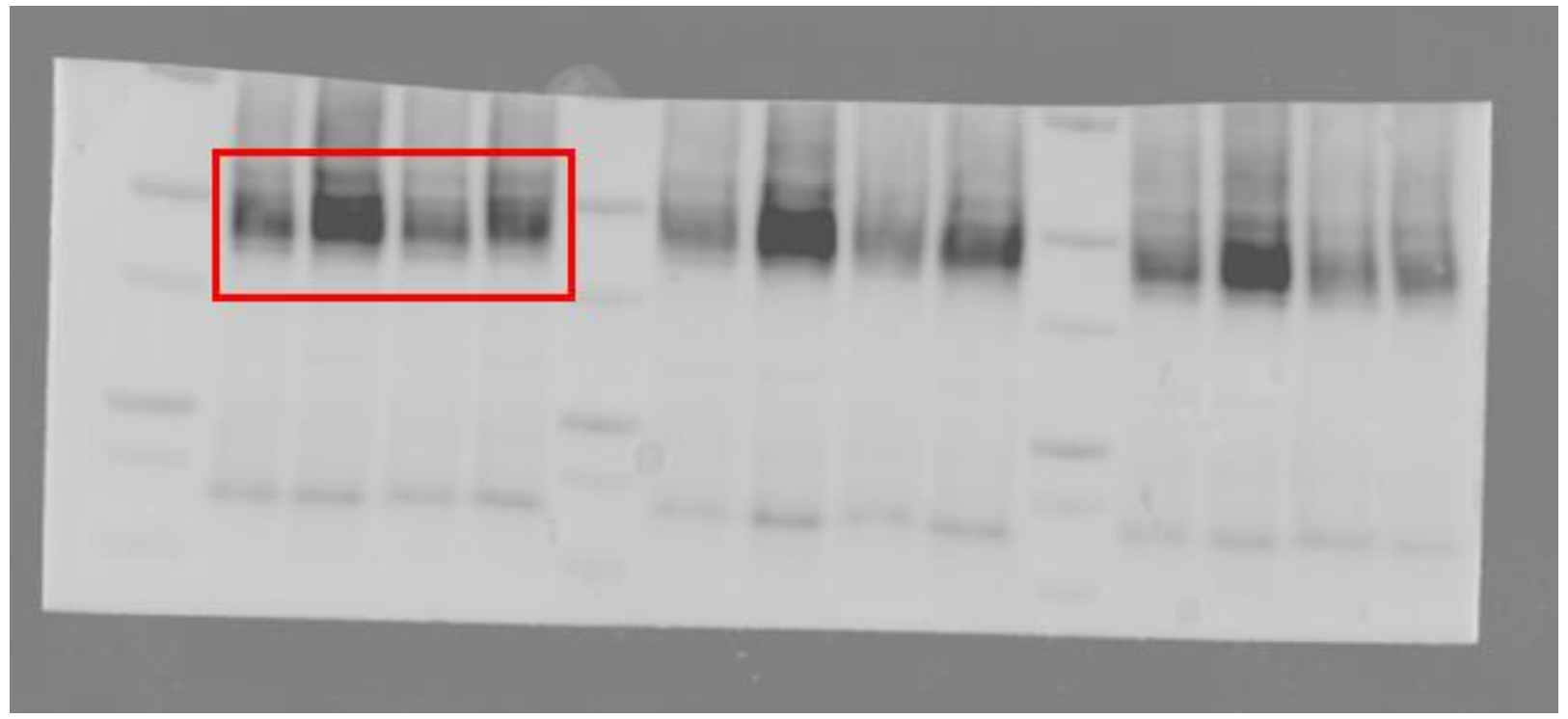

GPT2 band

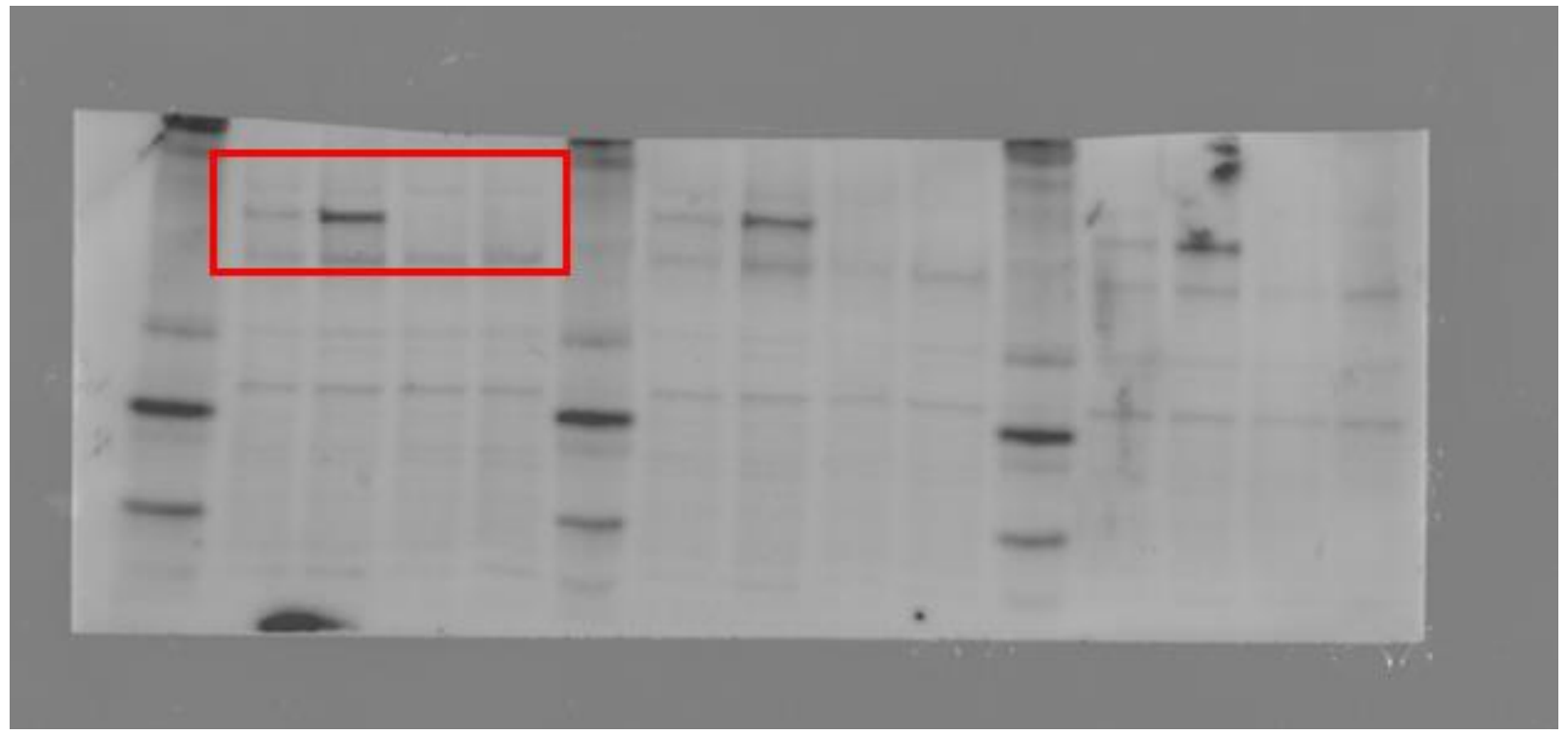

Total Protein

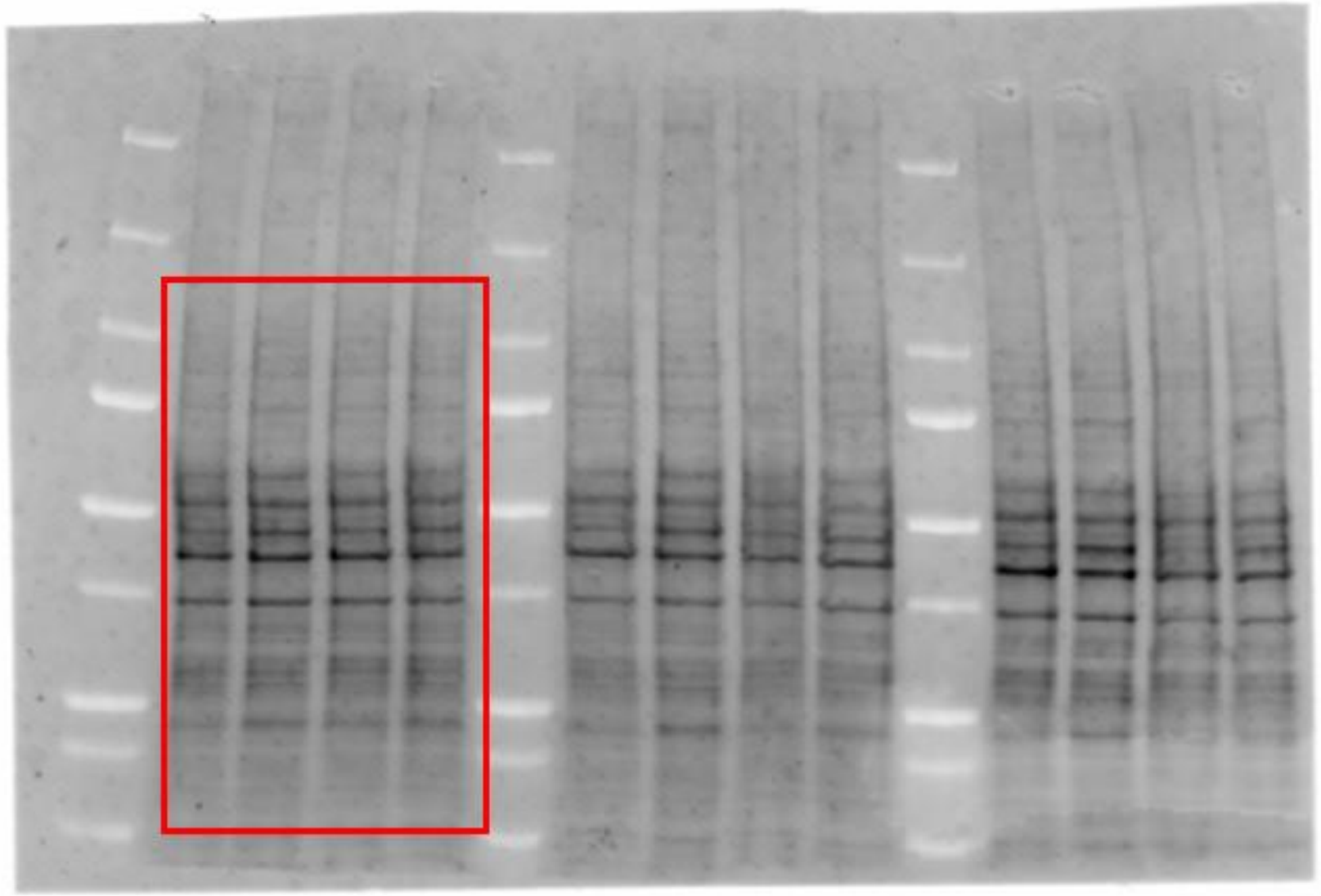

Full unedited gels for Fig. S7F

COL1A1 band

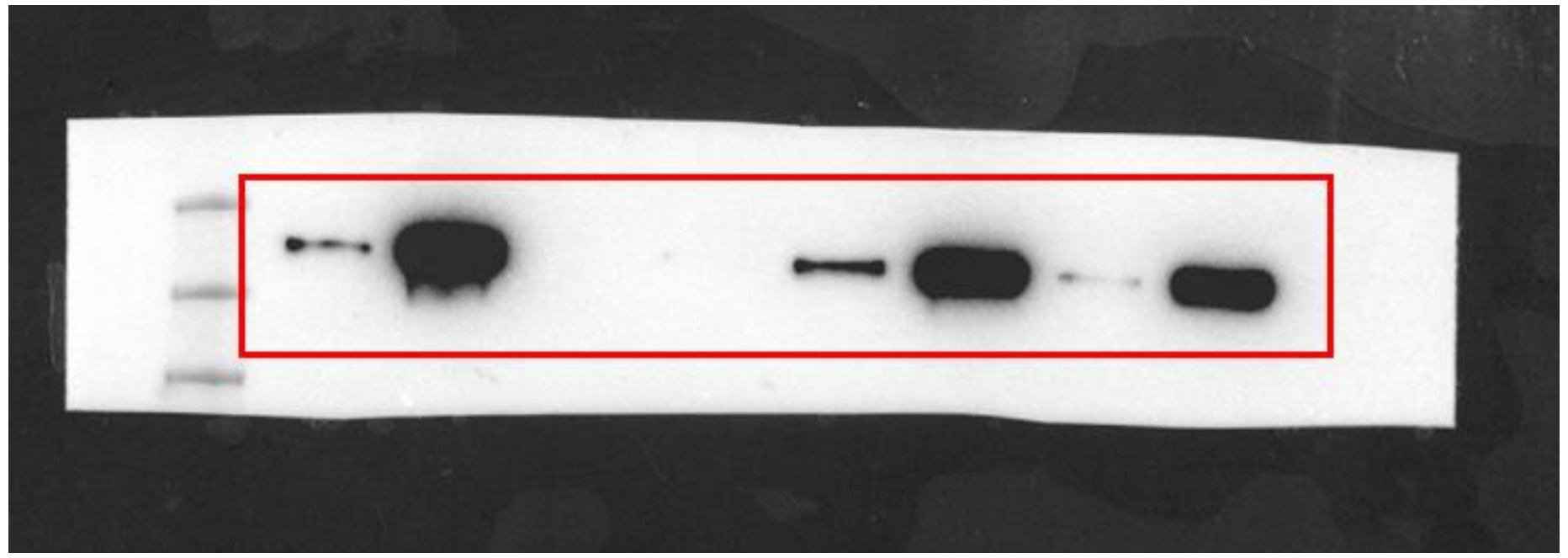

α-SMA band

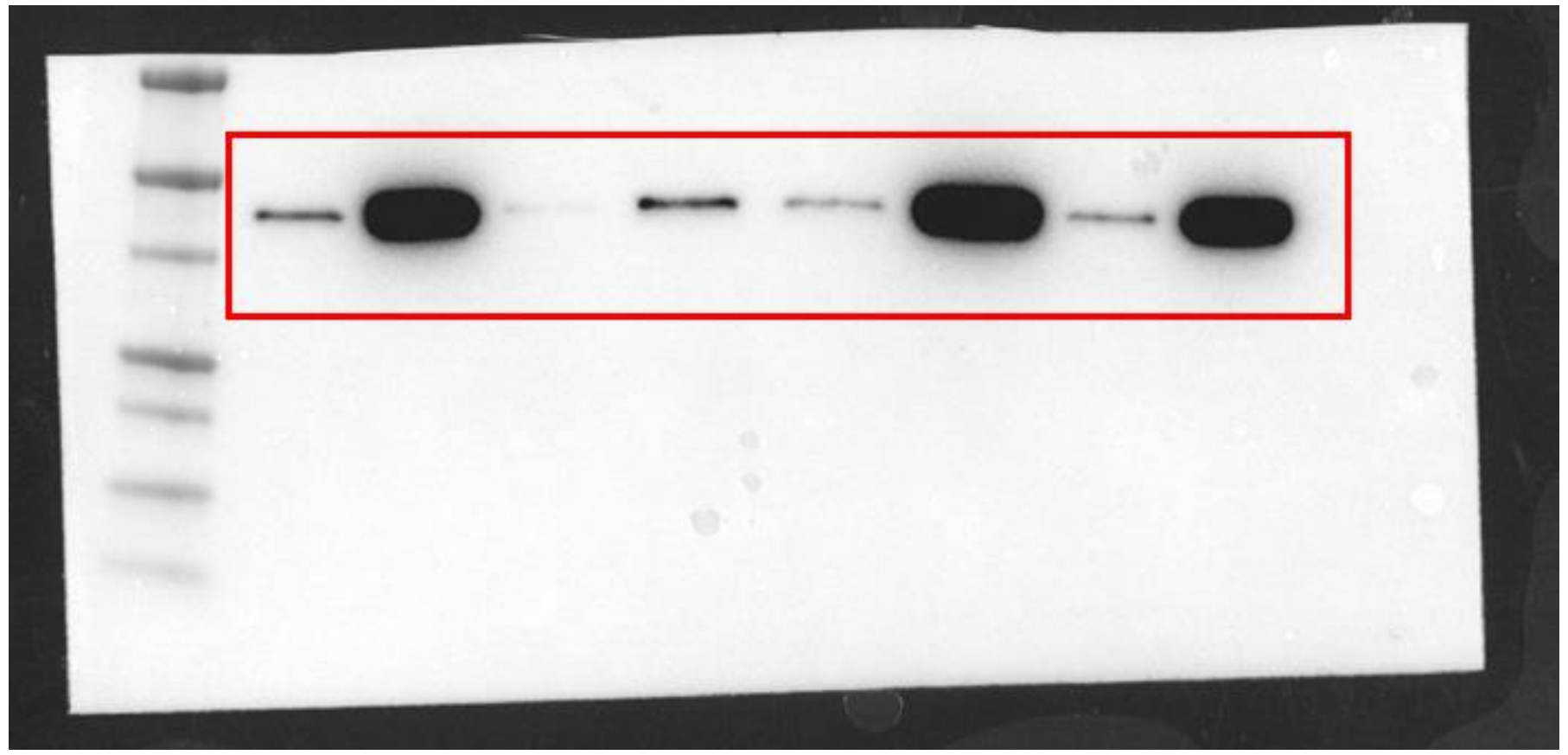

Total Protein

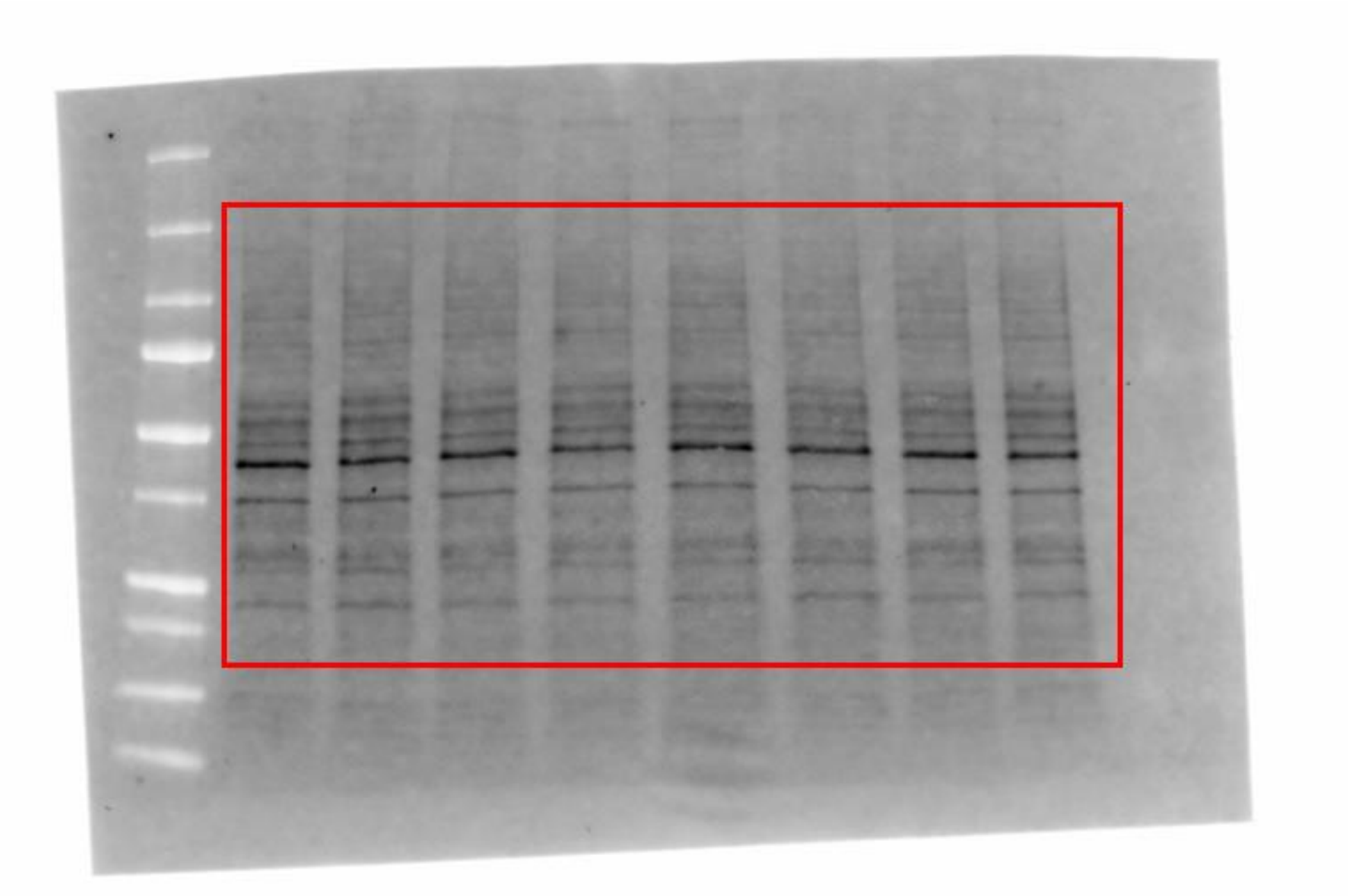

Full unedited gels for Fig. S8E

COL1A1 band

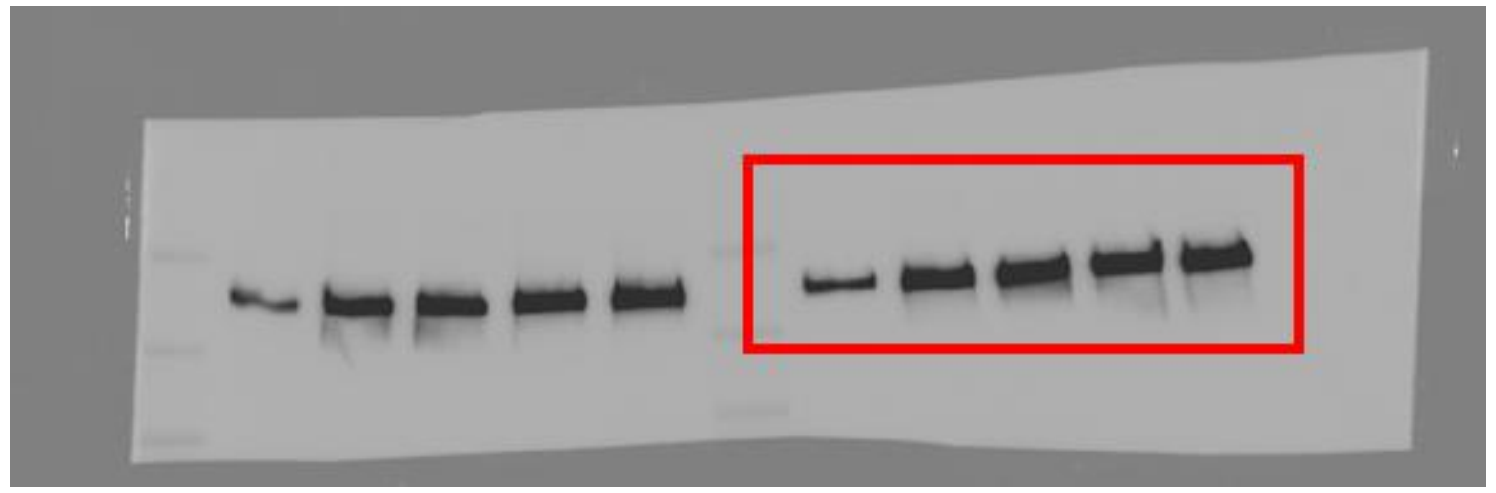

α-SMA band

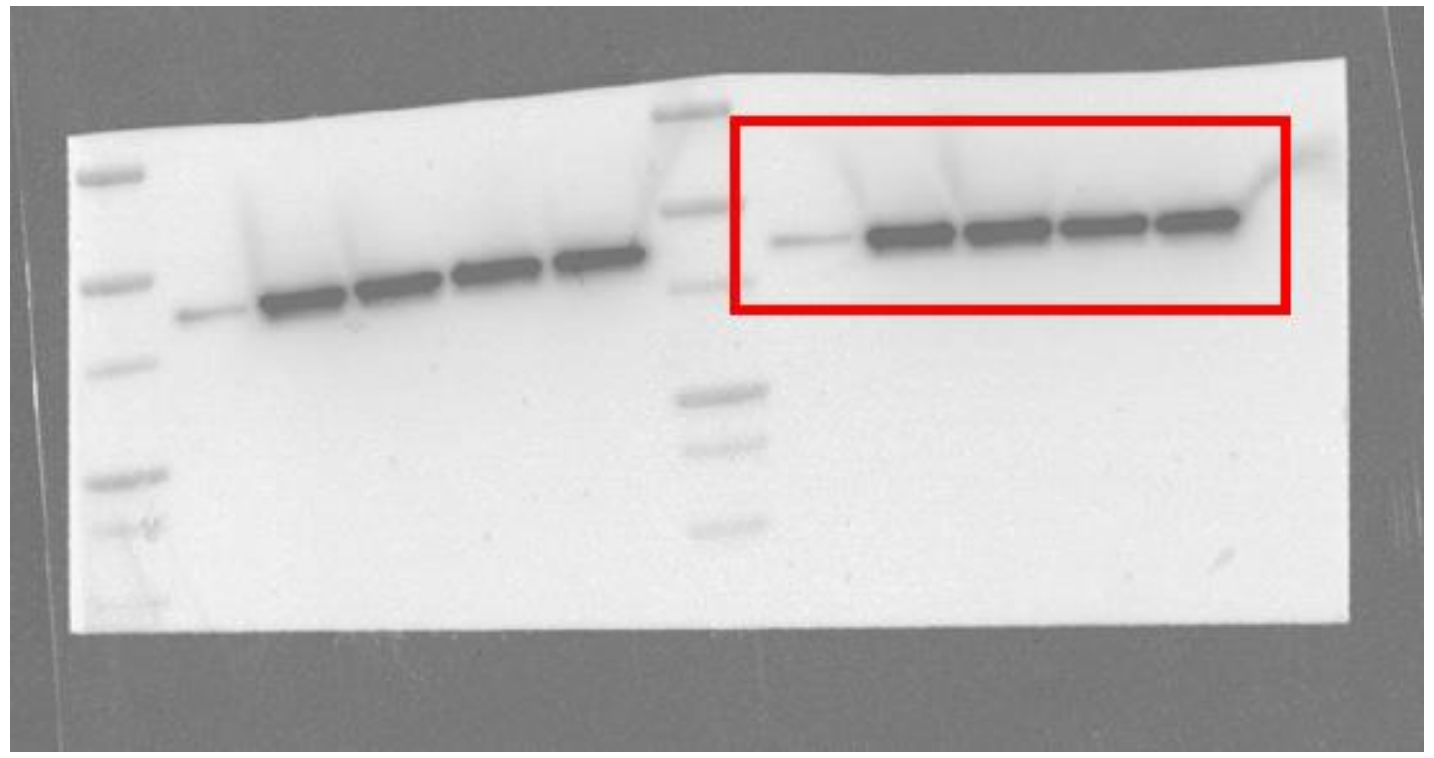

Total Protein

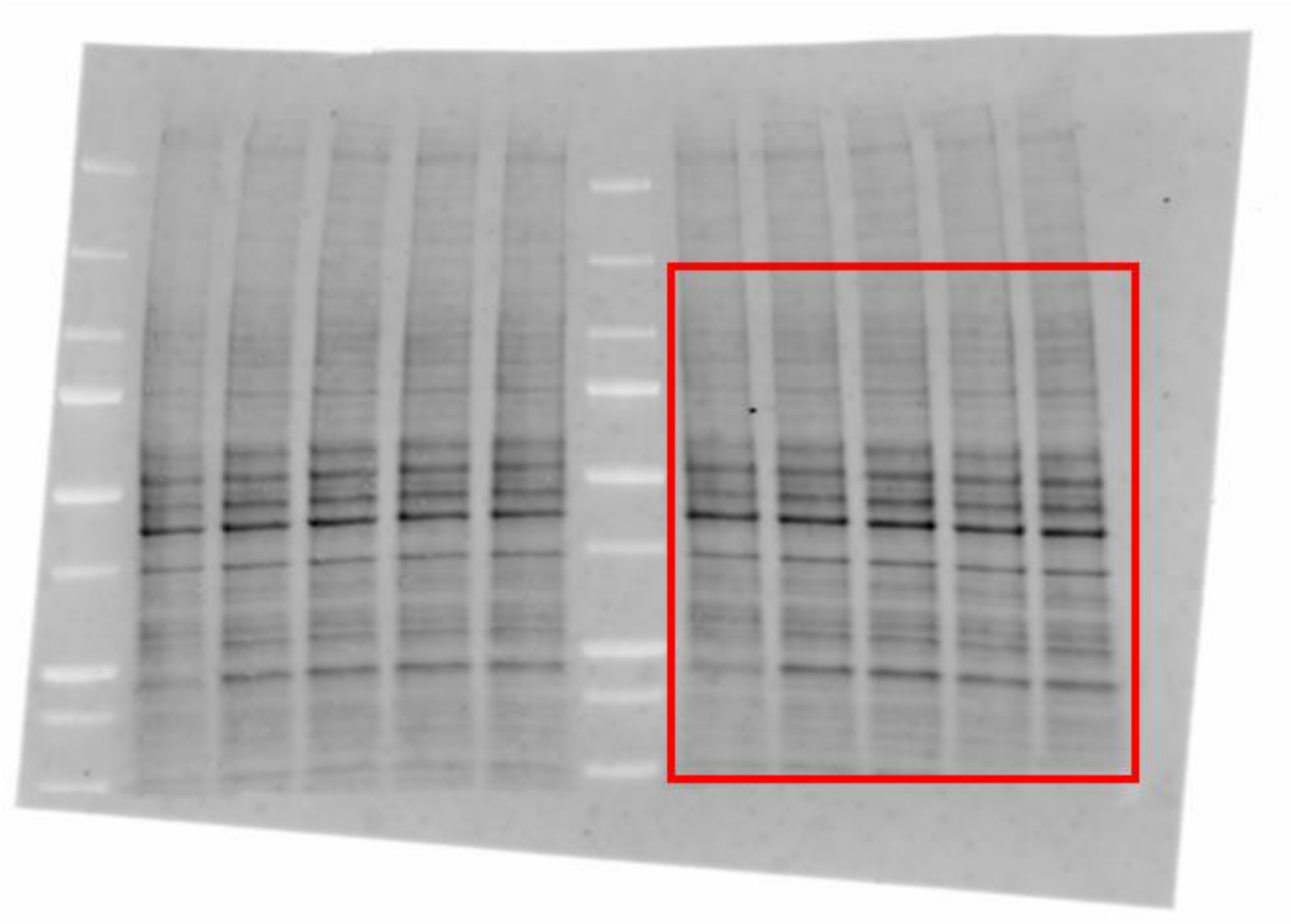

Supplement: Supplementary file 2 [file jciinsight-11-199449-s053.pdf]
